# Demystifying the catalytic pathway of *Mycobacterium tuberculosis* isocitrate lyase

**DOI:** 10.1038/s41598-020-75799-8

**Published:** 2020-11-03

**Authors:** Collins U. Ibeji, Nor Amirah Mohd Salleh, Jia Siang Sum, Angela Chiew Wen Ch’ng, Theam Soon Lim, Yee Siew Choong

**Affiliations:** 1 Institute for Research in Molecular Medicine (INFORMM), Universiti Sains Malaysia, 11800 Minden, Penang Malaysia; 2 Catalysis and Peptide Research Unit, School of Health Sciences, University of KwaZulu-Natal, Durban, 4041 South Africa; 3 Department of Pure and Industrial Chemistry, Faculty of Physical Sciences, University of Nigeria, Nsukka, 410001 Enugu State Nigeria

**Keywords:** Molecular modelling, Molecular biology

## Abstract

Pulmonary tuberculosis, caused by *Mycobacterium tuberculosis*, is one of the most persistent diseases leading to death in humans. As one of the key targets during the latent/dormant stage of *M. tuberculosis*, isocitrate lyase (ICL) has been a subject of interest for new tuberculosis therapeutics. In this work, the cleavage of the isocitrate by *M. tuberculosis* ICL was studied using quantum mechanics/molecular mechanics method at M06-2X/6-31+G(d,p): AMBER level of theory. The electronic embedding approach was applied to provide a better depiction of electrostatic interactions between MM and QM regions. Two possible pathways (pathway I that involves Asp108 and pathway II that involves Glu182) that could lead to the metabolism of isocitrate was studied in this study. The results suggested that the core residues involved in isocitrate catalytic cleavage mechanism are Asp108, Cys191 and Arg228. A water molecule bonded to Mg^2+^ acts as the catalytic base for the deprotonation of isocitrate C(2)–OH group, while Cys191 acts as the catalytic acid. Our observation suggests that the shuttle proton from isocitrate hydroxyl group C(2) atom is favourably transferred to Asp108 instead of Glu182 with a lower activation energy of 6.2 kcal/mol. Natural bond analysis also demonstrated that pathway I involving the transfer of proton to Asp108 has a higher intermolecular interaction and charge transfer that were associated with higher stabilization energy. The QM/MM transition state stepwise catalytic mechanism of ICL agrees with the in vitro enzymatic assay whereby Asp108Ala and Cys191Ser ICL mutants lost their isocitrate cleavage activities.

## Introduction

Isocitrate lyase (ICL) plays an important role in the metabolic processes of citric, methylcitric and glyoxylate cycles^1,2^. During the latent/dormant stage, *Mycobacterium tuberculosis* ICL converts isocitrate to glyoxylate and succinate for energy generation via the glyoxylate cycle^3^. Studies also showed that *M. tuberculosis* cannot survive in a latent TB model without ICL^4–6^. Therefore, ICL has become one of the major targets for latent *M. tuberculosis*. The structure of *M. tuberculosis* ICL in complex with the isocitrate metabolites (glyoxylate and succinate) that was solved in year 2000^7^ has enabled the study of ICL^8–11^, as well as to search for possible inhibitors for ICL^12–14^.

The inhibition mechanism of the ICL family has been proposed from experimental findings^15–18^. However, the actual process of isocitrate cleavage/metabolism remains unclear. The mechanism of isocitrate cleavage was reported to be involved in the deprotonation of isocitrate C(2)–OH group which resulted in the cleavage of the C(2)–C(3) bond trailed by the protonation of the carbanion^15,19^ that progresses towards the C(3) position. Two mechanisms have been proposed for the cleavage of isocitrate. The first proposed mechanism stated that two water molecules adjacent to Mg^2+^ may be involved in the deprotonation of isocitrate C(2)–OH group that leads to the formation of an anionic intermediate^15^. The orientation and specific distances from the substrate hydroxyl group (C(2)–OH) also suggested two water molecules as the candidates to act as the catalytic base (removing protons; one close to Asp108 and the other to Glu182). The deprotonations from C(2)–OH is then followed by the transfer of proton to either Asp108 (Asp58 of 2-methylisocitrate lyase, MICL) or Glu182 (Glu115 of MICL). Cys191 or Asp87 will then act as the catalytic acid in the protonation of the C(3) atom of the cleaved isocitrate. The catalytic acid (also known as general acid) is referred to an amino acid that participates in the mechanism of hydrolysis responsible for protonating (adding a proton) the succinate leaving group in the ICL cleavage mechanism. The second proposed mechanism involved Tyr43 as a catalytic base in the transfer of proton to His114 bonded to water molecules, resulting in the shuttling of a proton from isocitrate C(2)–OH group to Glu115^17,18^. Besides, Arg161 and Tyr89 of MICL (Arg 228 and Tyr43 in ICL) were also proposed as the unidentified catalytic base^16,20^.

The hybrid quantum mechanics/molecular mechanics (QM/MM) approach has been used to gain insight into the reaction mechanism of enzymes^21–23^. The QM/MM approach has also been applied to understand the catalytic mechanisms for lyases such as aspartate ammonia lyase^24^, N-acetylneuraminic acid lyase^25^, α-1,4-glucan lyase^26^, (*R*)-hydroxynitrile lyase^27^ and 2,3-dimethylmalate lyase (DMML)^16^. Studies on IMP dehydrogenase, pectate/pectin lyases, fumarate reductase, and l-aspartate oxidase^28^, as well as citrate synthase^29^ suggested that Arg could be the catalytic base while calculations on DMML^16^ and pyruvate formate-lyase^30^ proposed that Cys as the catalytic acid. However, the identity of the catalytic base involved in the deprotonation of C(2)–OH is yet to be resolved.

Herein, we applied QM/MM (ONIOM) approach to gain a better understanding on the mechanism involved in the breaking of isocitrate into glyoxylate and succinate. We explored the two proposed pathways by reported experimental works and identified the probable acidic and basic residues involved in the mechanistic process. This, in turn could be useful in providing computational models for the design of new anti-TB drugs.

## Results

**Figure 1:**
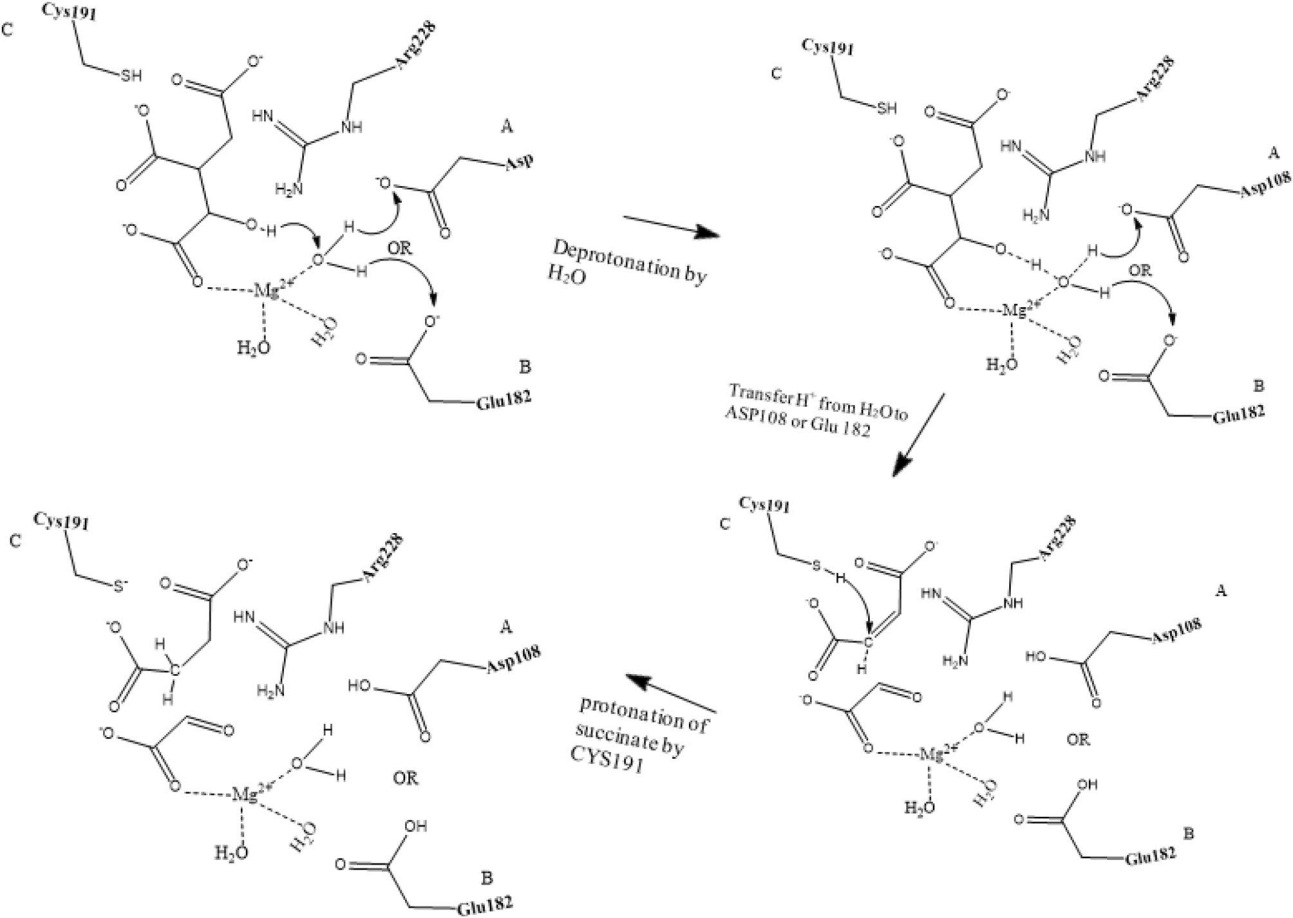
Possible catalytic mechanism of *M. tuberculosis* isocitrate lyase (ICL) for an acid/base-catalyzed conversion of substrate (isocitrate) to metabolites (glyoxylate and succinate). Step 1: Transfer of proton to either (**A**) Asp108 or (**B**) Glu182; Step 2: Proton transfer from Cys191 SH group to succinate C(3).

**Figure 2:**
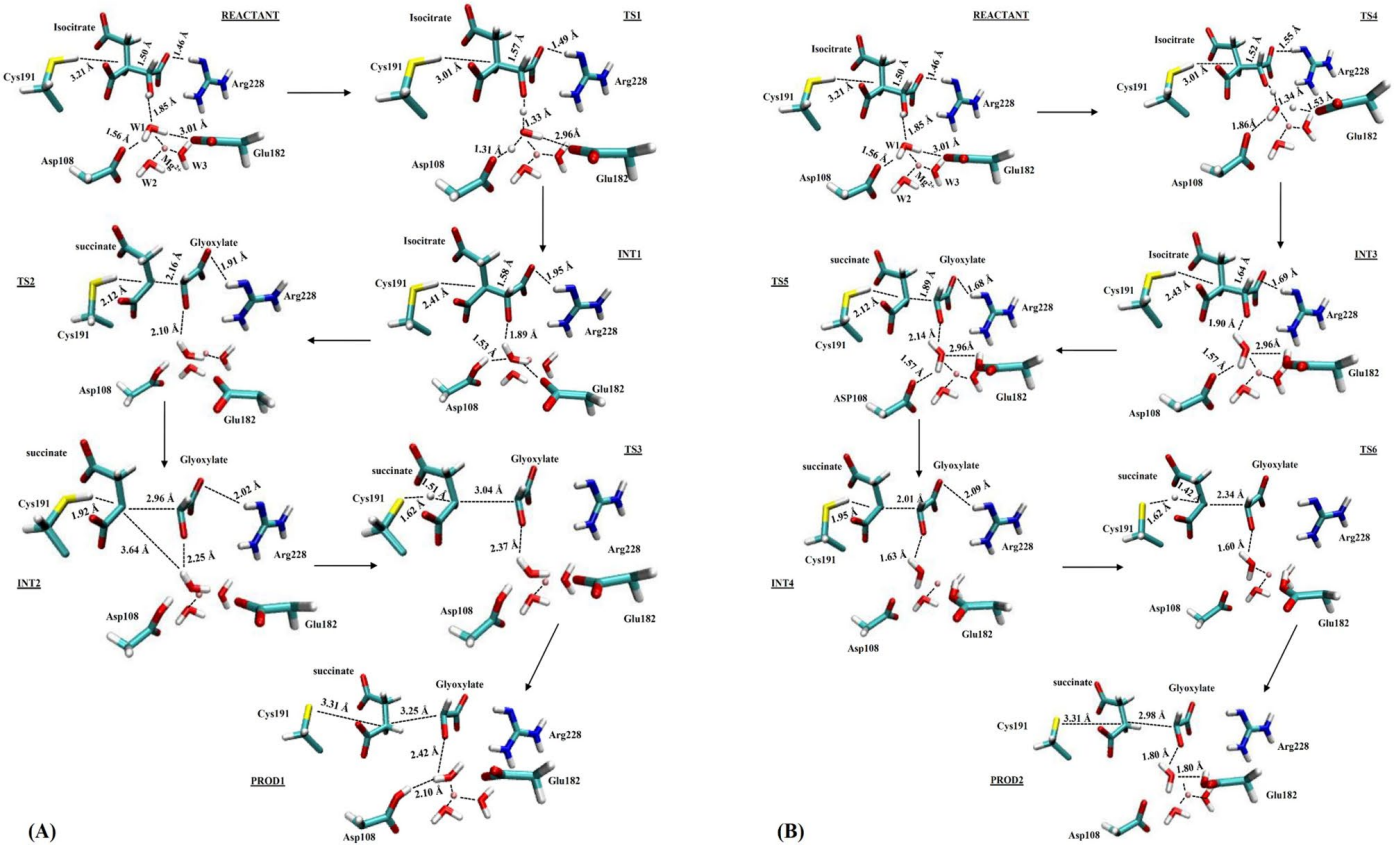
Graphical diagram of QM/MM stationary points at the M06-2X/6-31+G(d,p)/AMBER level of theory for possible (**A**) pathway I and, (**B**) pathway II**,** in the cleavage of isocitrate by *M. tuberculosis* isocitrate lyase (ICL).

**Figure 3:**
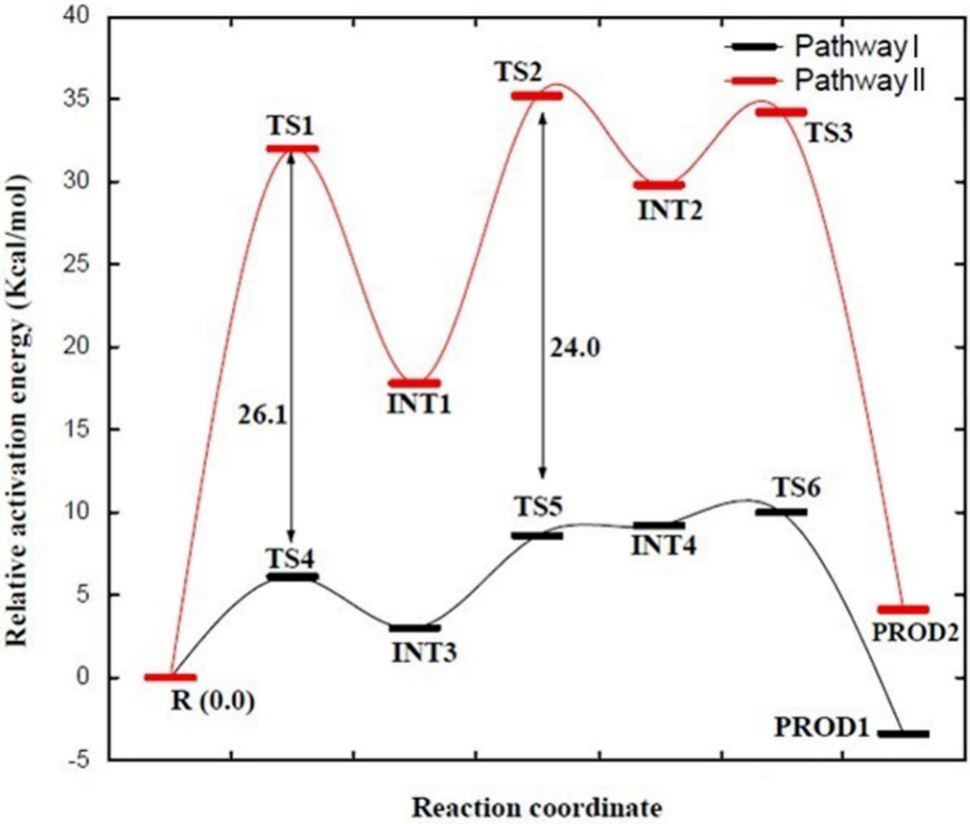
Relative Gibb free energy profile at (ONIOM) M06-2X/6-311++G(2d,2p) for the stepwise cleavage mechanism of isocitrate by *M. tuberculosis* isocitrate lyase (ICL).

**Figure 4:**
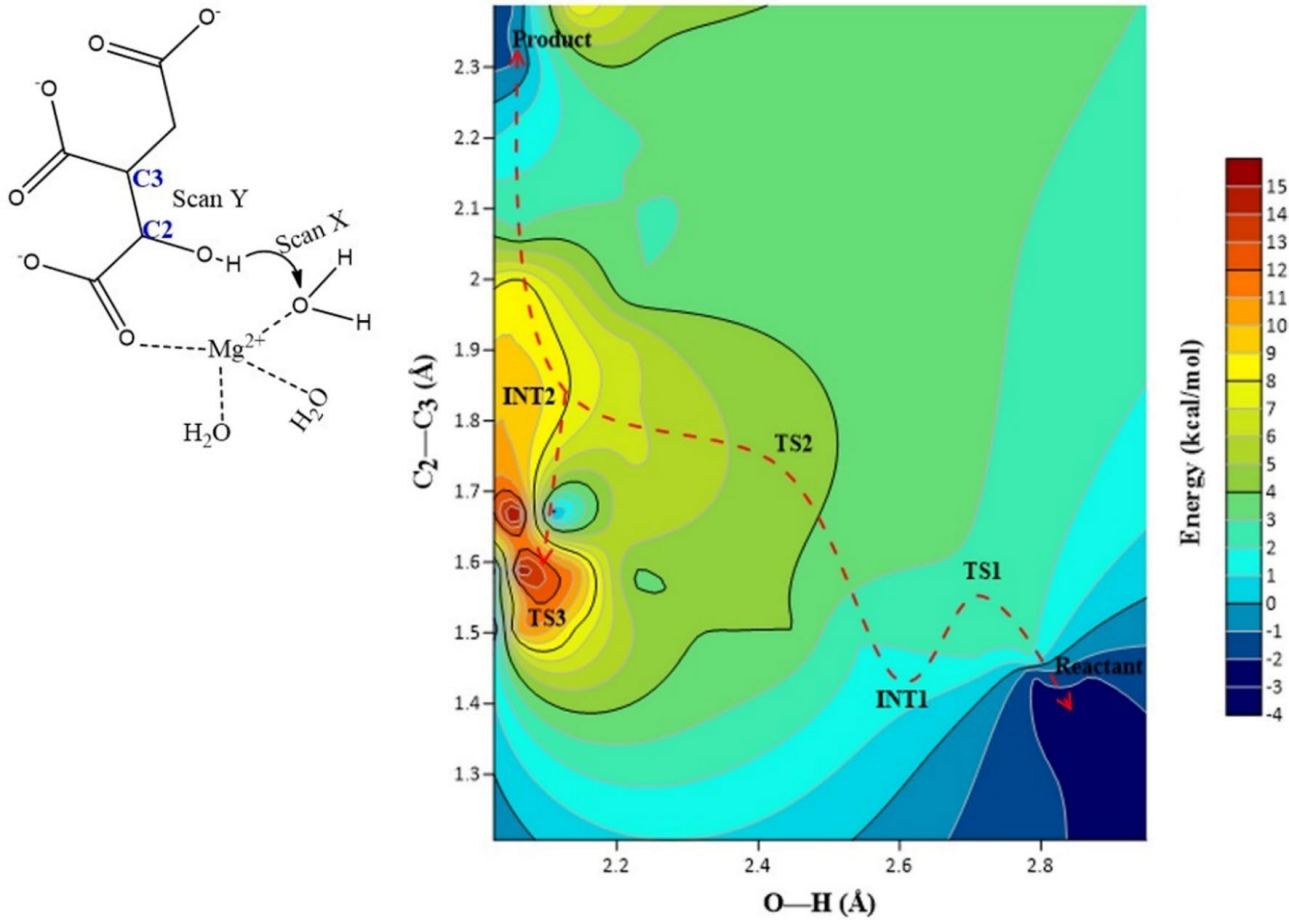
2D potential energy contour map showing a stepwise cleavage mechanism of isocitrate by *M. tuberculosis* isocitrate lyase (ICL) obtained from an ONIOM M06-2X/6-31+G(d,p)/AMBER scan calculation. TS is the transition state while INT is the intermediate.

The catalytic mechanism associated with the breakdown of isocitrate by *M. tuberculosis* ICL (Fig. 1) was explored using QM/MM (ONIOM) approach. In this work, we studied two different pathways (namely pathway I and II) that could lead to the metabolism of isocitrate (Figs. 1, 2). These pathways would involve two residues, one as the catalytic acid and the other as the catalytic base as observed experimentally^15^. The relative activation energies associated with the catalytic mechanism for pathways I and II are presented in Table 1 and Fig. 3. Single-point calculations with two additional density functionals (mPWB1W and ωb97XD) paired with 6-311++G (2d, 2p) in conjunction with electronic embedding were employed to explore the sensitivity of the applied functionals. These functionals have been reported to give accurate energetics for kinetics and thermodynamics^31^. The two-dimensional (2D) PES was performed to visualise the cleavage mechanism. The scan result suggested that the cleavage mechanism is a stepwise mechanism (Fig. 4). The figure showed that a concerted catalytic mechanism is unlikely since the deprotonation of isocitrate C(2)–OH and a proton transfer to the acceptor residue occurs before the elongation of the C(2)–C(3) bond and thereafter leading to bond breaking. This is consistent with the observation for the mechanism of DMML, a subclass of the lyase family^16^. The starting structure for the non-constrained transition state was obtained from the scan coordinate (Fig. 4).

**Table 1:** Reaction mechanism (relative) energy (kcal/mol) obtained from ONIOM using different density functionals at 6-311++G(2d,2p): Amber for the breakdown of isocitrate by *M. tuberculosis* isocitrate lyase (ICL).

	M06-2X ^a^		mPWB1W ^b^		ωb97XD ^c^	
Reaction path	∆E	∆G^‡^	∆E	∆G^‡^	∆E	∆G^‡^
**Pathway I**						
R	0.00	0.00	0.00	0.00	0.00	0.00
TS1	6.2	6.1	6.1	6.0	6.2	6.1
INT1	3.8	3.0	3.6	2.9	3.9	3.1
TS2	9.1	8.6	9.2	8.4	9.7	8.8
INT2	9.4	9.2	9.2	9.0	9.5	9.3
TS3	11.1	10.0	11.0	9.9	11.8	10.1
PROD1	− 2.1	− 3.4	− 2.2	− 3.5	− 2.9	3.8
**Pathway II**						
TS4	32.3	32.0	32.4	32.2	32.5	32.4
INT3	17.6	17.8	17.9	17.8	18.0	17.9
TS5	33.1	35.2	35.8	35.4	35.9	35.7
INT4	30.1	29.8	30.7	30.3	30.6	30.6
TS6	34.5	34.2	34.6	34.4	34.7	34.6
PROD2	5.3	4.1	4.5	4.2	4.6	4.1

^a,b,c^Relative (to reactant) total electronic energy (ΔE) and activation free energy (ΔG^‡^, with thermal correction) at B3LYP, M06-2X, ωB97X/6-311++G(d,p):AMBER//B3LYP/6-31G(d,p):AMBER level.

*R *reactant, *TS *transition state, *INT *intermediate, *PROD *product.

### Reaction mechanism for pathway I

The QM/MM reactant structure obtained from the restrained MD simulation is similar to the X-ray crystal structure with comparable key atomic distances (Supplementary Information Figure S1). The RMSD plot from the MD simulation is presented in Supplementary Information Figure S2. The ICL active site consists of a metal-dependent Mg^2+^ cofactor coordinated to three water molecules (namely W1, W2 and W3) with interatomic distances (Mg^2+^–O) of 1.96, 2.01 and 2.16 Å, respectively. It is also evidenced from the structural data that the substrate (isocitrate) for ICL is activated for deprotonation through the binding of Mg^2+^ to isocitrate C(2)–O carboxyl atom. The substrate interacts strongly with Arg228 with a short hydrogen bond distance of 1.46 Å corresponding to O–H atom (Fig. 2A). As suggested by X-ray experiment data^15^, two possible water molecules may mediate the deprotonation of isocitrate C(2)–OH based on the orientation and distance to the C(2)–OH group of isocitrate. FigureS 1 and 2A show that although water molecules W1 and W2 are coordinated to Mg^2+^, the position of W1 is closer and oriented to transfer the proton from isocitrate C(2)–OH with the interatomic distance of 1.85 Å. The proposed catalytic base identified as Cys191 is close enough to isocitrate C(3) with an interatomic distance of 3.21 Å. Further details on the optimized geometries of the high layer (QM) ONIOM model and the redundant coordinate of isocitrate lyase can be found in the Supplementary Information Figure S3.

### The function of water molecule (W1) in pathway I

**Figure 5:**
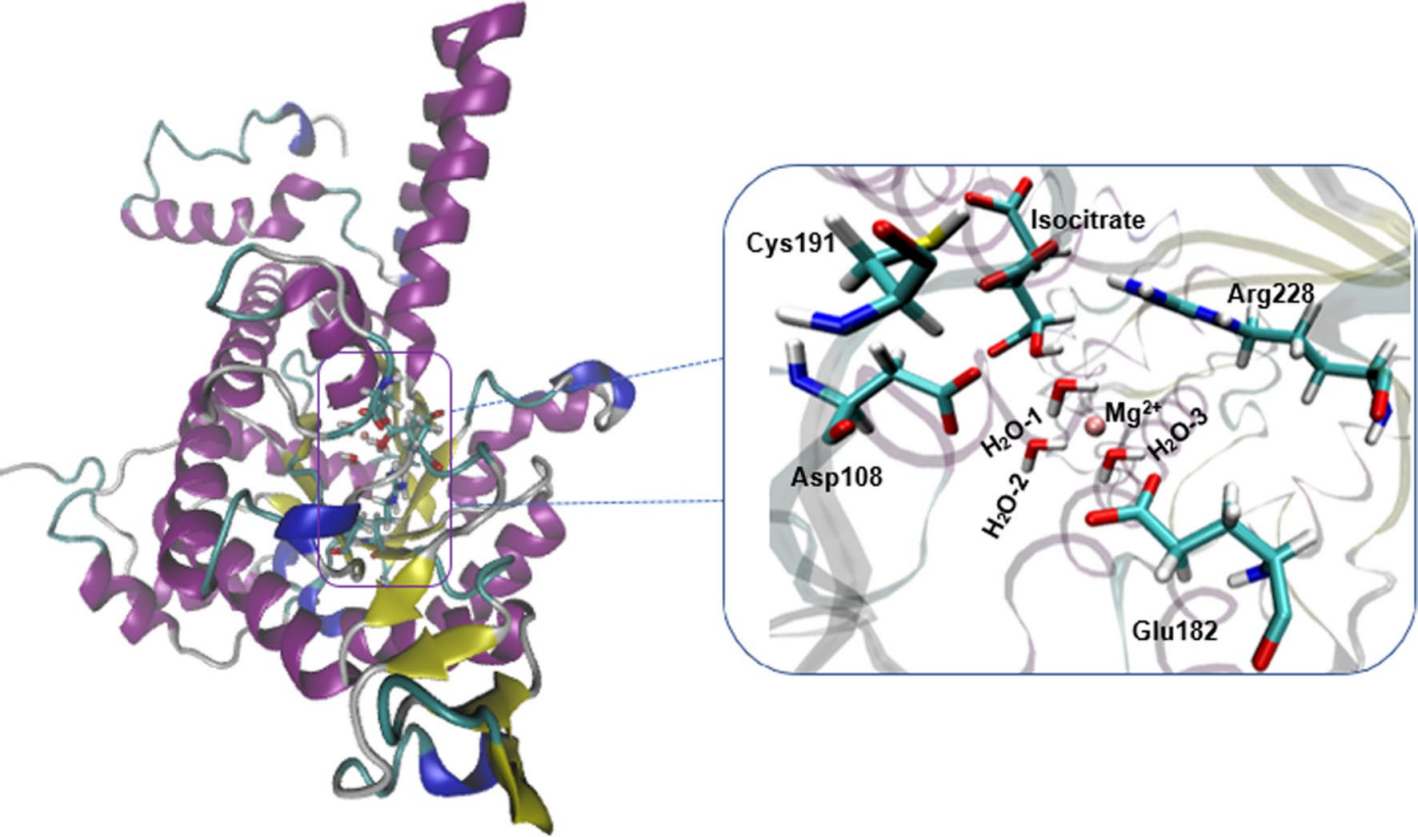
3D structural depiction of the QM/MM ONIOM model in the study of *M. tuberculosis* isocitrate lyase (ICL) catalytic pathway. The optimized the geometries of the high layer (QM) at the B3LYP/6-31+G(d,p) is in stick presentation while outside the box is the MM region in ribbon presentation.

It has been previously reported that water may act as a catalytic base in the cleavage mechanisms^15^. As shown in Fig. 1, a catalytic base is responsible for the isocitrate activation via proton abstraction. It was observed from the crystal structure^15^ that there are three water molecules (W1, W2, W3) coordinated to Mg^2+^ (Figs. 4, 5). These water molecules are oriented towards the hydroxyl group attached to isocitrate C(2) atom with W1 having the shortest distance of 1.85 Å , that is close enough to cause deprotonation of the isocitrate OH group. The energetics of the reaction step were studied and a concerted process was observed for the deprotonation of the hydroxyl group attached to isocitrate C(2) atom with concurrent deprotonation of W1 (Fig. 2A). The relative Gibbs free energy of activation (∆G^‡^) associated with this state (TS1) was observed to be 6.1 kcal/mol (Table 1). An intermediate (INT1) is formed in the process by the transfer of a proton from W1 to Asp108 with a distance of 1.31 Å, similar to the deprotonation distance of isocitrate C(2)–OH. The ∆G^‡^ associated with the intermediate state is about 3 kcal/mol lesser than TS1. We also evaluated if Arg228 can function as a catalytic base to deprotonate isocitrate C(2)–OH. However, the data showed that isocitrate becomes unstable as the orientation changed, resulting in an elongated distance beyond 5 Å between isocitrate C(2)–OH and Arg228. This suggests that W1 is more likely to act as the catalytic base in the mechanism of the reaction.

### The function of Asp108 in pathway I

It was proposed that water molecules may shuttle proton from the hydroxyl group of isocitrate C(2) via Asp108 or Glu182 (Fig. 1)^15^. To demonstrate Asp108 is key to this process, *S*. *enterica* Asp108 (Asp58 in MICL) was substituted with Ala and the result showed that Asp108 was essential in the catalytic outcome^15^. The 2D potential energy surface (PES) showed that the proton from C(2)–OH group was transferred to Asp108 (INT1) before the cleavage of the C2–C3 bond (TS2). Asp108 was found to form a perturbed bond with Mg^2+^ by direct coordination and the energetics for the mechanistic process showed that the transfer of a proton from W1 to Asp108 was preferred and favoured compared to Glu182 (Table 1; Fig. 2B). In this barrierless process, it was also observed that the distance between Cys191 and isocitrate C(3) has reduced considerably from 3.01 to 2.41 Å. This may indicate a characteristic hydrogen bonding between the electron-rich isocitrate C(3) atom and Cys191 thiol group. Thus, suggesting that Cys191 could be the catalytic acid in isocitrate cleavage process. Further analysis of the energetics (INT1) using a different functional (mPWB1W) revealed that the process is barrierless with lower ∆G^‡^ of 2.9 kcal/mol.

### The role of Arg228 in pathway I

In DMML, Arg161 (corresponding to Arg228 of *M. tuberculosis* ICL) was reported to be potentially responsible for the deprotonation of isocitrate C(2)–OH which leads to the cleavage of isocitrate C(2)–C(3) bond^16,20^. However, our study showed alternative findings as ICL Arg228 is already in the protonated state and could not serve as a proton shuttle from isocitrate C(2)–OH that leads to the isocitrate cleavage. But our observation is similar to MICL experimental findings^15^ whereby there is no basis for Arg228 to mediate a proton transfer based on its protonated state since it only aids in intermediate stabilization. In this study, step 3 of the mechanism involves the breaking of isocitrate C(2)–C(3) bond and it was observed that Arg228 plays an important role in this step. In the reactant stage, Arg228 forms a strong bond with the carboxylate group of the substrate (Fig. 2A) which stabilizes the substrate and aids the intermediate stabilization. After the deprotonation of the C(2) atom hydroxy group, Arg228 pulls out from the substrate with an elongated bond distance from 1.49 Å (reactant TS1) to 1.91 Å (TS2) which further resulted in the destabilization of the substrate thus leading to the cleavage of the C(2)–C(3) bond (TS2). In this step, the interatomic distance between C(2) and C(3) atom changes from 1.50 to 2.16 Å (reactant to TS2).

### The function of Cys191 in pathway I

In MICL, Cys123 (corresponding to Cys191 in *M. tuberculosis* ICL) was found to show significant effect on the stereochemistry and mechanism of the isocitrate C(2)–C(3) bond cleavage^15^. The cleavage of isocitrate resulted in the formation of a carbanion which is followed by protonation of this intermediate by Cys191. Our calculation showed that the carbanion-like intermediate transition state (INT2) at the C(3) position following the breaking of the C(2)–C(3) bond is characterised by an activation energy of about 3 kcal/mol higher than the INT1. The proton transferred from Cys191 thiol group to the intermediate with a ∆G^‡^ of 10.0 kcal/mol (TS3). From the crystal structure, the most probable candidate to play the role of the catalytic acid would be Cys191 and the water molecule bound to Mg^2+^ based on their p*K*_a_ and distances (Supplementary Information Figure S1). In addition, the interatomic distance between Cys191 SH group and succinate C(3) atom in INT2 is 1.92 Å compared to 3.64 Å between succinate C(3) atom and W1. Likewise, either Asp108 or Arg228 were too far (> 4 Å) from succinate C(3) atom to donate a proton. Therefore, Cys191 would be the most suitable candidate to serve as a catalytic acid in the cleavage of isocitrate by ICL.

### Alternative pathway II

As mentioned earlier, the experimental data showed that water molecules may shuttle proton from the hydroxyl group of the isocitrate C(2) atom via Asp108 or Glu182^15^. Therefore, we also considered another scenario whereby the deprotonated atom was transferred to Glu182 (Fig. 2B). The QM/MM calculations revealed that the transfer of a proton to Glu182 could occur but would require a higher activation energy (~ 26 kcal/mol) compared to the transfer of proton to Asp108. The ∆G^‡^ for the intermediate step (INT3) is also higher compared to the protonation stage of Asp108, indicating that concerted proton transfer step in Glu182 is less favourable due to a higher energy barrier. The bond breaking step of isocitrate C(2)–C(3) could be slower based on the interatomic distance compared to pathway I (TS2). The activation energy of the rate-determining step associated with C(2)–C(3) bond breaking is characterized by a higher energy barrier of 35 kcal/mol compared to the pathway I. The formation of the products (glyoxylate and succinate) was also observed to have a high activation energy. In addition, the analysis of the entropy associated with the product formation step revealed that the products formed in pathway II possesses lower entropy compared to that in pathway I (Supplementary Information Table S1). Second order perturbation theory calculation to analyse the natural bond orbital was also performed to further study the stabilization energy and the intermolecular charge transfer associated with TS1 and TS4.

### Natural bond orbital (NBO) analysis

**Figure 6:**
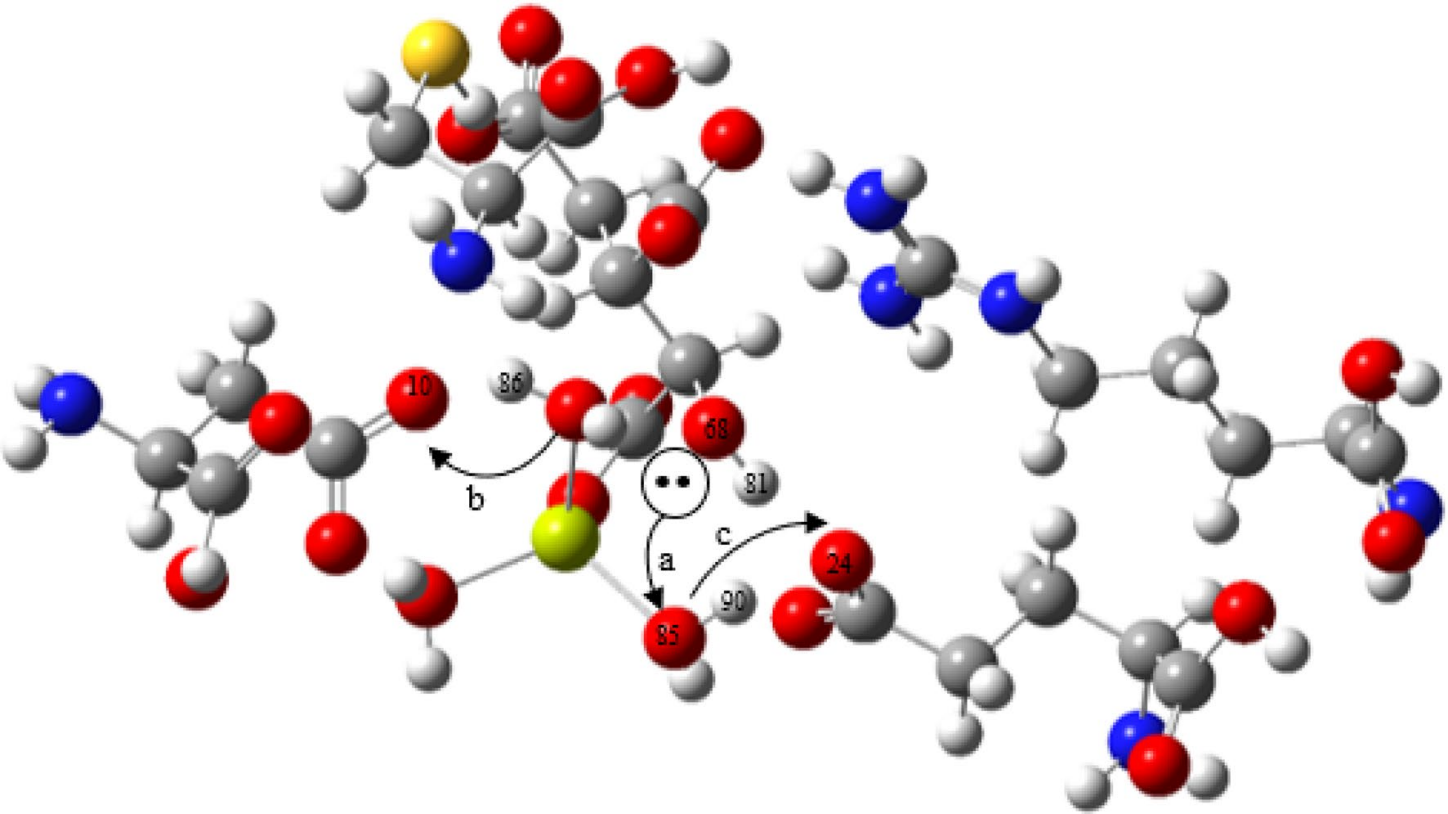
The depiction of electrons transfers for isocitrate-ICL complexes derived from second-order perturbation theory of NBO analysis. The curved arrows (a, b and c) illustrate the direction of charge transfer from lone pair to antibonding (LP → σ*).

NBO analysis from the second order perturbation theory calculation demonstrates that higher stabilization energy (E^2^) values indicate stronger intermolecular interactions with a higher charge transfer between electron-donor and electron-acceptors due to hyperconjugation^32–34^. Table 2 shows that the stabilization energy E^2^ for the deprotonation of the isocitrate C(2)–OH (O_68_) for pathway I represented as TS1 is 64.4 kcal/mol, indicating more electron charge transfer from a lone pair LP(O_68_) to O_85_ of W1. For pathway II, the E^2^ value was lower compared to pathway I. A precise contrast between transition states TS1 involving the transfer of proton to Asp108 and TS4 (proton transfer to Glu182) revealed that the pathway I transition state is favoured with higher stabilization energy. A similar comparison has been shown in the literature for other catalytic mechanism involving enzymes^21^. This calculation demonstrates that higher intermolecular interaction and charge transfer takes place in pathway I, involving the transfer of proton to Asp108 (Fig. 6).

**Table 2:** Second-order perturbation stabilization energies (kcal/mol) corresponding to the main intermolecular charge transfer interaction (Donor → Acceptor) obtained at M06-2X/6-311++g(d,p) for *M. tuberculosis* isocitrate lyase (ICL) for pathway I and II transition states.

Donor	Acceptor	E^2^ (kcal/mol)
**TS1**		
LP(O_68_)	σ* (O_85_–H_81_)	64.4
LP(O_85_)	σ* (O_10_–H_86_)	78.8
**TS4**		
LP(O_68_)	σ* (O_85_–H_81_)	31.1
LP(O_85_)	σ* (O_24_–H_90_)	24.6

### *M. tuberculosis* ICL cloning, expression and purification

**Figure 7:**
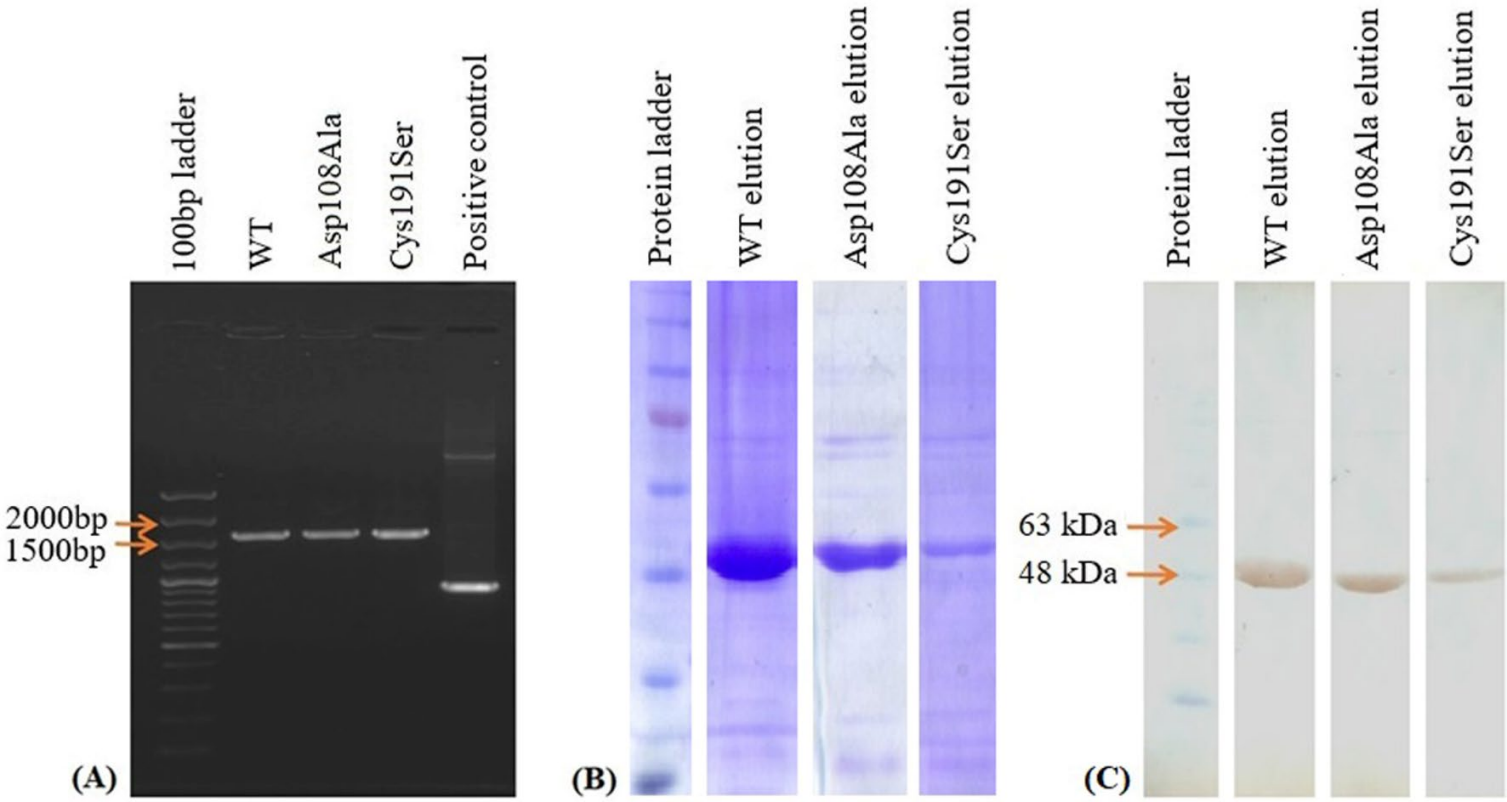
(**A**) The gel electrophoresis of colony PCR from wild type (WT), mutant type (MT) Asp108Ala and Cys191Ser *M. tuberculosis* isocitrate lyase (ICL) gene. The plasmid size for each sample is about 1600 bp. (**B**) The Coomasie Blue stained SDS-PAGE gel and (**C**) Western Blot of the purified WT, MT Asp108Ala and Cys191Ser *M. tuberculosis* ICL. The purified ICL is about 50 kDa. Full-length gels and blot are presented in Supplementary Information Figure S4.

Extraction of pET28α plasmid containing the *M. tuberculosis* ICL gene was used due to the availability of the lac operon for inducible expression of the ICL protein. Site directed mutagenesis of the ICL was done to introduce point mutations at Asp108Ala and Cys191Ser. A total of 6 colonies from both mutated ICL plates were randomly picked for colony PCR. The amplified colony PCR product of the mutant (MT) and wild type (WT) ICL resolved at a correct DNA band size of 1600 bp by electrophoresis (Fig. 7A). The sequencing results confirmed successful introduction of the desired point mutations in the ICL gene. The plasmids were then used for the expressions of the WT and MT ICL. The expressed ICL was extracted and purified from overnight culture using HIS-tagged purification method. Different elution and washing step were applied during the purification stage to ensure the proteins were purified to ~ 95% purity. The purified ICL was tested on SDS-PAGE to confirm and validate the protein size from the expression. The WT and MT protein extracts have the same size of approximately 50 kDa (Fig. 7B). To confirm the purified bands were indeed that of the ICL, Western Blotting analysis was carried out. The Western Blot analysis showed a distinct band at approximately 50 kDa (Fig. 7C). From the gel and blot analysis, the yield of the Cys19Ser mutant appeared to be lower than the Asp108Ala mutant. The solubility of the WT protein provided a higher yield in comparison to the two mutants.

### Enzymatic assay

**Figure 8:**
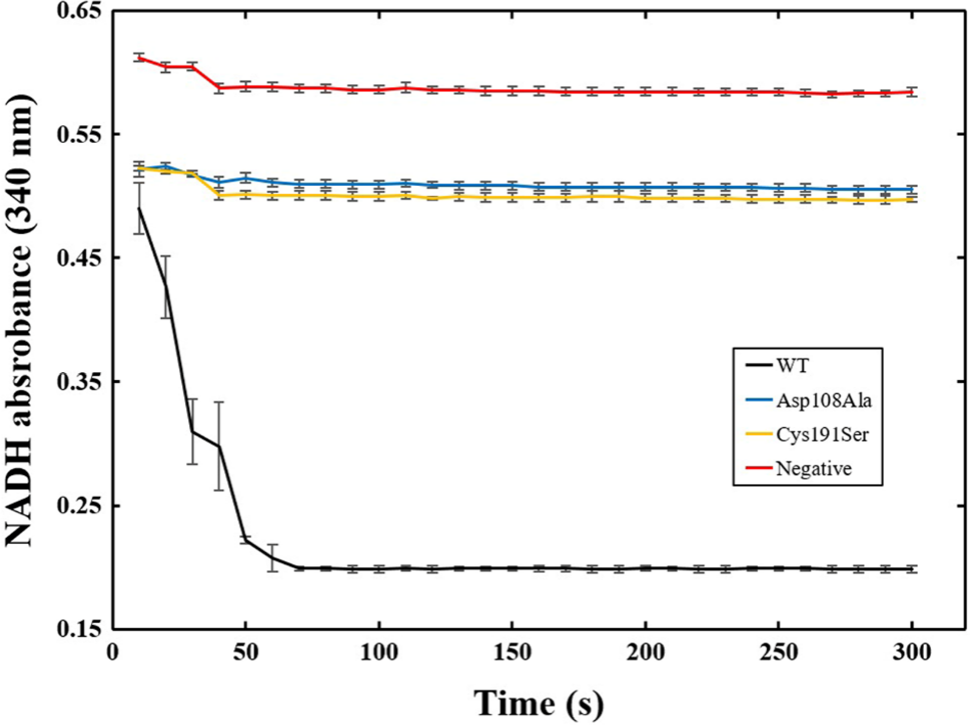
The NADH absorbance at 340 nm throughout 300 s reaction time for WT, MT Asp108Ala and Cys191Ser *M. tuberculosis* isocitrate lyase (ICL).

Enzymatic assay of ICL was performed to validate and differentiate the functionality of the purified WT and MT ICL with isocitrate as the substrate. The ICL activity determination has been previously described^35^. In general, an active ICL will cleave isocitrate into glyoxylate and succinate as the metabolites. With the presence of lactate dehydrogenase, NADH will be oxidised, followed by the reduction of glyoxylate to glycoxylate. The formation of glycoxylate will then reduce the concentration of NADH in the sample thus leading to a decreasing absorbance pattern of NADH which can be detected at 340 nm^36^. In this experiment, both MT ICL clones were considered inactive as the absorbance reading of NADH remained constant compared to WT ICL which underwent rapid absorbance decrease from ~ 0.5 to ~ 0.2 in 300 s (5 min) reaction time (Fig. 8).

## Discussion

The mechanistics involved in the cleavage of isocitrate is of interest due to its potential application for drug development. An earlier experiment proposed that water may act as a catalytic base since three water molecules labelled as W1, W2, W3 are coordinated to Mg^2+^ oriented towards the hydroxyl group attached to isocitrate C(2) atom^15^. We observed through the energetics with lower activation energy (TS1) and a concerted process, water molecule W1 functioned as the catalytic base associated with the deprotonation of isocitrate C(2)–OH.

Our investigation also showed that the isocitrate cleavage mechanism follows a stepwise process and the results suggests that proton shuttling from the hydroxyl group of isocitrate C(2) is favourably transferred to Asp108 at a lower activation energy of 6.2 kcal/mol instead of Glu182. The rate-determining step of the reaction mechanism is associated with the breaking of the isocitrate C(2)–C(3) bond followed by a proton transfer from Cys191, which was suggested as the catalytic acid. It was also observed that Arg228 or Asp108 was found to be far from the C(3) atom to donate a proton to the cleaved isocitrate, thus suggesting that Cys191 would be the most suitable candidate to serve as the catalytic acid in the cleavage of isocitrate by ICL. In this study, step 3 of the mechanism involves the breaking of isocitrate C(2)–C(3) bond and it was observed that Arg228 plays an important role in this step by forming a strong bond with the carboxylate group which stabilizes the substrate. After the deprotonation of isocitrate C(2) hydroxyl group, Arg228 detaches from the substrates with a lengthened bond length making it difficult to contribute significantly to the break down of substrate.

The pathway II which involves the proton transfer from W1 to Glu182 was found to be with a high energy barrier making Glu182 unlikely to be involved in this catalytic mechanism. A further study of the entropy calculation showed that pathway I which involves water molecule W1 as the catalytic base had a more negative entropy compared to pathway II, indicating that pathway I mechanistic route is more favourable. Second order perturbation theory calculation was also performed to further study the stabilization energy and the intermolecular charge transfer associated with TS1 and TS4. The stabilization energy *E*^2^ derived from second perturbation theory calculation also unveiled that TS1 of the pathway I had stronger intermolecular charge transfer compared to TS4 (pathway II).

The theoretical calculation in this work was also supported by in vitro investigation. The Asp108Ala and Cys191Ser ICL mutants were non-reactive and did not generate the metabolites indicating the importance of Asp108 and Cys191 to the enzyme function. However, WT ICL remained active in the assay for the production of the metabolites. Therefore, the enzymatic assay further indicates the importance of both Asp108 and Cys191 to ensure the functionality and catalytic activity of *M. tuberculosis* ICL.

## Conclusion

This study considered the catalytic mechanism for the cleavage of isocitrate by *M. tuberculosis* ICL using QM/MM approach. We explored the ill-defined residues involved in the cleavage mechanism which was unclear from experimental findings. Our investigation showed that the cleavage mechanism follows a stepwise process and the proton shuttle from the hydroxyl group of the isocitrate C(2) atom is favourably transferred to Asp108 instead of Glu182 with a lower activation energy of 6.2 kcal/mol. The rate-determining step of the reaction mechanism is the isocitrate C(2)–C(3) bond break that is followed by a proton transfer from Cys191 which was suggested as the catalytic acid. The pathway II that involves the proton transfer from W1 to Glu182 was found to have a high energy barrier making Glu182 unlikely the residue to be involved in this catalytic mechanism. The stabilization energy *E*^2^ derived from second perturbation theory calculation also unveiled that that TS1 of the pathway I had stronger intermolecular charge transfer compared to TS4 (pathway II). The revelation of the residues involved in *M. tuberculosis* ICL mechanism of inhibition could therefore provide better insights for future designs of new anti-tuberculosis drugs.

## Materials and methods

### Computational details

#### Structure preparations

The X-ray crystal structure of *M. tuberculosis* ICL with PDB id 1F8I^7^ (glyoxylate and succinate as the ligands) and isocitrate with PDB id 1XG4^15^ (MICL with isocitrate as the ligand) were aligned using Pymol^37^ program. The ligands of 1F81 (glyoxylate and succinate) and MICL of 1XG4 were then removed to generate the starting structure of isocitrate in complex with ICL. The Ser191 point mutation of ICL (in 1F81) was reverted to WT (Cys191). The three catalytic water molecules coordinated to Mg^2+^ in 1F8I was kept according to the possible catalytic mechanisms study^38^.

#### Molecular dynamics (MD) simulation

Molecular dynamics (MD) simulation was conducted using Amber 14^39^ with ff99SB force field^40^ for ICL and GAFF^41^ for isocitrate. The protonation states of ICL were assigned based on the pK_a_ values computed by the empirical PropKa web server^42^. The complex was immersed in a 10 Å TIP3P^43^ cubic water box and Na^+^ as counter ions were added to neutralize the system. Prior to MD simulation, two-stage geometric minimization was performed with 2500 steps of steepest decent followed with 2500 of conjugated gradient to relax steric clashes and closed contacts. The first stage minimization involved a constraint of 500 kcal/mol/Å^2^ while the second stage minimization did not involve any restraints. The system was then heated slowly to 300 K under the NVT ensemble for 500 ps using the Langevin thermostat^44^ with a collision frequency of 1 ps^−1^. A 10 ns of protein-retrained equilibration MD with force constraint of 10 kcal/mol/Å^2^ was first performed. This was followed by 25 ns MD production run with 2 fs time step without harmonic restraints in the periodic boundary condition under the NPT ensemble, 300 K and 1 atm. Partial Mesh Ewald (PME) algorithm^45^ was employed to calculate long range electrostatic interactions while 12 Å cutoff was set for van der Waals force. All bonds involving hydrogen atoms were constrained using SHAKE algorithm^46^. Analysis of RMSD was performed on ICL backbone C_α_ atoms.

#### ONIOM QM/MM setup

The starting structure for QM/MM calculation was obtained for the last snapshot (with RMSD of 1.8 Å compared to the crystal structure) of the 25 ns MD production run. Two-step minimizations were performed as described in the MD simulation to remove unfitting distortions. Truncation of the system was performed by removing water molecules beyond 15 Å from isocitrate. A two-layered ONIOM method^47–49^ in Gaussian 09^50^ that was widely used in enzymatic systems^21,51–53^ was applied in this study. The ONIOM QMM/MM system was divided into two layers: the high layer (QM) and the low layer (MM) (Fig. 5). The QM region consisted of 102 atoms and 6 link hydrogen atoms employed to saturate dangling bonds and to treat the boundary between QM/MM region, while the rest was included in the MM region. To circumvent spurious deviations and fluctuations in the geometries, the QM/MM regions, residues and water molecules within 6 Å around the active site were fully optimized while others were held fixed (2443 atoms in the MM region). Partial charges for the high layer was calculated using the restrained electrostatic potential (RESP) charge fitting^54^. To obtain a better understanding of the proposed reaction mechanism, potential energy surface (PES) for bond breaking (C2–C3) and deprotonation of the C(2)–OH group reaction coordinate scan were explored using ONIOM implemented in Gaussian 09. In order to obtain a better understanding of the proposed reaction mechanism, potential energy surface (PES) scan was explored using ONIOM implemented in Gaussian 09. The PES scan is a two dimensional single scan which involves the C(2)–C(3) and the deprotonation of C(2)–OH group. When two coordinate scan is done simultaneously, it is called a 2D scan. The scan was performed prior to the final optimization and the starting structures for the transition state (TS) was obtained from the 2D scan (Fig. 4). Density functional theory (DFT) was applied in for QM optimization. M06-2X functional^16,55^ in conjunction with 6-31+G(d,p) basis set was used to obtain minima and transition states optimized geometries. The electrostatic interaction between the QM and MM region was described by the electronic embedding scheme^51,56^ implemented in ONIOM. The vibrational frequency was calculated to confirm that all transition states have only one negative frequency corresponding to the bond forming/breaking process and the minimum reaction energy path was determined by intrinsic reaction coordinate (IRC) calculation by ONIOM (M06-2X/6-31+G(d,p): AMBER) level. Single point calculation with electronic embedding scheme^57^ at ONIOM (M06-2X/6-31+G(d,p): AMBER) was performed on the obtained optimized structures (reactant, transition state and product). Two functionals- mPWB1W/6-311++G(2d,2p) and ωb97XD/6-311++G(2d,2p) were subsequently used for single point calculations to further evaluate the sensitivity of the density functional. These functionals have been reported to give accurate performances for both thermodynamics and kinetics when used together with the large 6-311++G(2d,2p) basis set^31,58^.

#### Natural bond orbital (NBO) calculations

Second-order perturbation analysis was performed on the transition states (TS) structures (TS1 and TS4). In NBO analysis, high E^2^ value demonstrates robust and strong interaction between acceptor and donors in a molecular system. The electronic wave functions are construed in terms of a set of unoccupied non-Lewis localised and molecular orbitals. The conjugative (delocalisation) interaction was determined using Eq. (1):

1 $$ E^{2 } = \Delta E_{ij} = q_{j} \frac{{F(i,j)^{2} }}{{\varepsilon_{j} - \varepsilon_{i} }} $$

where $q_{j}$ is the donor orbital occupancy, $\varepsilon_{i}$ and $\varepsilon_{j}$ are diagonal matrix elements and $F\left( {i,j} \right)$ is the off-diagonal Fock matrix element.

### Laboratory experimental details

#### Materials

DH5α and DreamTaq DNA polymerase from Thermo Fisher Scientific; designed primers (T7 promoter and T7 terminator) were synthesized by IDT DNA Technologies; Vent DNA polymerase from New England Biolabs; Ni-NTA agarose, plasmid extraction and Qiaprep Miniprep kit (Qiagen); *Escherichia coli* BL21, lactate dehydrogenase, nicotinamide adenine dinucleotide (NADH), MOPs, MgCl_2_, l-cysteine, ethylenediaminetetraacetic acid (EDTA), isocitrate from Sigma Aldrich; IPTG from OmniPur; lysozyme from Norgen Biotek; 2YT media (Novagen); skim milk, kanamycin and peroxidase stain DAB kit from Nacalai Tesque; goat anti-mouse HRP from Dako; nitrocellulose membrane from BioTrace; anti-6-His epitope tag from BioLegend.

#### Site directed mutagenesis of *M. tuberculosis* ICL

Wild type (WT) *M. tuberculosis* ICL gene was PCR-amplified with the designed primers (Supplementary Information Table S2) to produce Asp108Ala and Cys191Ser mutants. PCR reaction mix (20 μl) containing 2 μl Vent buffer, 1 μl each for forward and reverse primer, 2 μl dNTPs, 0.2 μl Vent DNA polymerase, 1 μl purified plasmid PM107 and 12.8 μl distilled water. PCR amplification condition was set up with 3 min (95 °C) initial denaturation, 30 s (95 °C) denaturation, 1 min (55 °C) annealing, 7 min (72 °C) elongation for 30 cycles and ended with 15 min (72 °C) as final extension. The PCR products were digested with 1 μl DpnI restriction enzyme to breakdown the original template. The digested PCR product was then transformed into DH5α via electroporation. The transformants were screened with colony PCR and the DNA sequence was checked to verify the desired mutants were acquired.

#### Expression of *M. tuberculosis* ICL

The purified plasmids of the verified clones were transformed into expression vector *E. coli* BL21. Cells containing recombinant constructs were inoculated into 5 ml 2YT media with kanamycin and grown overnight at 37 °C. The culture was diluted 1:100 with fresh 2YT media containing kanamycin and cultured at 37 °C, 200 rpm until OD_600nm_ reached 0.6 nm. Induction was initiated with 40 μl 1 M IPTG and incubation continued at 25 °C, 160 rpm overnight.

#### Extraction and purification of *M. tuberculosis* ICL

The overnight culture was centrifuged at 8000 rpm, 12 °C for 10 min. The bacterial pellet was re-suspended with 3 ml lysis buffer [50 mM NaH_2_PO_4_, 500 mM NaCl, 20 µg/ml lysozyme, pH 8]. The cell lysate was sonicated (Sonicator 3000, Misonix) with power of 3.5 for 3 min, followed by centrifugation at 8000 rpm, 12 °C for 30 min. The supernatant with ICL was purified with Ni-NTA agarose affinity chromatography column, with increasing imidazole concentration for elution.

#### SDS-PAGE and western blot analysis of *M. tuberculosis* ICL

Elution fractions containing expressed target protein were identified with SDS-PAGE and Western Blot. In SDS-PAGE analysis, the sample aliquots were mixed with 2× Laemmli sample buffer and electrophoresed in 10% SDS–polyacrylamide gel. Resulting protein bands were visualized with Coomasie Blue stain. For Western Blot, the protein from the electrophoresed gel was transferred to nitrocellulose membrane. The membrane was initially blocked with 2% skim milk for 1 h, followed by 1 h incubation with anti-6-His epitope tag and 1 h incubation with goat anti-mouse HRP. In between incubation steps were the washing steps with 1 × phosphate-buffered saline (PBS) with Tween 20 (PBS-T) 5 min for 3 times. The antibody tagged protein were visualized with peroxidase stain DAB kit.

#### *M. tuberculosis* ICL enzymatic assay

The reaction mixture was prepared with 58.3 μl purified ICL, 2.1 μl LDH, 46.7 μl NADH and MOPS buffer to a final volume of 700 μl. The mixture was transferred into a microtitre plate and pre-incubated at 37 °C, for 5 min followed by addition of 100 μl 20 mM isocitrate. The absorbance was immediately checked at 340 nm for 300 s at 10 s interval time. A negative control was set without the addition of purified ICL into the reaction mixture. Equations (2) and (3) are the calculations for ICL activity and specific activity, respectively. The calculated K_m_ and V_max_ are 25 mM and 0.97 µmol/min/mg, respectively.

2 $$ {\text{Enzyme }}\;{\text{activity}}\;({\text{U/ml}}) = \frac{{(absornace{/}min) (total \;volume) (DF)}}{(NADH\; extinction\; coefficient) (sample \;volume)(light \;path)} $$

3 $$ {\text{Specific}}\;{\text{activity}}\;(\upmu {\text{mol/min/mg}}) = \frac{Enzyme\;activity}{{ICL \;concentration}} $$

## Supplementary information

Supplementary Information.
