# Supplementary material for: Demystifying the catalytic pathway of Mycobacterium tuberculosis isocitrate lyase
Source: Sci Rep. 2020 Nov 3;10:18925. doi: 10.1038/s41598-020-75799-8 (PMC7609661; doi:10.1038/s41598-020-75799-8)
Supplement: Supplementary file 1 — Supplementary Information. [file 41598_2020_75799_MOESM1_ESM.docx]

**Demystifying the catalytic pathway of *Mycobacterium tuberculosis* isocitrate lyase**

Collins U. Ibeji^1,2,3^, Nor Amirah Mohd Salleh^1^, Jia Siang Sum^1^, [Angela Chiew Wen Ch'ng](https://www.sciencedirect.com/science/article/pii/S0076687919304252?via%3Dihub#!)^1^, Theam Soon Lim^1^, Yee Siew Choong^1,*^

^1^Institute for Research in Molecular Medicine (INFORMM), Universiti Sains Malaysia, 11800 Minden, Penang, Malaysia

^2^Catalysis and Peptide Research Unit, School of Health Sciences, University of KwaZulu-Natal, Durban 4041, South Africa.

^3^Department of Pure and Industrial Chemistry, Faculty of Physical Sciences, University of Nigeria, Nsukka 410001, Enugu State, Nigeria.

**Supplementary Information**

**Table S1.** Reaction mechanism (relative) entropy ΔS **(**cal/mol/K) obtained from ONIOM calculation using different density functionals at 6-311++G(2d,2p): Amber for the breakdown of isocitrate by *M. tuberculosis* isocitrate lyase (ICL).

**Table S2.** The forward and reverse primer design for *M. tuberculosis* isocitrate lyase (ICL) gene.

**Fig. S1. (A)** The starting structure for QM/MM study in this work and, **(B)** the structure obtained from x-ray crystal (PDB id: 1F8I).

**Fig. S2.** Backbone carbon (C_α_) atoms root mean square deviation (RMSD) of *M. tuberculosis* isocitrate lyase (ICL) with the function of time.

**Figure S3.** **(A)** View 1 and **(B)** View 2 of the optimized the geometries of the high layer (QM) of the ONIOM model in the study of *M. tuberculosis* isocitrate lyase (ICL) catalytic pathway.

**Table S1.** Reaction mechanism (relative) entropy ΔS **(**cal/mol/K) obtained from ONIOM calculation using different density functionals at 6-311++G(2d,2p): Amber for the breakdown of isocitrate by *M. tuberculosis* isocitrate lyase (ICL).

|  | B3LYP | M06-2X | ωB97X |
| --- | --- | --- | --- |
| Pathway **I** |  |  |  |
| R | 0.00 | 0.00 | 0.00 |
| PROD1 | -12.3 | -13.4 | -12.2 |
| Pathway **II** |  |  |  |
| PROD2 | -7.3 | -8.1 | -7.5 |

**Table S2.** The forward and reverse primer design for *M. tuberculosis* isocitrate lyase (ICL) gene.

|  | Forward primer | Reverse primer |
| --- | --- | --- |
| Wild type  (WT) | 5’-CGCGGATCCATGTCTGTCG-3, | 5’-TTTTCCTTTTGCGGCCGCACTAGTGGAACTGGCCCTCTTC-3’ |
| Asp108Ala | 5’-GATACAGGCTCTGGGCGGGGTAGGTGTGC-3’ | 5’-GCACACCTACCCCGCCCAGAGCCTGTATC-3’ |
| Cys191Ser | 5’-CCAGGTGGCCGCTCTTCTTCTTCTCAGAGG-3’ | 5’-CCTCTGAGAAGAAGAGCGGCCACCTGGG-3’ |


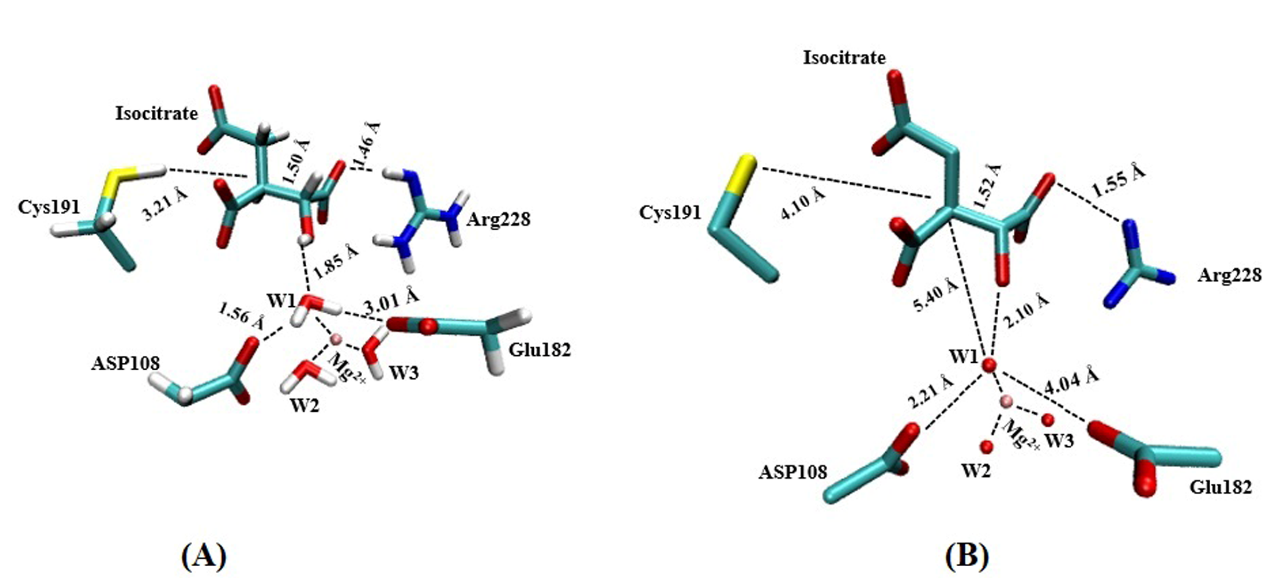


**Fig. S1. (A)** The starting structure for QM/MM study in this work and, **(B)** the structure obtained from x-ray crystal (PDB id: 1F8I).

**
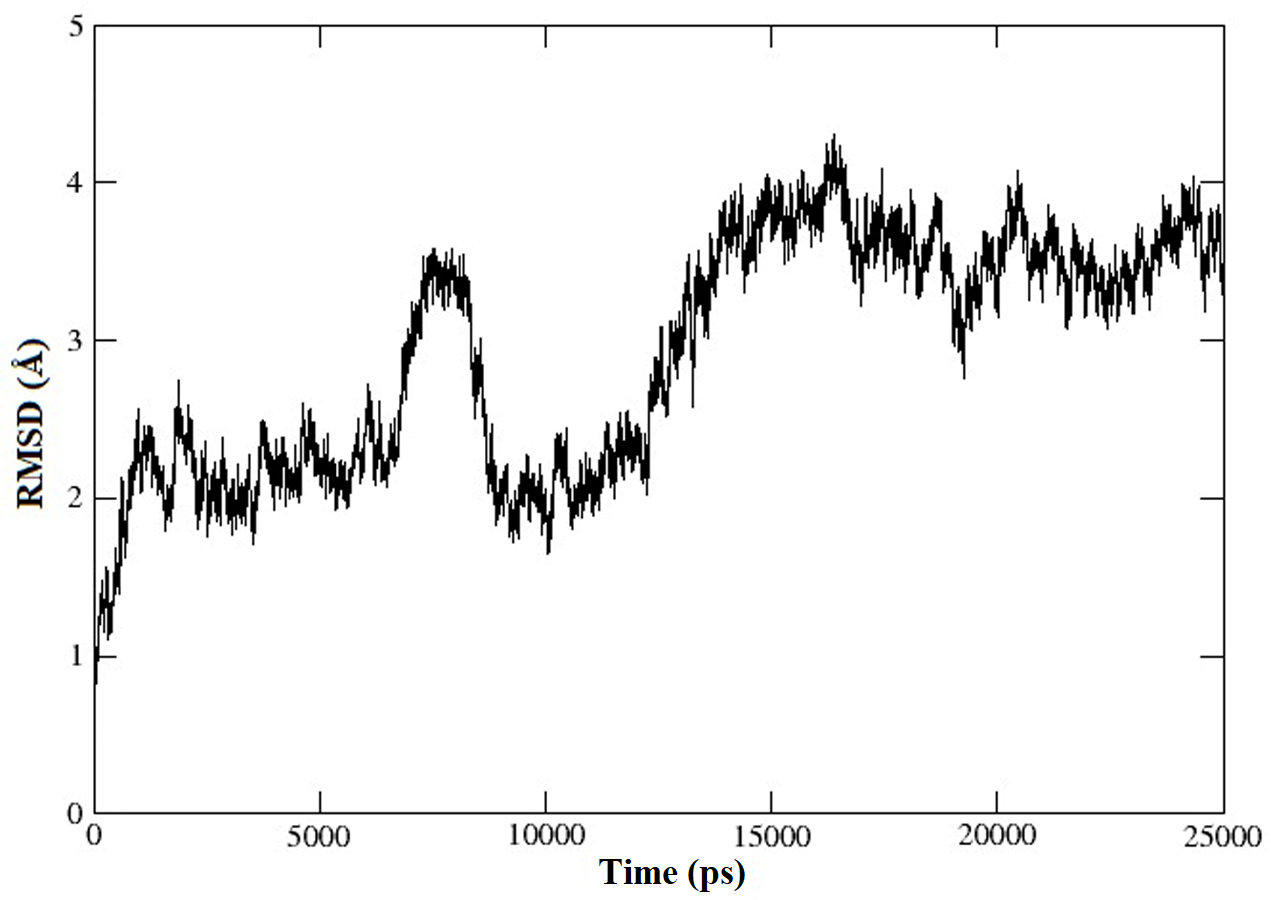
**

**Fig. S2.** Backbone carbon (C_α_) atoms root mean square deviation (RMSD) of *M. tuberculosis* isocitrate lyase (ICL) with the function of time.


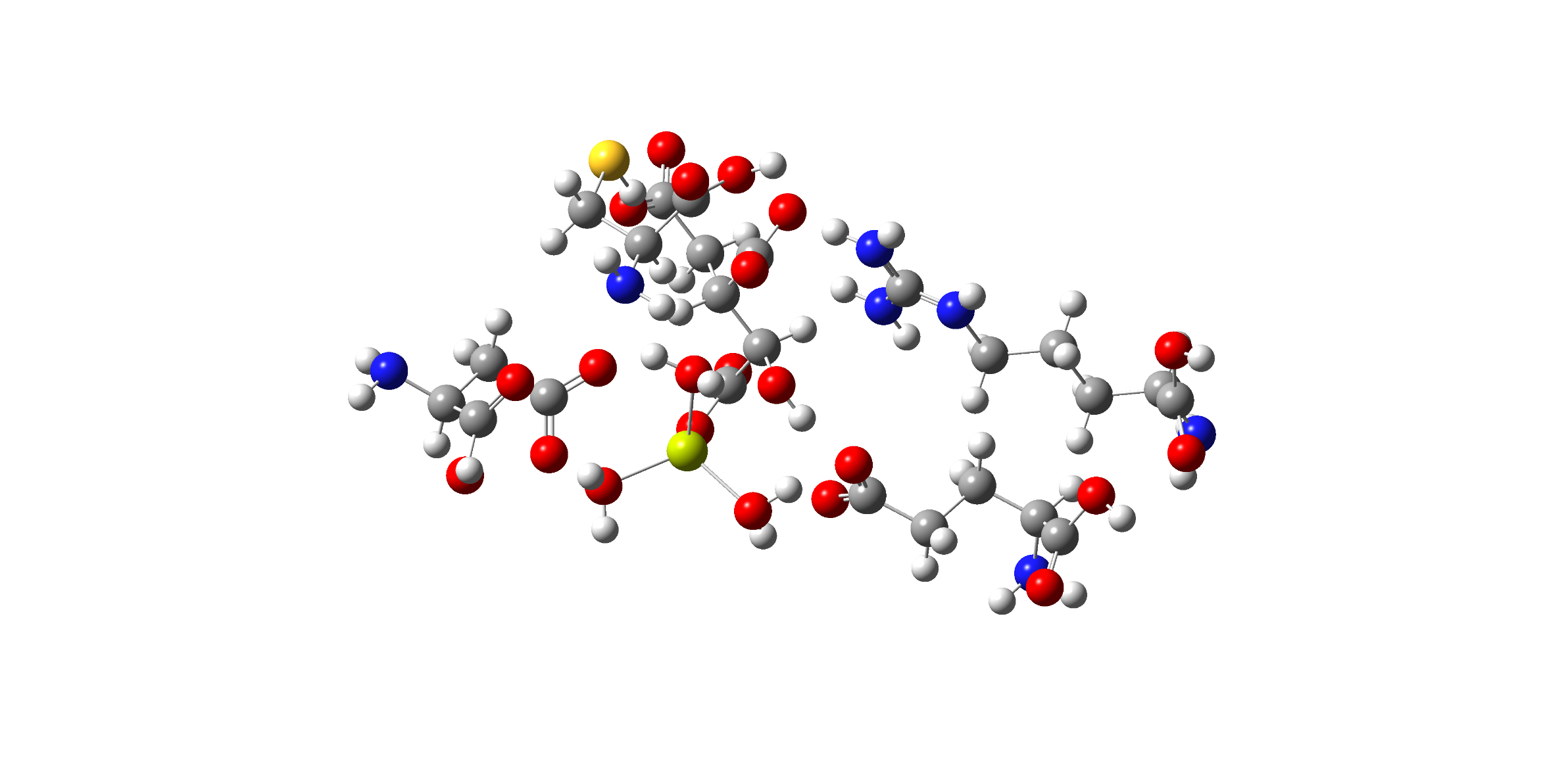


**(A)**


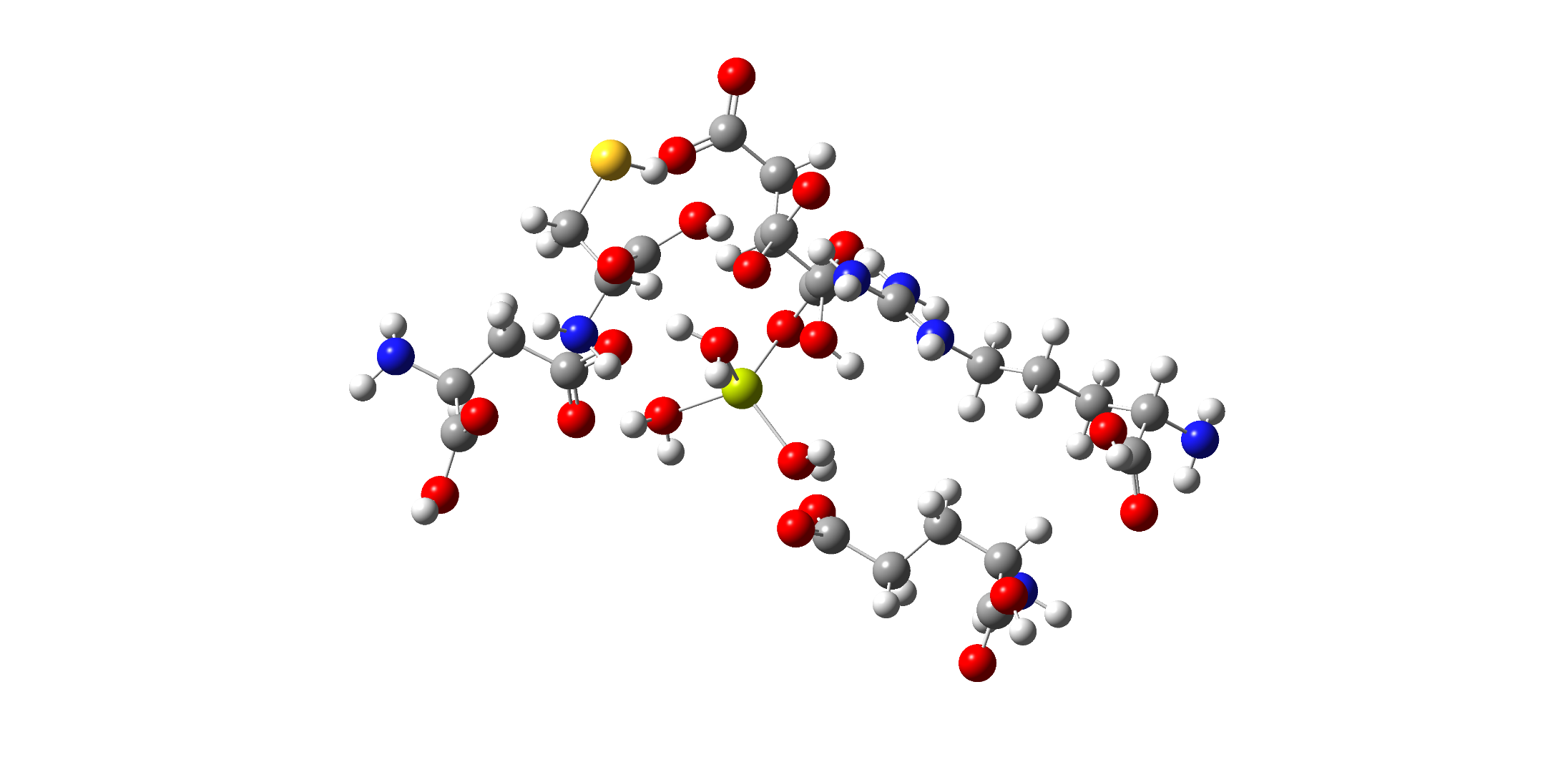


**(B)**

**Figure S3.** **(A)** View 1 and **(B)** View 2 of the optimized the geometries of the high layer (QM) of the ONIOM model in the study of *M. tuberculosis* isocitrate lyase (ICL) catalytic pathway.

The redundant co-ordinate of isocitrate lyase (QM/102 atoms)

N-N -1 30.78800000 41.12500000 43.75500000 H

H-H -1 30.92953700 40.49238092 44.51641997 H

C-CT -1 32.08600000 41.66000000 43.31400000 H

H-H1 -1 32.19500000 42.66600000 43.72400000 H

C-CT -1 33.21700000 40.81600000 43.91400000 H

H-HC -1 33.11300000 40.80100000 44.99900000 H

H-HC -1 33.13800000 39.79100000 43.54700000 H

C-C 0 34.58200000 41.40300000 43.55200000 H

O-O2 -1 34.80400000 42.60800000 43.79700000 H

O-O2 0 35.44200000 40.67800000 43.00900000 H

C-C -1 32.19500000 41.78100000 41.78000000 H

O-O 0 32.79551811 40.89371315 41.11994651 H

N-N 0 44.18600000 44.23000000 38.39600000 H

H-H 0 43.53289701 44.95290667 38.17047299 H

C-CT 0 43.87600000 43.02500000 37.60800000 H

H-H1 0 44.70400000 42.32300000 37.70200000 H

C-CT 0 42.63600000 42.33000000 38.19900000 H

H-HC 0 42.54500000 41.32700000 37.78100000 H

H-HC 0 42.76500000 42.24200000 39.27800000 H

C-CT 0 41.35800000 43.11900000 37.89100000 H

H-HC 0 41.55800000 44.17300000 38.03900000 H

H-HC 0 41.10000000 42.98000000 36.84000000 H

C-C 0 40.17500000 42.72500000 38.77400000 H

O-O2 0 40.20500000 42.94800000 40.00700000 H

O-O2 0 39.15800000 42.25400000 38.22900000 H

C-C 0 43.72300000 43.30400000 36.10400000 H

O-O 0 43.50986750 44.47660046 35.70007767 H

N-N 0 34.88900000 38.46900000 40.79400000 H

H-H -1 34.02039483 37.98555407 40.68534878 H

C-CT -1 35.73400000 37.74800000 41.75700000 H

H-H1 -1 36.48100000 38.43600000 42.14500000 H

C-CT -1 34.84900000 37.31100000 42.93900000 H

H-H1 -1 34.74400000 38.16000000 43.61300000 H

H-H1 -1 33.86100000 37.05500000 42.56800000 H

S-SH 0 35.47600000 35.87300000 43.86800000 H

H-HS 0 36.54100000 36.43500000 44.49200000 H

C-C -1 36.47800000 36.58300000 41.06800000 H

O-O 0 36.12063663 36.51099551 39.86355933 H

N-N 0 48.09900000 41.53600000 38.88000000 H

H-H -1 47.51435291 42.32826826 39.05463902 H

C-CT -1 47.28600000 40.31200000 38.90600000 H

H-H1 -1 47.87800000 39.50000000 39.32100000 H

C-CT -1 46.08700000 40.52400000 39.84400000 H

H-HC -1 46.45000000 40.54100000 40.87200000 H

H-HC -1 45.62900000 41.49100000 39.63200000 H

C-CT -1 45.00900000 39.43900000 39.70300000 H

H-HC -1 44.55900000 39.50300000 38.71100000 H

H-HC -1 45.44700000 38.44900000 39.83900000 H

C-CT -1 43.91300000 39.64900000 40.74100000 H

H-H1 -1 44.28800000 39.32400000 41.71100000 H

H-H1 -1 43.66600000 40.71000000 40.80300000 H

N-N2 -1 42.69700000 38.91300000 40.37100000 H

H-H -1 42.40400000 38.91100000 39.39300000 H

C-CA 0 41.79800000 38.43900000 41.20200000 H

N-N2 -1 41.98700000 38.38300000 42.48200000 H

H-H 0 42.89700000 38.53600000 42.86900000 H

H-H -1 41.25100000 37.93000000 43.02700000 H

N-N2 -1 40.66300000 38.00600000 40.76500000 H

H-H 0 40.54500000 37.83400000 39.76900000 H

H-H -1 39.94200000 37.82400000 41.46200000 H

C-C -1 46.87100000 39.88600000 37.49700000 H

O-O 0 46.47413712 40.74783032 36.67036894 H

Mg-MG 0 38.72200000 42.73700000 44.09200000 H

C-C -1 39.62900000 41.31400000 45.95600000 H

O-O -1 40.39000000 41.83000000 46.73800000 H

O-O -1 38.55900000 41.82100000 45.69700000 H

C-CT -1 40.14900000 40.16800000 45.09400000 H

O-OH -1 40.51200000 40.74700000 43.83600000 H

C-CT -1 39.16500000 38.94100000 44.98500000 H

C-CT -1 39.57000000 37.96000000 46.11900000 H

C-C -1 38.72400000 36.72400000 46.37200000 H

O-O2 -1 39.28700000 35.79200000 46.91400000 H

O-O2 -1 37.53700000 36.74700000 46.12600000 H

C-C -1 39.16600000 38.28400000 43.59600000 H

O-O2 -1 39.70000000 37.21400000 43.42100000 H

O-O2 -1 38.71000000 38.90400000 42.66100000 H

H-H1 -1 41.07500000 39.82900000 45.56700000 H

H-HC -1 38.14400000 39.27500000 45.18400000 H

H-HC -1 39.58800000 38.52800000 47.04700000 H

H-HC -1 40.58800000 37.63000000 45.91200000 H

H-HO -1 39.57777005 40.74005441 43.42665155 H

O-O 0 39.63800000 43.18800000 42.47200000 H

H-H -1 39.91200000 43.06500000 41.50400000 H

H-H -1 40.31200000 43.90300000 42.67900000 H

O-O -1 37.86900000 41.27900000 43.16900000 H

H-H 0 36.88500000 41.01700000 43.12800000 H

O-O -1 37.09500000 43.67900000 44.03500000 H

H-H -1 36.78400000 44.63000000 43.86800000 H

H-H -1 36.16000000 43.27800000 43.97400000 H

H-H -1 37.72719645 41.52074470 42.25082177 H

H-H 0 48.80602521 41.48158704 39.58509189 H

H-H 0 45.11256324 44.53915038 38.18174175 H

H-H 0 30.20065589 41.87789738 44.05193842 H

H-H 0 35.35631208 38.51727439 39.91122653 H

O-OH 0 46.93394573 38.50832728 37.11890515 H

H-HO 0 47.53125483 38.40738084 36.37416918 H

O-OH 0 43.82287431 42.23102730 35.16397090 H

H-HO 0 44.52460701 42.41905503 34.53642266 H

O-OH 0 31.61363541 42.90144679 41.10805193 H

H-HO 0 31.13427178 42.59741414 40.33385903 H

O-OH 0 36.16301103 35.35673062 41.73278970 H

H-HO 0 36.69160901 34.64627630 41.36206225 H

1 2 1.0 3 1.0 93 1.0

2

3 4 1.0 5 1.0 11 1.0

4

5 6 1.0 7 1.0 8 1.0

6

7

8 9 2.0 10 2.0

9

10

11 12 2.0 99 1.0

12

13 14 1.0 15 1.0 92 1.0

14

15 16 1.0 17 1.0 26 1.0

16

17 18 1.0 19 1.0 20 1.0

18

19

20 21 1.0 22 1.0 23 1.0

21

22

23 24 2.0 25 2.0

24

25

26 27 2.0 97 1.0

27

28 29 1.0 30 1.0 94 1.0

29

30 31 1.0 32 1.0 37 1.0

31

32 33 1.0 34 1.0 35 1.0

33

34

35 36 1.0

36

37 38 2.0 101 1.0

38

39 40 1.0 41 1.0 91 1.0

40

41 42 1.0 43 1.0 61 1.0

42

43 44 1.0 45 1.0 46 1.0

44

45

46 47 1.0 48 1.0 49 1.0

47

48

49 50 1.0 51 1.0 52 1.0

50

51

52 53 1.0 54 2.0

53

54 55 2.0 58 2.0

55 56 1.0 57 1.0

56

57

58 59 1.0 60 1.0

59

60

61 62 2.0 95 1.0

62

63 66 1.0 82 1.0 85 1.0 87 1.0

64 65 2.0 66 2.0 67 1.0

65

66

67 68 1.0 69 1.0 77 1.0

68

69 70 1.0 74 1.0 78 1.0

70 71 1.0 79 1.0 80 1.0

71 72 2.0 73 2.0

72

73

74 75 2.0 76 2.0

75

76

77

78

79

80

81

82 83 1.0 84 1.0

83

84

85 90 1.0

86

87 88 1.0

88

89

90

91

92

93

94

95 96 1.0

96

97 98 1.0

98

99 100 1.0

100

101 102 1.0

102

The redundant co-ordinate of isocitrate lyase complex (QM and MM layer)

N-N -1 -5.94906600 9.42568600 -10.12276000 L

H-H -1 -5.46117500 9.69761100 -9.28135700 L

C-CT -1 -7.05727900 8.48347500 -9.96810600 L

H-H1 -1 -7.80070200 8.66673400 -10.74677000 L

C-CT -1 -6.51155200 7.05855000 -10.14132000 L

H-HC -1 -5.76439700 6.84679500 -9.37526800 L

H-HC -1 -7.32707100 6.33958100 -10.06212400 L

H-HC -1 -6.05756100 6.95611900 -11.12698100 L

C-C -1 -7.74130700 8.64361600 -8.59760000 L

O-O -1 -7.15761000 9.18627500 -7.65264900 L

N-N -1 -8.97089900 8.13498600 -8.47809500 L

H-H -1 -9.37044400 7.65581300 -9.27521500 L

C-CT -1 -9.75335900 8.13199900 -7.23833800 L

H-H1 -1 -9.16880700 8.57271400 -6.42895900 L

C-CT -1 -11.03531300 8.96824200 -7.42405000 L

H-HC -1 -11.62939200 8.49891800 -8.20404000 L

H-HC -1 -11.60939300 8.92850800 -6.49787600 L

C-CT -1 -10.84141100 10.44231300 -7.82221000 L

H-HC -1 -10.30917500 10.49973000 -8.77011900 L

C-CT -1 -12.21130000 11.10125700 -7.99087800 L

H-HC -1 -12.75862600 11.08707800 -7.04895100 L

H-HC -1 -12.08525400 12.13527300 -8.31432300 L

H-HC -1 -12.78644000 10.57019200 -8.74953400 L

C-CT -1 -10.06708000 11.23997100 -6.77795900 L

H-HC -1 -9.05176800 10.85577300 -6.69839600 L

H-HC -1 -10.01069800 12.28376700 -7.08465600 L

H-HC -1 -10.56863700 11.17485300 -5.81348100 L

C-C -1 -10.08592700 6.69137300 -6.82620200 L

O-O -1 -10.44479600 5.86422900 -7.66451500 L

N-N -1 -9.99526000 6.38718000 -5.53397700 L

H-H -1 -9.63379800 7.08503500 -4.89152100 L

C-CT -1 -10.30955200 5.06620800 -4.99746700 L

H-H1 -1 -9.75981900 4.30870200 -5.54869000 L

H-H1 -1 -9.99259500 5.01664100 -3.96062200 L

C-C -1 -11.80149000 4.75341100 -5.05682400 L

O-O -1 -12.58493100 5.37653300 -4.33752100 L

N-N -1 -12.18643000 3.77751500 -5.87496200 L

H-H -1 -11.46641600 3.30707900 -6.41702900 L

C-CT -1 -13.54003900 3.23977900 -5.97149000 L

H-H1 -1 -14.25273600 3.99612800 -5.63961700 L

C-CT -1 -13.82646000 2.91961700 -7.44643700 L

H-HC -1 -13.09703400 2.19593900 -7.81522400 L

H-HC -1 -14.82290700 2.49472100 -7.55161000 L

H-HC -1 -13.76190100 3.82484900 -8.04753300 L

C-C -1 -13.72917700 1.98981300 -5.09143100 L

O-O -1 -12.77280900 1.26926500 -4.80496800 L

N-N -1 -14.98750100 1.69731500 -4.74223200 L

H-H -1 -15.69541800 2.39579600 -4.91203200 L

C-CT -1 -15.41178600 0.40796300 -4.16993600 L

H-H1 -1 -14.55204900 -0.26617100 -4.19146200 L

C-CT -1 -15.76233900 0.60460700 -2.68019300 L

H-HC -1 -15.70636500 -0.35940500 -2.17448100 L

H-HC -1 -14.97470000 1.22248700 -2.25110400 L

C-CT -1 -17.12855000 1.25505700 -2.37679200 L

H-HC -1 -17.45363900 1.84022600 -3.23504900 L

C-CT -1 -18.20064400 0.21471300 -2.04824500 L

H-HC -1 -17.92442100 -0.33286400 -1.14722000 L

H-HC -1 -19.16016400 0.70509200 -1.88482000 L

H-HC -1 -18.30585400 -0.49743200 -2.86339900 L

C-CT -1 -17.03168900 2.18701200 -1.16850400 L

H-HC -1 -16.33243300 2.99042300 -1.38941000 L

H-HC -1 -18.00666800 2.62198800 -0.95211300 L

H-HC -1 -16.67813100 1.63407300 -0.29788600 L

C-C -1 -16.49277200 -0.30757300 -5.00898300 L

O-O -1 -16.82999800 -1.45497000 -4.73680400 L

N-N -1 -16.19180500 7.89814500 -12.79716300 L

H-H -1 -16.32790600 6.96963200 -12.41361100 L

C-CT -1 -14.82182800 8.43280100 -12.86937200 L

H-H1 -1 -14.79715000 9.42042100 -12.40716900 L

C-CT -1 -13.83057900 7.51430700 -12.13264100 L

H-HC -1 -13.98954900 6.48238500 -12.43951300 L

H-HC -1 -12.81833700 7.78654000 -12.43063900 L

C-CT -1 -13.90189300 7.59740000 -10.60252100 L

H-HC -1 -13.66528100 8.61128100 -10.28423300 L

H-HC -1 -14.90856800 7.37260500 -10.26321100 L

C-C -1 -12.92032700 6.63975800 -9.92942600 L

O-O -1 -11.96229700 6.15196200 -10.50382000 L

N-N -1 -13.08980700 6.35749800 -8.65894300 L

H-H -1 -12.32059500 5.84057500 -8.24364300 L

H-H -1 -13.84342300 6.75248300 -8.13197400 L

C-C -1 -14.36781000 8.60118400 -14.32822600 L

O-O -1 -13.90005300 9.67665900 -14.70833500 L

N-N -1 -8.13526900 4.36807100 -12.81440800 L

H-H -1 -7.68074300 3.59258900 -13.28612300 L

C-CT -1 -9.00016200 4.01471000 -11.67690600 L

H-H1 -1 -9.49655700 4.90932000 -11.29470400 L

C-CT -1 -10.08901700 3.01925100 -12.14835200 L

H-HC -1 -9.57377100 2.16199200 -12.58431000 L

C-CT -1 -10.95896900 2.50579400 -10.98173900 L

H-HC -1 -11.41110200 3.34896500 -10.45523100 L

H-HC -1 -11.75120500 1.85846500 -11.35112400 L

H-HC -1 -10.36162000 1.92220800 -10.28132200 L

C-CT -1 -11.01190900 3.63235900 -13.23083700 L

H-HC -1 -11.81223100 4.20351300 -12.75670700 L

H-HC -1 -10.45962200 4.31629400 -13.87278200 L

C-CT -1 -11.61522000 2.55811200 -14.14180000 L

H-HC -1 -12.14968900 1.81213400 -13.55896600 L

H-HC -1 -12.30825200 3.02312000 -14.84208000 L

H-HC -1 -10.82095900 2.06424300 -14.70186800 L

C-C -1 -8.13445700 3.42188800 -10.55394800 L

O-O -1 -7.14529800 2.73654400 -10.82272800 L

N-N 0 -8.49795200 3.69744300 -9.29621200 L

H-H 0 -9.30966900 4.27633100 -9.13435800 L

C-CT 0 -7.82927700 3.14759800 -8.12375800 L

H-H1 0 -7.01480300 2.49942700 -8.44659700 L

C-CT 0 -7.19713100 4.28461100 -7.30654000 L

H-HC 0 -6.62990600 4.92275900 -7.98489700 L

H-HC 0 -7.98240500 4.89817000 -6.87417900 L

C-CA 0 -6.25578100 3.83301700 -6.20476900 L

C-CA 0 -4.90336700 3.57577500 -6.50209100 L

H-HA 0 -4.54084300 3.68904300 -7.51218900 L

C-CA 0 -4.01280500 3.18982100 -5.48583600 L

H-HA 0 -2.97257800 3.01388800 -5.71920800 L

C-C 0 -4.47950300 3.05561800 -4.15735100 L

O-OH 0 -3.61545900 2.73608300 -3.15210800 L

H-HO 0 -2.71352700 2.61393400 -3.45739400 L

C-CA 0 -5.84318100 3.28185000 -3.86551400 L

H-HA 0 -6.20298900 3.17524400 -2.85365000 L

C-CA 0 -6.72242300 3.67990300 -4.88437900 L

H-HA 0 -7.75595000 3.86688400 -4.64011700 L

C-C 0 -8.81568600 2.28669100 -7.32879000 L

O-O 0 -10.03241800 2.42321300 -7.45500700 L

N-N 0 -8.26065200 1.37446600 -6.52714300 L

H-H 0 -7.25227200 1.32430500 -6.49712900 L

C-CT 0 -8.96712400 0.45200000 -5.65707100 L

H-H1 0 -9.91822900 0.90076600 -5.36603000 L

C-CT 0 -9.24452600 -0.86589700 -6.40470300 L

H-HC 0 -9.93217600 -0.65596500 -7.22598900 L

H-HC 0 -8.31052800 -1.22355300 -6.83804200 L

C-CT 0 -9.83362400 -1.99699200 -5.53412400 L

H-HC 0 -9.11347900 -2.26050800 -4.75811600 L

C-CT 0 -11.15791900 -1.61302300 -4.85701100 L

H-HC 0 -11.89528100 -1.33154600 -5.60938800 L

H-HC 0 -11.52947400 -2.45936100 -4.27943600 L

H-HC 0 -11.01234500 -0.78098800 -4.17123800 L

C-CT 0 -10.05763400 -3.23890700 -6.40769800 L

H-HC 0 -9.11498100 -3.54098900 -6.85987900 L

H-HC 0 -10.42855200 -4.06117800 -5.79677400 L

H-HC 0 -10.77903200 -3.02387000 -7.19702400 L

C-C 0 -8.10495300 0.25494500 -4.41218400 L

O-O 0 -6.90336100 -0.00229300 -4.50272000 L

N-N 0 -8.75273500 0.39048100 -3.25241400 L

H-H 0 -9.74472000 0.57806300 -3.28227400 L

C-CT 0 -8.13808900 0.43236000 -1.94128400 L

H-H1 0 -7.05618600 0.35303900 -2.03878100 L

C-CT 0 -8.48986500 1.78613300 -1.31814600 L

H-H1 0 -8.14099700 2.58503600 -1.96978800 L

H-H1 0 -9.56892300 1.85777200 -1.22344300 L

O-OH 0 -7.91886400 1.95316300 -0.04436500 L

H-HO 0 -6.96408500 1.90028800 -0.12923100 L

C-C 0 -8.66859100 -0.73268900 -1.11054500 L

O-O 0 -9.88042500 -0.90420800 -0.99160700 L

N-N 0 -7.75220700 -1.51303300 -0.52265100 L

H-H 0 -6.77359000 -1.30993000 -0.66909700 L

C-CT 0 -8.06827300 -2.61178000 0.38363600 L

H-H1 0 -8.77567900 -3.27981900 -0.10589500 L

H-H1 0 -7.15976300 -3.17239800 0.58487500 L

C-C 0 -8.65409400 -2.13624300 1.71815300 L

O-O 0 -9.28939700 -2.92186300 2.41883000 L

N-N 0 -8.45520600 -0.85259300 2.05029300 L

H-H 0 -7.89523800 -0.28654900 1.42874300 L

C-CT 0 -9.12749600 -0.13485600 3.12142000 L

H-H1 0 -8.90917400 -0.63415400 4.06523600 L

C-CT 0 -8.56882300 1.29427300 3.16603200 L

H-HC 0 -7.48783900 1.24206400 3.02917400 L

H-HC 0 -8.97121100 1.84878200 2.32203300 L

C-C* 0 -8.81615100 2.09759200 4.40688700 L

C-CW 0 -7.84007200 2.52663900 5.23400200 L

H-H4 0 -6.79010700 2.32814900 5.08938300 L

N-NA 0 -8.38145800 3.24871700 6.27325900 L

H-H 0 -7.81957500 3.64981000 7.01050700 L

C-CN 0 -9.74898800 3.36346300 6.14579900 L

C-CA 0 -10.73006700 4.00878300 6.91558200 L

H-HA 0 -10.45855400 4.53289100 7.82015700 L

C-CA 0 -12.06815600 3.96795300 6.49250500 L

H-HA 0 -12.83706300 4.45479200 7.07426900 L

C-CA 0 -12.40130000 3.30086200 5.30164300 L

H-HA 0 -13.42562900 3.26704000 4.96894800 L

C-CA 0 -11.40857500 2.67183600 4.53153400 L

H-HA 0 -11.69770700 2.19817800 3.60812100 L

C-CB 0 -10.05737700 2.65754800 4.94328100 L

C-C 0 -10.64072800 -0.13975300 2.87617900 L

O-O 0 -11.40051600 -0.46783100 3.78350700 L

N-N 0 -11.06364100 0.23298600 1.65645900 L

H-H 0 -10.36833600 0.48834600 0.96964400 L

C-CT 0 -12.45585700 0.30589600 1.22103500 L

H-H1 0 -13.02876100 0.81493000 1.99763800 L

C-CT 0 -12.56687300 1.13384700 -0.06914800 L

H-HC 0 -12.02399200 0.62788600 -0.86740100 L

H-HC 0 -13.61837500 1.18206400 -0.35176100 L

C-CT 0 -12.04588900 2.57419900 0.07401900 L

H-HC 0 -12.58175800 3.06631900 0.88605300 L

H-HC 0 -10.98744500 2.56759400 0.33270800 L

C-C 0 -12.23397000 3.37848600 -1.21510400 L

O-O 0 -12.89634500 4.41133600 -1.21133400 L

N-N 0 -11.65094700 2.91705500 -2.32460600 L

H-H 0 -11.75206000 3.41952400 -3.19476800 L

H-H 0 -11.11082200 2.06410300 -2.29368900 L

C-C 0 -13.08053400 -1.08198200 1.00113200 L

O-O 0 -14.30146700 -1.19874800 1.09332000 L

N-N 0 -12.27234700 -2.11866200 0.71222300 L

H-H 0 -11.27955900 -1.95217800 0.62823300 L

C-CT 0 -12.71386100 -3.51288900 0.65038400 L

H-H1 0 -13.59200200 -3.56327200 0.00933300 L

C-CT 0 -11.62572100 -4.41989900 0.03013400 L

H-HC 0 -10.68999100 -4.27871400 0.56883000 L

C-CT 0 -11.99939500 -5.90985400 0.10952200 L

H-HC 0 -12.95197600 -6.08269100 -0.38987500 L

H-HC 0 -11.22967100 -6.50935300 -0.37710400 L

H-HC 0 -12.07362300 -6.23006800 1.14848300 L

C-CT 0 -11.41093800 -4.06964400 -1.45326500 L

H-HC 0 -11.12213900 -3.02797200 -1.56344500 L

H-HC 0 -10.62051200 -4.68988200 -1.87405900 L

H-HC 0 -12.33066400 -4.23698100 -2.01337700 L

C-C 0 -13.13978900 -3.95832300 2.05327900 L

O-O 0 -14.26832000 -4.41462500 2.21926500 L

N-N 0 -12.26639000 -3.78616000 3.05805200 L

H-H 0 -11.35452200 -3.40560900 2.84934800 L

C-CT 0 -12.58587200 -3.99944000 4.46655600 L

H-H1 0 -12.78845900 -5.06239900 4.60626300 L

C-CT 0 -11.37056800 -3.63322700 5.32858600 L

H-HC 0 -11.18568600 -2.56104100 5.29298600 L

H-HC 0 -11.54987400 -3.92590300 6.36131400 L

H-HC 0 -10.48538100 -4.15490900 4.96729900 L

C-C 0 -13.83808000 -3.20384600 4.87581700 L

O-O 0 -14.71006800 -3.75025100 5.54846000 L

N-N 0 -13.93007000 -1.93905200 4.43104800 L

H-H 0 -13.17176500 -1.59072100 3.86238700 L

C-CT 0 -15.00693800 -0.99221700 4.68830800 L

H-H1 0 -15.04086300 -0.78914300 5.75934400 L

H-H1 0 -14.77548200 -0.05945600 4.17338300 L

C-C 0 -16.38474500 -1.47603200 4.22529800 L

O-O 0 -17.34230600 -1.35282300 4.98842700 L

N-N -1 -16.49735400 -2.00565100 2.99411600 L

H-H -1 -15.64855900 -2.12841800 2.45274300 L

C-CT -1 -17.75346000 -2.62274000 2.51812500 L

H-H1 -1 -18.22942500 -3.11050300 3.36764100 L

C-CT -1 -18.70295500 -1.53708900 1.95921100 L

H-HC -1 -18.54879300 -0.59908500 2.49477700 L

H-HC -1 -18.47973500 -1.35272300 0.90765800 L

C-C -1 -20.17444700 -1.92825600 2.12975400 L

O-O2 -1 -20.57470400 -2.08454500 3.31181600 L

O-O2 -1 -20.93522600 -2.00653700 1.13462700 L

C-C -1 -17.59018500 -3.71613200 1.45386000 L

O-O -1 -18.34323900 -4.68633100 1.46328900 L

N-N -1 -16.63945500 -3.57414700 0.52692500 L

H-H -1 -16.00630800 -2.78900500 0.60008700 L

C-CT -1 -16.61073000 -4.37089800 -0.70529700 L

H-H1 -1 -17.64975800 -4.56577400 -0.97900000 L

C-CT -1 -16.02670000 -3.50257100 -1.82970100 L

H-HC -1 -14.96902900 -3.30737200 -1.65705900 L

H-HC -1 -16.14635300 -4.01323700 -2.78338900 L

H-HC -1 -16.56691200 -2.55856900 -1.88327500 L

C-C -1 -15.96536000 -5.77359000 -0.56924100 L

O-O -1 -15.48731000 -6.33585600 -1.55545400 L

N-N -1 -15.92978500 -6.35014900 0.63533300 L

H-H -1 -16.39909500 -5.87598600 1.39693500 L

C-CT -1 -15.40045200 -7.69339500 0.87595700 L

H-H1 -1 -14.56070700 -7.82972700 0.19898200 L

C-CT -1 -14.83145300 -7.80359500 2.31057700 L

H-HC -1 -14.19596700 -8.68629100 2.34811300 L

H-HC -1 -14.20795100 -6.94026700 2.53208800 L

C-C -1 -15.87506000 -7.94660700 3.40960600 L

O-O -1 -17.07013300 -7.83237700 3.21180200 L

N-N -1 -15.46764700 -8.29061000 4.60382600 L

H-H -1 -16.19787900 -8.55629700 5.25119700 L

H-H -1 -14.49614400 -8.31250500 4.83843500 L

C-C -1 -16.41284600 -8.80801600 0.53708500 L

O-O -1 -17.61026400 -8.58200200 0.35846100 L

N-N -1 -15.94449400 -10.05835500 0.52762300 L

H-H -1 -14.94068500 -10.18299500 0.58616000 L

C-CT -1 -16.78507300 -11.25889900 0.38775600 L

H-H1 -1 -17.56449300 -11.02406500 -0.33770700 L

C-CT -1 -15.93061900 -12.40842600 -0.19498500 L

H-HC -1 -15.05966100 -12.55233100 0.44791300 L

H-HC -1 -16.51079600 -13.33093800 -0.18503500 L

C-CT -1 -15.46491100 -12.16428900 -1.64480000 L

H-HC -1 -15.00321700 -11.18470100 -1.71966800 L

C-CT -1 -14.42698900 -13.21172000 -2.04330000 L

H-HC -1 -14.86505600 -14.20837900 -1.99597100 L

H-HC -1 -14.07105800 -13.01737600 -3.05559500 L

H-HC -1 -13.57942200 -13.15680500 -1.36034700 L

C-CT -1 -16.61331300 -12.22584900 -2.65559500 L

H-HC -1 -17.33218700 -11.43174600 -2.45889700 L

H-HC -1 -16.22162400 -12.08727200 -3.66284600 L

H-HC -1 -17.11140100 -13.19203900 -2.59251500 L

C-C -1 -17.57016000 -11.63229500 1.67296500 L

O-O -1 -17.75212800 -12.81092200 1.97722000 L

N-N -1 -19.56551100 -6.79078000 5.41048700 L

H-H -1 -18.75833500 -7.20494400 4.95951600 L

C-CT -1 -19.40195100 -6.25408000 6.77418600 L

H-H1 -1 -20.38095400 -6.02553600 7.19993000 L

C-CT -1 -18.75377200 -7.33359300 7.66239800 L

H-HC -1 -17.75434000 -7.54284800 7.27991600 L

H-HC -1 -18.65063500 -6.94904900 8.67676700 L

C-CC -1 -19.51116400 -8.63970800 7.76364200 L

N-NB -1 -18.93648600 -9.86105700 8.12905000 L

C-CR -1 -19.93295600 -10.76502300 8.10152400 L

H-H5 -1 -19.82336200 -11.81791900 8.33598300 L

N-NA -1 -21.07801100 -10.18251000 7.71340400 L

H-H -1 -21.95906600 -10.66276500 7.55323000 L

C-CW -1 -20.83765000 -8.84271900 7.50901700 L

H-H4 -1 -21.55662300 -8.09800700 7.19438100 L

C-C -1 -18.63065700 -4.91917400 6.76107100 L

O-O -1 -18.52623600 -4.27374400 5.71706700 L

N-N -1 -18.12787400 -4.45843600 7.91100800 L

H-H -1 -18.28677000 -4.98700500 8.75651800 L

C-CT -1 -17.32324400 -3.22709300 8.02432300 L

H-H1 -1 -16.87685100 -2.97946000 7.06001800 L

C-CT -1 -18.19644000 -2.04228100 8.48072400 L

H-H1 -1 -18.57781600 -2.24150600 9.48263800 L

C-CT -1 -17.42156400 -0.72427900 8.48450400 L

H-HC -1 -16.96828800 -0.55045600 7.50845800 L

H-HC -1 -18.09723300 0.09855300 8.71534700 L

H-HC -1 -16.64056000 -0.74948400 9.24441200 L

O-OH -1 -19.29983400 -1.84630900 7.61673300 L

H-HO -1 -20.00997800 -1.50212500 8.20014400 L

C-C -1 -16.19538000 -3.45198500 9.03566900 L

O-O -1 -16.44714600 -3.51322200 10.24015400 L

N-N 0 -14.95988700 -3.55021300 8.53340900 L

H-H 0 -14.84099500 -3.46324000 7.53498100 L

C-CT 0 -13.75764900 -3.84064200 9.30279000 L

H-H1 0 -14.02146200 -3.93056100 10.35571100 L

C-CT 0 -13.15226600 -5.17273600 8.83403200 L

H-HC 0 -12.95513700 -5.11423500 7.76349100 L

H-HC 0 -12.19175800 -5.31036500 9.33063200 L

C-CA 0 -13.99402100 -6.40111300 9.11686200 L

C-CA 0 -14.18792200 -6.82452000 10.44575700 L

H-HA 0 -13.75776400 -6.26396300 11.26315500 L

C-CA 0 -14.93542600 -7.98283400 10.71817900 L

H-HA 0 -15.07952300 -8.30085400 11.74060500 L

C-C 0 -15.48624500 -8.73347200 9.65322900 L

O-OH 0 -16.20189000 -9.86561000 9.91309500 L

H-HO 0 -16.27165200 -10.05827300 10.85097300 L

C-CA 0 -15.28622900 -8.30978500 8.31847900 L

H-HA 0 -15.69859400 -8.88175200 7.50014700 L

C-CA 0 -14.54942600 -7.14213900 8.05521600 L

H-HA 0 -14.40069500 -6.82736100 7.03240600 L

C-C 0 -12.73164500 -2.71460400 9.12582400 L

O-O 0 -12.68816100 -2.09402800 8.06055000 L

N-N 0 -11.86658400 -2.47421400 10.13199300 L

C-CT 0 -11.88394300 -3.06969900 11.46324600 L

H-H1 0 -11.94480200 -4.15685300 11.41492100 L

H-H1 0 -12.73117100 -2.67047900 12.02181000 L

C-CT 0 -10.57784400 -2.64899100 12.13894100 L

H-HC 0 -9.80689900 -3.39685200 11.95644600 L

H-HC 0 -10.70954900 -2.49098400 13.20968800 L

C-CT 0 -10.21031200 -1.35600500 11.41654400 L

H-HC 0 -9.13764800 -1.16141000 11.45166900 L

H-HC 0 -10.76020400 -0.52463000 11.86029000 L

C-CT 0 -10.71641100 -1.59554600 9.99110200 L

H-H1 0 -11.02203700 -0.64342700 9.55418300 L

C-C 0 -9.65217200 -2.24858800 9.09842900 L H-H 355 0.0000

O-O 0 -9.64106500 -3.46442200 8.89482900 L

N-N -1 -8.77239700 -1.40967600 8.56895800 H

H-H -1 -8.90284000 -0.42328700 8.72423800 H

C-CT -1 -7.72351900 -1.79295200 7.61058800 H

H-H1 -1 -8.14713100 -2.52354400 6.91861800 H

C-CT -1 -7.32672700 -0.57200600 6.77196700 H

H-HC -1 -8.21578500 -0.16935600 6.28642500 H

H-HC -1 -6.91386400 0.19816800 7.42613300 H

C-C 0 -6.40359200 -0.91469700 5.66009800 H

O-O2 -1 -6.56854700 -1.90801300 4.93247000 H

O-O2 -1 -5.43069000 -0.03438900 5.45375200 H

C-C -1 -6.50799500 -2.46011400 8.28666900 H

O-O 0 -5.51071100 -1.74917000 8.42197000 H

N-N 0 -6.65603100 -3.75130900 8.65965400 L H-H1 365 0.0000

H-H 0 -7.53079600 -4.22121900 8.47520700 L

C-CT 0 -5.64273200 -4.49211900 9.40356200 L

H-H1 0 -4.67313700 -4.04121500 9.20432400 L

C-CT 0 -5.93675500 -4.39522400 10.91757500 L

H-HC 0 -6.87913000 -4.90021700 11.13339900 L

H-HC 0 -5.14115300 -4.91359100 11.45482000 L

C-CT 0 -6.01585300 -2.95906000 11.45968100 L

H-HC 0 -5.12841300 -2.40743500 11.15185100 L

H-HC 0 -6.89149700 -2.45680300 11.04636400 L

C-C 0 -6.10244800 -2.94315200 12.98494000 L

O-O 0 -5.09111200 -3.09551000 13.66701500 L

N-N 0 -7.31026400 -2.75767600 13.52506000 L

H-H 0 -7.41709000 -2.73930100 14.52917200 L

H-H 0 -8.11639100 -2.63581700 12.92869200 L

C-C 0 -5.57195500 -5.95390200 8.92855200 L

O-O 0 -5.36266300 -6.86042800 9.73658900 L

N-N 0 -5.72521800 -6.17268600 7.61127600 L

H-H 0 -5.85497200 -5.36842700 7.01423200 L

C-CT 0 -5.87327600 -7.47347400 6.96162400 L

H-H1 0 -6.18989000 -7.28396200 5.93693100 L

C-CT 0 -4.52498400 -8.21745200 6.89827300 L

H-H1 0 -4.18150200 -8.44346900 7.90784800 L

H-H1 0 -4.66146600 -9.15449100 6.35791300 L

O-OH 0 -3.53126600 -7.45862700 6.24189700 L

H-HO 0 -3.82861900 -7.27592200 5.34758200 L

C-C 0 -6.98602800 -8.29384900 7.62990800 L

O-O 0 -6.70986600 -9.25557600 8.34819800 L

N-N 0 -8.24438100 -7.89365900 7.38473300 L

H-H 0 -8.38489100 -7.10119800 6.77455300 L

C-CT 0 -9.44705800 -8.46932700 7.98943700 L

H-H1 0 -9.20623000 -9.43581300 8.43066000 L

C-CT 0 -9.97236200 -7.53659300 9.10435000 L

H-HC 0 -10.17388600 -6.56120600 8.65766900 L

H-HC 0 -10.91759400 -7.93348700 9.47871000 L

C-CT 0 -9.03496700 -7.34151900 10.31433700 L

H-HC 0 -8.07302000 -6.96815000 9.96805600 L

C-CT 0 -9.63845300 -6.28724900 11.25224300 L

H-HC 0 -10.60470100 -6.62440500 11.63013600 L

H-HC 0 -8.96690400 -6.10663900 12.09229300 L

H-HC 0 -9.77594300 -5.35497700 10.70903600 L

C-CT 0 -8.81003800 -8.64041600 11.10534700 L

H-HC 0 -8.31983500 -9.38702000 10.48322000 L

H-HC 0 -8.17074400 -8.44101300 11.96610400 L

H-HC 0 -9.76423800 -9.03763100 11.45382600 L

C-C 0 -10.50923900 -8.72966500 6.90784600 L

O-O 0 -11.68683800 -8.41415000 7.08961500 L

N-N 0 -10.08813500 -9.33607700 5.78662900 L

H-H 0 -9.10747600 -9.56022000 5.69640500 L

C-CT 0 -10.96533000 -9.79058100 4.71306200 L

H-H1 0 -11.85532800 -10.20171000 5.18399700 L

C-CT 0 -11.40235200 -8.60609900 3.83298500 L

H-HC 0 -12.04559700 -8.99238800 3.04342000 L

H-HC 0 -12.00968000 -7.92436700 4.42978100 L

C-CA 0 -10.27796200 -7.81552900 3.19033500 L

C-CA 0 -9.83761400 -8.15081300 1.89696200 L

H-HA 0 -10.29402200 -8.96775600 1.36130100 L

C-CA 0 -8.80225000 -7.42313600 1.29223300 L

H-HA 0 -8.46534000 -7.69562000 0.30581800 L

C-C 0 -8.20685200 -6.33945100 1.97583800 L

O-OH 0 -7.21506500 -5.62359600 1.37560600 L

H-HO 0 -6.88087500 -4.91137200 1.92574700 L

C-CA 0 -8.64378400 -6.00485300 3.27889000 L

H-HA 0 -8.19027400 -5.18263400 3.81313400 L

C-CA 0 -9.67670800 -6.74440500 3.88000400 L

H-HA 0 -10.00671800 -6.48673400 4.87561100 L

C-C 0 -10.26904500 -10.87479700 3.86267600 L

O-O 0 -9.05138000 -10.80334900 3.68084200 L

N-N -1 -11.02064300 -11.85181900 3.30617900 L

C-CT -1 -12.43850800 -12.07479200 3.56000900 L

H-H1 -1 -13.03140200 -11.38630400 2.95532500 L

H-H1 -1 -12.69783400 -11.97253500 4.61336600 L

C-CT -1 -12.69951400 -13.51022500 3.11631400 L

H-HC -1 -13.74247900 -13.66637100 2.84194900 L

H-HC -1 -12.39589900 -14.20232000 3.90398100 L

C-CT -1 -11.76695600 -13.64848200 1.91684900 L

H-HC -1 -12.27232100 -13.23549500 1.04197200 L

H-HC -1 -11.49250500 -14.68868200 1.73850400 L

C-CT -1 -10.54428200 -12.79484900 2.29188100 L

H-H1 -1 -9.78649300 -13.43751500 2.74170800 L

C-C -1 -9.94740400 -12.06085700 1.08065000 L

O-O -1 -10.66305100 -11.33677100 0.38845200 L

N-N -1 -8.65249300 -12.24624800 0.80251700 L

H-H -1 -8.10179100 -12.77078300 1.46465400 L

C-CT -1 -7.91667300 -11.48456600 -0.21916000 L

H-H1 -1 -7.98125300 -10.42368000 0.03316400 L

C-CT -1 -6.44314900 -11.89517400 -0.14385900 L

H-HC -1 -6.32280400 -12.94202400 -0.42320500 L

H-HC -1 -5.85943900 -11.27273400 -0.82428600 L

H-HC -1 -6.06760100 -11.74411000 0.86869400 L

C-C -1 -8.46174900 -11.64859300 -1.65563500 L

O-O -1 -8.36344500 -10.73042200 -2.46770200 L

N-N -1 -9.12962000 -12.76975400 -1.95465800 L

H-H -1 -9.26882300 -13.44221600 -1.21279100 L

C-CT -1 -9.88429000 -12.98973500 -3.19775200 L

H-H1 -1 -9.20112100 -12.80995000 -4.02906300 L

C-CT -1 -10.33839600 -14.46652200 -3.29015900 L

H-HC -1 -10.88455600 -14.60284900 -4.22349400 L

H-HC -1 -9.45979500 -15.10934200 -3.32602400 L

C-C -1 -11.22034000 -14.94796400 -2.14591100 L

O-O -1 -11.23209400 -14.39365000 -1.05959900 L

N-N -1 -11.94967500 -16.02461000 -2.31798600 L

H-H -1 -12.49841300 -16.32928700 -1.53561600 L

H-H -1 -11.93135700 -16.56749200 -3.17334800 L

C-C -1 -11.05395700 -11.99550600 -3.40025100 L

O-O -1 -11.58615600 -11.89991300 -4.50663900 L

N-N -1 -11.39460200 -11.19413400 -2.38410500 L

H-H -1 -10.95351900 -11.35383700 -1.48674400 L

C-CT -1 -12.28349700 -10.03410300 -2.51670400 L

H-H1 -1 -13.25292000 -10.36493300 -2.88650800 L

C-CT -1 -12.48556600 -9.32271700 -1.17249300 L

H-H1 -1 -11.53265700 -8.91985900 -0.82585000 L

H-H1 -1 -13.17774000 -8.49338400 -1.32420900 L

O-OH -1 -13.00799200 -10.18831500 -0.17929300 L

H-HO -1 -12.25401800 -10.74319000 0.10324200 L

C-C -1 -11.73268800 -9.00229700 -3.50754100 L

O-O -1 -12.49576800 -8.50448400 -4.32813000 L

N-N -1 -10.42772800 -8.69094200 -3.49012400 L

H-H -1 -9.79868700 -9.19377000 -2.87271600 L

C-CT -1 -9.87363400 -7.61206700 -4.33570800 L

H-H1 -1 -10.52045700 -6.74692500 -4.18478300 L

C-CT -1 -8.45319600 -7.15716600 -3.91798400 L

H-HC -1 -7.70998700 -7.85519900 -4.28830200 L

C-CT -1 -8.15332400 -5.77807600 -4.51545900 L

H-HC -1 -8.83400500 -5.03245700 -4.10451000 L

H-HC -1 -7.13070700 -5.48923600 -4.27211200 L

H-HC -1 -8.25152400 -5.80391900 -5.60039100 L

C-CT -1 -8.27971000 -7.05626700 -2.39709800 L

H-HC -1 -8.32291200 -8.04634000 -1.94710000 L

H-HC -1 -7.29824800 -6.63380900 -2.16723900 L

H-HC -1 -9.04570800 -6.41668400 -1.96346900 L

C-C -1 -9.97459300 -7.95247700 -5.83543900 L

O-O -1 -10.51167200 -7.12706200 -6.57669700 L

N-N -1 -9.61332100 -9.17003900 -6.30555300 L

C-CT -1 -8.76126300 -10.15151400 -5.64636300 L

H-H1 -1 -9.33718200 -10.68865000 -4.90058200 L

H-H1 -1 -7.88685300 -9.70012800 -5.18704400 L

C-CT -1 -8.30872400 -11.11922500 -6.73334200 L

H-HC -1 -8.13078000 -12.11626600 -6.32939600 L

H-HC -1 -7.41467600 -10.73504100 -7.22277400 L

C-CT -1 -9.47860200 -11.09871400 -7.70907800 L

H-HC -1 -10.25761100 -11.77135300 -7.34491000 L

H-HC -1 -9.17427300 -11.38319300 -8.71529200 L

C-CT -1 -9.95304400 -9.64316600 -7.65150800 L

H-H1 -1 -9.39362400 -9.05795400 -8.38060800 L

C-C -1 -11.44460000 -9.54330000 -7.99295100 L

O-O -1 -11.78894600 -9.06647800 -9.07262000 L

N-N -1 -13.64889100 -7.40911200 -6.72046700 L

H-H -1 -12.96225700 -7.69186400 -6.02623800 L

C-CT -1 -13.94131900 -5.97277700 -6.88752300 L

H-H1 -1 -15.01997600 -5.87130600 -6.95313500 L

C-CT -1 -13.52599400 -5.13528800 -5.66489800 L

H-HC -1 -12.45686000 -5.24267400 -5.48693100 L

C-CT -1 -13.87261300 -3.65124700 -5.86364700 L

H-HC -1 -14.92598500 -3.54193100 -6.12210400 L

H-HC -1 -13.68535500 -3.09839200 -4.94340300 L

H-HC -1 -13.26273100 -3.22050700 -6.65537200 L

C-CT -1 -14.29020000 -5.58399900 -4.41472700 L

H-HC -1 -14.10297900 -6.63117600 -4.19550400 L

H-HC -1 -13.96744000 -5.00123400 -3.55342800 L

H-HC -1 -15.36231700 -5.44930300 -4.55727700 L

C-C -1 -13.36650200 -5.42751600 -8.20265300 L

O-O -1 -14.07566000 -4.72070000 -8.91670500 L

N-N -1 -12.15364000 -5.82875900 -8.61236400 L

H-H -1 -11.59623200 -6.39790400 -7.98065700 L

C-CT -1 -11.62437400 -5.53385600 -9.96555000 L

H-H1 -1 -11.58870200 -4.45216100 -10.09497800 L

C-CT -1 -10.18813500 -6.08078000 -10.15133000 L

H-HC -1 -10.17163000 -7.14031400 -9.90270700 L

C-CT -1 -9.65855800 -5.91572000 -11.58324200 L

H-HC -1 -9.66788800 -4.86461800 -11.86928700 L

H-HC -1 -8.63922500 -6.29735700 -11.64663500 L

H-HC -1 -10.26884600 -6.48174200 -12.28371000 L

C-CT -1 -9.19342500 -5.35413800 -9.24001900 L

H-HC -1 -9.45608900 -5.51768500 -8.19810100 L

H-HC -1 -8.18810800 -5.74501600 -9.39823900 L

H-HC -1 -9.20038900 -4.28441100 -9.44755500 L

C-C -1 -12.56541400 -6.08072600 -11.04989900 L

O-O -1 -12.82635500 -5.40053100 -12.04130900 L

N-N -1 -15.77569500 -4.02237700 -11.15931800 L

H-H -1 -15.01914700 -4.53988700 -10.72416200 L

C-CT -1 -15.42600000 -2.82662500 -11.93636000 L

H-H1 -1 -16.21578800 -2.09348300 -11.81202300 L

C-CT -1 -14.11744100 -2.18150900 -11.41349600 L

H-HC -1 -13.34858000 -2.95097400 -11.34516700 L

C-CT -1 -13.62760400 -1.09637200 -12.38977400 L

H-HC -1 -14.42554900 -0.38694700 -12.59239600 L

H-HC -1 -12.76495700 -0.56785400 -11.98538700 L

H-HC -1 -13.32039300 -1.54478600 -13.33357200 L

C-CT -1 -14.33321700 -1.57128800 -10.00612800 L

H-HC -1 -14.91127300 -0.65170200 -10.09090500 L

H-HC -1 -14.90406400 -2.26226800 -9.38801300 L

C-CT -1 -13.02853400 -1.26422900 -9.25660300 L

H-HC -1 -12.46276100 -0.48476500 -9.76746500 L

H-HC -1 -13.26577700 -0.91639300 -8.25070300 L

H-HC -1 -12.42053600 -2.16589100 -9.18332200 L

C-C -1 -15.39503600 -3.14516700 -13.43661900 L

O-O -1 -16.15136500 -2.54259200 -14.19536700 L

N-N -1 -6.85519600 -0.02832400 -14.14465800 L

H-H -1 -6.52729600 -0.86582000 -14.59723500 L

C-CT -1 -7.14477200 -0.12277200 -12.70363900 L

H-H1 -1 -7.53391400 0.83098600 -12.35069900 L

C-CT -1 -8.21868600 -1.20870000 -12.43002600 L

H-HC -1 -7.79197800 -2.17326000 -12.71389900 L

C-CT -1 -8.56291900 -1.26301800 -10.92649000 L

H-HC -1 -8.93562400 -0.29414100 -10.59031900 L

H-HC -1 -9.32116800 -2.01946300 -10.73084400 L

H-HC -1 -7.68555200 -1.52816500 -10.33641800 L

C-CT -1 -9.51287800 -1.00202200 -13.25503700 L

H-HC -1 -10.08095700 -0.16383400 -12.85323900 L

H-HC -1 -9.26684100 -0.77128100 -14.29107100 L

C-CT -1 -10.40523200 -2.24844400 -13.29380700 L

H-HC -1 -10.83720500 -2.45206900 -12.31492700 L

H-HC -1 -11.21066900 -2.08270300 -14.00652900 L

H-HC -1 -9.82475200 -3.10957900 -13.62542200 L

C-C -1 -5.85324000 -0.43727100 -11.93528000 L

O-O -1 -5.12653900 -1.35533300 -12.32207100 L

N-N -1 -5.59489600 0.26488400 -10.82981700 L

H-H -1 -6.21530900 1.04008100 -10.60875200 L

C-CT -1 -4.56017400 -0.07565800 -9.83133300 L

H-H1 -1 -3.92691400 -0.85849800 -10.23454400 L

C-CT -1 -3.65642900 1.13559900 -9.52792900 L

H-HC -1 -4.27061000 1.95842400 -9.16167900 L

C-CT -1 -2.58787600 0.81312500 -8.47414600 L

H-HC -1 -1.98854400 -0.04151100 -8.78845800 L

H-HC -1 -1.93759900 1.67446100 -8.32908900 L

H-HC -1 -3.05699500 0.58807400 -7.51581200 L

C-CT -1 -2.92335800 1.59818400 -10.79336800 L

H-HC -1 -3.64095000 1.93209900 -11.54289900 L

H-HC -1 -2.26351400 2.43419200 -10.55860000 L

H-HC -1 -2.33325100 0.77615500 -11.19645400 L

C-C -1 -5.22835900 -0.61363600 -8.56204100 L

O-O -1 -6.19876400 -0.01172000 -8.10452500 L

N-N -1 -4.74222300 -1.72853400 -8.00102200 L

H-H -1 -3.93934100 -2.17779400 -8.42535000 L

C-CT -1 -5.37669000 -2.40495000 -6.85940600 L

H-H1 -1 -6.06852500 -1.70949500 -6.38654400 L

C-CT -1 -6.20009700 -3.58593200 -7.38892400 L

H-HC -1 -5.53138200 -4.33449200 -7.81318200 L

H-HC -1 -6.75724700 -4.03726200 -6.56860700 L

H-HC -1 -6.89966400 -3.24121000 -8.15111400 L

C-C -1 -4.40684300 -2.86819200 -5.75045400 L

O-O -1 -3.25812400 -3.22745200 -6.00830900 L

N-N 0 -4.95761200 -3.01620000 -4.55719600 L

H-H 0 -5.92899900 -2.77399400 -4.42354300 L

C-CT 0 -4.19872700 -3.39338800 -3.39618800 L

H-H1 0 -3.24279900 -2.87430900 -3.44358700 L

C-CT 0 -5.00377600 -2.87067100 -2.21648500 L

H-HC 0 -5.44321600 -1.90274300 -2.46837900 L

H-HC 0 -5.82228300 -3.56241900 -2.00648900 L

C-C 0 -4.18089700 -2.66011600 -0.96621500 L

O-O 0 -2.95255800 -2.63698000 -1.00796400 L

O-O 0 -4.83824800 -2.52858800 0.04590300 L H-H 2506 0.0000

C-C 0 -3.92238600 -4.90136100 -3.30965100 L

O-O 0 -4.63551800 -5.72325500 -3.88517600 L

N-N 0 -2.87175800 -5.24013800 -2.55417600 L

H-H 0 -2.33262400 -4.49411100 -2.13825200 L

C-CT 0 -2.50167000 -6.58867000 -2.15052400 L

H-H1 0 -3.39001500 -7.21830500 -2.14005100 L

H-H1 0 -1.79269700 -6.99070600 -2.87407700 L

C-C 0 -1.86810200 -6.60823000 -0.75236600 L

O-O 0 -1.21819300 -7.59241600 -0.40061500 L

N-N 0 -2.08499600 -5.53706700 0.03371900 L

H-H 0 -2.64564300 -4.79193600 -0.35424900 L

C-CT 0 -1.60212900 -5.29386400 1.39178500 L

H-H1 0 -1.68177000 -4.22420200 1.55490000 L

C-CT 0 -2.54404200 -5.96017300 2.41929700 L

H-HC 0 -2.55328800 -7.04139800 2.27212600 L

H-HC 0 -2.18211600 -5.74953400 3.42568600 L

C-CT 0 -3.97460300 -5.40757200 2.30841300 L

H-HC 0 -3.92644500 -4.32223500 2.24357700 L

H-HC 0 -4.43808400 -5.78254000 1.39548200 L

C-C 0 -4.84762600 -5.79239600 3.50347900 L

O-O2 0 -5.02020000 -7.00953500 3.71219800 L

O-O2 0 -5.34915200 -4.87004800 4.18187600 L

C-C 0 -0.10621300 -5.61672800 1.54407700 L

O-O 0 0.67687700 -5.33223100 0.63708600 L

N-N 0 0.28993400 -6.18384500 2.69141000 L

H-H 0 -0.40539600 -6.35707300 3.40293200 L

C-CT 0 1.62099000 -6.71127000 2.95772800 L

H-H1 0 2.33758400 -5.89264400 2.93690400 L

C-CT 0 1.62135800 -7.32572100 4.36077300 L

H-HC 0 0.79844100 -8.03496500 4.45658500 L

H-HC 0 2.55781500 -7.85540500 4.52962000 L

H-HC 0 1.50517100 -6.54061300 5.10913200 L

C-C 0 2.00920900 -7.75505500 1.90255800 L

O-O 0 3.07543700 -7.66658000 1.29441100 L

N-N 0 1.12121100 -8.73697100 1.71245600 L

H-H 0 0.27181200 -8.70027900 2.25760700 L

C-CT 0 1.25627200 -9.89662500 0.84572700 L

H-H1 0 1.00582400 -9.60760800 -0.17471900 L

H-H1 0 2.28676700 -10.25254100 0.87122300 L

C-C 0 0.32406800 -11.03238600 1.29066000 L

O-O 0 0.29258900 -12.07100400 0.63132900 L

N-N 0 -0.41053300 -10.82880600 2.40351700 L

H-H 0 -0.31237400 -9.92906500 2.85142100 L

C-CT 0 -1.28143100 -11.76265600 3.11767000 L

H-H1 0 -1.67402700 -11.20504400 3.96878700 L

C-CT 0 -2.50141200 -12.14854200 2.26444200 L

H-HC 0 -2.17216200 -12.59735000 1.32710600 L

H-HC 0 -3.07069800 -12.91287200 2.79468100 L

C-CA 0 -3.44312600 -10.99520900 1.96794300 L

C-CA 0 -4.52580200 -10.73374300 2.83160600 L

H-HA 0 -4.67538100 -11.33392400 3.71739700 L

C-CA 0 -5.44022300 -9.71016500 2.52795400 L

H-HA 0 -6.28167000 -9.52555600 3.17985300 L

C-CA 0 -5.26119500 -8.92912500 1.37274300 L

H-HA 0 -5.96105200 -8.14327900 1.13556600 L

C-CA 0 -4.16342000 -9.16335700 0.52940800 L

H-HA 0 -4.02773000 -8.55559500 -0.35086600 L

C-CA 0 -3.25753300 -10.19770600 0.82174000 L

H-HA 0 -2.42643800 -10.38230800 0.15678000 L

C-C 0 -0.56275500 -12.97956900 3.72674600 L

O-O 0 -1.19934900 -13.73791700 4.45931400 L

N-N -1 0.74177300 -13.16220200 3.46805100 L

H-H -1 1.21716700 -12.51533200 2.85196600 L

C-CT -1 1.51764000 -14.28825700 3.99470600 L

H-H1 -1 1.78187300 -14.09085700 5.03271500 L

H-H1 -1 0.90237700 -15.18813900 3.96335600 L

C-C -1 2.80214900 -14.55132600 3.20362500 L

O-O -1 3.32942900 -13.66437300 2.53666700 L

N-N -1 3.30772400 -15.78983300 3.25718100 L

H-H -1 2.85255000 -16.47197900 3.84327000 L

C-CT -1 4.45391600 -16.22002100 2.44174200 L

H-H1 -1 5.31978700 -15.61249700 2.70608100 L

H-H1 -1 4.69097200 -17.26204400 2.65709300 L

C-C -1 4.18548200 -16.07150700 0.93555200 L

O-O -1 3.04056900 -16.18612900 0.49445200 L

N-N -1 5.23275100 -15.83361200 0.13939800 L

H-H -1 6.14302100 -15.80320000 0.57476000 L

C-CT -1 5.14520200 -15.25306400 -1.21152100 L

H-H1 -1 4.63120300 -14.29462000 -1.11378600 L

C-CT -1 6.57577500 -14.95312700 -1.68154900 L

H-HC -1 7.13359800 -15.88169900 -1.81184100 L

H-HC -1 6.54639600 -14.42148500 -2.63239500 L

H-HC -1 7.08561100 -14.32072400 -0.95314200 L

C-C -1 4.35189200 -16.05010700 -2.27919100 L

O-O -1 4.07153200 -15.51052100 -3.35129700 L

N-N -1 1.19944700 -16.66894100 -1.72204200 L

H-H -1 1.84097200 -16.64451900 -0.93913100 L

C-CT -1 -0.03972600 -15.88986500 -1.62455600 L

H-H1 -1 -0.88021700 -16.51811600 -1.92922200 L

C-CT -1 -0.25988800 -15.44980700 -0.16774700 L

H-HC -1 0.59711900 -14.87604700 0.18603100 L

H-HC -1 -1.13509600 -14.80140900 -0.11565400 L

C-C -1 -0.50298400 -16.62636400 0.75399600 L

O-O -1 -1.57879800 -17.19787100 0.80307600 L

N-N -1 0.50408100 -17.08259200 1.45992400 L

H-H -1 0.32147400 -17.87889400 2.04233100 L

H-H -1 1.41829200 -16.66473100 1.34050400 L

C-C -1 -0.01563000 -14.68785500 -2.57951500 L

O-O -1 -0.99250700 -14.44877000 -3.28383200 L

N-N -1 1.12912400 -13.99851000 -2.67612800 L

H-H -1 1.87589400 -14.24097500 -2.04584800 L

C-CT -1 1.37274800 -12.93168400 -3.66433200 L

H-H1 -1 0.62549300 -12.14774400 -3.53085900 L

C-CT -1 2.77121900 -12.30828800 -3.46650800 L

H-HC -1 3.52152700 -13.08839600 -3.55405700 L

C-CT -1 3.09559500 -11.23371900 -4.50919400 L

H-HC -1 2.32677600 -10.46370600 -4.51246500 L

H-HC -1 4.05221200 -10.76935200 -4.27782800 L

H-HC -1 3.16082400 -11.67361000 -5.50312700 L

C-CT -1 2.91882700 -11.67863900 -2.07745200 L

H-HC -1 2.65556700 -12.38587700 -1.29103900 L

H-HC -1 3.95178100 -11.37071300 -1.92102300 L

H-HC -1 2.27220500 -10.80450800 -2.00600700 L

C-C -1 1.22576500 -13.46510100 -5.09159500 L

O-O -1 0.61286600 -12.80050100 -5.92050800 L

N-N -1 1.72420000 -14.67600100 -5.37139500 L

H-H -1 2.21385400 -15.17474600 -4.64398800 L

C-CT -1 1.65943200 -15.28458900 -6.70367000 L

H-H1 -1 2.10883400 -14.59088200 -7.41583000 L

C-CT -1 2.48296000 -16.58226900 -6.72193200 L

H-HC -1 3.46719200 -16.38972400 -6.29806600 L

H-HC -1 2.00504800 -17.32666500 -6.08676400 L

C-CA -1 2.67358600 -17.17466200 -8.10281400 L

C-CA -1 1.82330700 -18.20238100 -8.55721500 L

H-HA -1 1.02432300 -18.56572100 -7.92419500 L

C-CA -1 2.01478900 -18.76333200 -9.83484000 L

H-HA -1 1.36716200 -19.55501300 -10.17724600 L

C-C -1 3.05902700 -18.29026700 -10.66093200 L

O-OH -1 3.26104300 -18.83267500 -11.88908800 L

H-HO -1 2.67479300 -19.56955500 -12.06407100 L

C-CA -1 3.90267500 -17.25632100 -10.20765500 L

H-HA -1 4.69140700 -16.90125200 -10.85269500 L

C-CA -1 3.71232300 -16.69941800 -8.92887400 L

H-HA -1 4.35866800 -15.90372700 -8.57981500 L

C-C -1 0.21216900 -15.53498100 -7.15411800 L

O-O -1 -0.19631400 -15.04969200 -8.20887100 L

N-N -1 -2.58132300 -14.25691700 -5.71940600 L

H-H -1 -1.84516900 -14.41536300 -5.03796700 L

C-CT -1 -3.31207900 -12.98339600 -5.67230100 L

H-H1 -1 -4.37654200 -13.21663200 -5.70067200 L

C-CT -1 -3.01690000 -12.25703900 -4.34586600 L

H-HC -1 -3.28142700 -12.92438600 -3.52317500 L

H-HC -1 -1.94598200 -12.06122100 -4.28527400 L

C-CT -1 -3.76871000 -10.92261000 -4.15391600 L

H-HC -1 -3.45467400 -10.21597600 -4.92115900 L

C-CT -1 -5.29017900 -11.08212300 -4.22375400 L

H-HC -1 -5.62224300 -11.83444800 -3.50894400 L

H-HC -1 -5.76646900 -10.13009400 -3.98547100 L

H-HC -1 -5.58929700 -11.36943800 -5.23021200 L

C-CT -1 -3.42119600 -10.33370000 -2.78728400 L

H-HC -1 -2.34915000 -10.15393300 -2.72085600 L

H-HC -1 -3.93469800 -9.38050400 -2.65217000 L

H-HC -1 -3.71902100 -11.01678100 -1.99233700 L

C-C -1 -3.01483800 -12.10738700 -6.89856000 L

O-O -1 -3.94752100 -11.59955800 -7.51724800 L

N-N -1 -1.74868300 -11.97654700 -7.30510100 L

H-H -1 -1.01263100 -12.41700900 -6.75997500 L

C-CT -1 -1.36352700 -11.24645300 -8.51842600 L

H-H1 -1 -1.78608800 -10.24339900 -8.46032000 L

C-CT -1 0.17057500 -11.13964000 -8.56768900 L

H-HC -1 0.52623600 -10.67018600 -7.64917000 L

H-HC -1 0.58232800 -12.14919300 -8.60337000 L

C-CT -1 0.73879800 -10.35612900 -9.76259600 L

H-HC -1 1.80630700 -10.55862700 -9.80115300 L

H-HC -1 0.31073200 -10.71856300 -10.69316200 L

C-C -1 0.55205900 -8.84192800 -9.68236900 L

O-O -1 -0.47740600 -8.31430700 -9.28974700 L

N-N -1 1.54903100 -8.06744200 -10.04578000 L

H-H -1 1.42599700 -7.07404400 -9.93808600 L

H-H -1 2.43822400 -8.48230600 -10.31548400 L

C-C -1 -1.94072300 -11.92067300 -9.77314300 L

O-O -1 -2.51655600 -11.24296700 -10.62355500 L

N-N -1 -5.72640300 -11.03262800 -9.67916200 L

H-H -1 -4.94667100 -11.26545400 -9.07073300 L

C-CT -1 -5.89483500 -9.63226800 -10.09010700 L

H-H1 -1 -6.91684400 -9.32059000 -9.87713600 L

C-CT -1 -4.91863300 -8.74232700 -9.29133700 L

H-HC -1 -3.91317500 -9.15245100 -9.39509000 L

H-HC -1 -4.91881300 -7.75253400 -9.74141300 L

C-CT -1 -5.23727600 -8.55931400 -7.79489000 L

H-HC -1 -5.41164900 -9.52461500 -7.32709600 L

C-CT -1 -4.06010900 -7.88268300 -7.09105200 L

H-HC -1 -3.86730400 -6.90341200 -7.52794100 L

H-HC -1 -4.28232900 -7.76030100 -6.02945000 L

H-HC -1 -3.16765100 -8.50204300 -7.18723800 L

C-CT -1 -6.47249000 -7.68284000 -7.58161000 L

H-HC -1 -7.34622700 -8.14410600 -8.03379000 L

H-HC -1 -6.65026200 -7.56676500 -6.51289600 L

H-HC -1 -6.31352400 -6.69900200 -8.02131400 L

C-C -1 -5.68826200 -9.46351200 -11.60846300 L

O-O -1 -6.47286200 -8.77995500 -12.26787300 L

N-N -1 -6.73186100 -6.37482500 -13.92355000 L

H-H -1 -6.51763700 -7.30513600 -13.57749200 L

C-CT -1 -5.98825500 -5.23626300 -13.34686600 L

H-H1 -1 -6.70702900 -4.43166400 -13.19225100 L

C-CT -1 -5.40337400 -5.62050400 -11.96701900 L

H-HC -1 -6.06784900 -6.34830600 -11.50227100 L

C-CT -1 -3.99346600 -6.23243300 -12.03195900 L

H-HC -1 -3.27325900 -5.51149200 -12.41689400 L

H-HC -1 -3.66084200 -6.52279300 -11.03705500 L

H-HC -1 -4.00289200 -7.11099500 -12.67571000 L

C-CT -1 -5.38441100 -4.42005700 -11.02535100 L

H-HC -1 -6.38957700 -4.01676300 -10.90600000 L

H-HC -1 -5.01026200 -4.72785700 -10.04995400 L

H-HC -1 -4.72778200 -3.65068800 -11.41641800 L

C-C -1 -4.90770300 -4.71176800 -14.31044300 L

O-O -1 -4.36957800 -5.47250900 -15.11175800 L

N-N -1 -2.24993000 -2.66432100 -12.74604700 L

H-H -1 -3.18024600 -2.52190900 -12.37111500 L

C-CT -1 -1.14169900 -2.66888300 -11.79692200 L

H-H1 -1 -0.43301600 -3.44377200 -12.08682800 L

H-H1 -1 -0.63489600 -1.70647800 -11.82207200 L

C-C -1 -1.57213200 -2.93947300 -10.35832600 L

O-O -1 -2.75786100 -2.88993000 -10.03375800 L

N-N -1 -0.61052100 -3.23412700 -9.49103300 L

H-H -1 0.35351700 -3.23754900 -9.81099400 L

C-CT -1 -0.86703900 -3.59520800 -8.09391400 L

H-H1 -1 -1.79925500 -3.11936400 -7.80734300 L

C-CT -1 -1.07337700 -5.11012400 -7.96601800 L

H-H1 -1 -1.22712600 -5.37153100 -6.91654900 L

H-H1 -1 -1.96832700 -5.38748200 -8.52339500 L

O-OH -1 0.03195300 -5.83552200 -8.48359800 L

H-HO -1 -0.29339500 -6.73706400 -8.69884100 L

C-C -1 0.19448500 -3.06693300 -7.13114300 L

O-O -1 1.38007700 -3.00823600 -7.47274600 L

N-N 0 -0.22701400 -2.71242100 -5.90991100 L

H-H 0 -1.20778100 -2.80181400 -5.68677500 L

C-CT 0 0.63276800 -2.14745200 -4.87782400 L

H-H1 0 1.63766700 -2.04765100 -5.28382000 L

C-CT 0 0.18710700 -0.72854300 -4.49576800 L

H-HC 0 0.78271500 -0.39222900 -3.64623000 L

H-HC 0 0.40899000 -0.06207000 -5.32816600 L

C-CC 0 -1.27535400 -0.56089400 -4.17407400 L

N-NB 0 -2.26144400 -0.25740700 -5.11033100 L

C-CR 0 -3.38561800 -0.09498300 -4.40201600 L

H-H5 0 -4.34449200 0.15069600 -4.83143100 L

N-NA 0 -3.15387200 -0.26581400 -3.08805300 L

H-H 0 -3.83885500 -0.18676400 -2.34992400 L

C-CW 0 -1.82342200 -0.56974200 -2.92475500 L

H-H4 0 -1.30686100 -0.73880200 -1.99135800 L

C-C 0 0.74080600 -3.09004200 -3.68207700 L

O-O 0 -0.24232200 -3.67326600 -3.22433700 L

N-N 0 1.98649400 -3.22595300 -3.21392300 L

H-H 0 2.71031600 -2.68179200 -3.66130500 L

C-CT 0 2.45186800 -4.16512900 -2.20539900 L

H-H1 0 1.60618000 -4.54231700 -1.63102900 L

C-CT 0 3.18527300 -5.33690700 -2.87579400 L

H-HC 0 4.14457800 -4.98206000 -3.25722500 L

H-HC 0 3.39658500 -6.08264700 -2.11026900 L

C-C* 0 2.47271500 -6.00996600 -4.00632100 L

C-CW 0 2.55297400 -5.64413800 -5.30546300 L

H-H4 0 3.14024500 -4.82243900 -5.68847100 L

N-NA 0 1.75860700 -6.46891400 -6.06812400 L

H-H 0 1.66938100 -6.36622900 -7.06885400 L

C-CN 0 1.12049200 -7.41549600 -5.29565900 L

C-CA 0 0.22319000 -8.45086500 -5.59449600 L

H-HA 0 -0.10829400 -8.61283000 -6.60829200 L

C-CA 0 -0.23003000 -9.27968500 -4.55722600 L

H-HA 0 -0.90638100 -10.08866000 -4.78036500 L

C-CA 0 0.20963200 -9.06062900 -3.23869500 L

H-HA 0 -0.12726200 -9.70576300 -2.44133100 L

C-CA 0 1.09417100 -8.00640400 -2.94745600 L

H-HA 0 1.41722900 -7.84875900 -1.92880200 L

C-CB 0 1.57288900 -7.15700900 -3.96812600 L

C-C 0 3.42738300 -3.42553500 -1.29755400 L H-H 886 0.0000

O-O 0 4.24892800 -2.65726600 -1.79832200 L

N-N 0 3.36793300 -3.68630500 0.00649700 H

H-H 0 2.63827500 -4.26685700 0.33340500 H

C-CM 0 4.05974900 -2.92147800 0.98502900 H

H-H 0 4.85910100 -2.29235300 0.46716600 H

C-CT 0 3.19372600 -1.89566200 1.74561800 H

H-HC 0 3.88279100 -1.22302200 2.31923700 H

H-HC 0 2.67806000 -1.25472000 0.97996300 H

C-CT 0 2.15847700 -2.48320500 2.67577400 H

H-HC 0 2.00587100 -3.57120800 2.46247100 H

H-HC 0 2.49487400 -2.38612900 3.73833600 H

C-C 0 0.82881400 -1.75833200 2.50659000 H

O-O2 0 -0.02462200 -2.25151700 1.69891500 H

O-O2 0 0.59725700 -0.67970600 3.13390000 H

C-C 0 4.83319700 -3.77831800 2.00369900 H

O-O 0 4.53720000 -4.96108400 2.17228100 H

N-N 0 5.83251400 -3.09034900 2.59764900 L H-H1 899 0.0000

H-H 0 5.93673700 -2.11444700 2.35880200 L

C-CT 0 6.86798700 -3.64316000 3.45292500 L

H-H1 0 7.18914700 -4.59292300 3.02538300 L

C-CT 0 8.06317700 -2.67494000 3.40585000 L

H-HC 0 8.92337900 -3.16720300 3.86067500 L

H-HC 0 8.30867000 -2.48086900 2.36141600 L

C-C 0 7.83974400 -1.32675200 4.10796800 L

O-O2 0 6.67398500 -0.96694800 4.38697800 L

O-O2 0 8.86509300 -0.66150200 4.36110900 L

C-C 0 6.41087200 -3.90212300 4.89764000 L

O-O 0 7.26409800 -4.06075000 5.76742800 L

N-N 0 5.09516900 -3.94384500 5.16307200 L

H-H 0 4.43747200 -3.79718400 4.41067900 L

C-CT 0 4.54241200 -4.24709900 6.47799100 L

H-H1 0 5.20782100 -3.83467300 7.23347500 L

C-CT 0 3.16029200 -3.59696600 6.65154400 L

H-HC 0 2.53193300 -3.82562100 5.78910100 L

H-HC 0 2.68097400 -4.01050700 7.54026000 L

C-CT 0 3.26230600 -2.07984400 6.84286400 L

H-HC 0 3.94609800 -1.86293200 7.66523800 L

H-HC 0 3.66220800 -1.62758500 5.93582400 L

C-C 0 1.89504600 -1.47911100 7.16520100 L

O-O 0 1.56431700 -1.26096600 8.32912200 L

N-N 0 1.09119200 -1.22242100 6.13226500 L

H-H 0 0.17409700 -0.83048900 6.29034600 L

H-H 0 1.40028500 -1.41986500 5.19109500 L

C-C 0 4.43183200 -5.75699400 6.70688600 L

O-O 0 4.37453000 -6.54778800 5.76607500 L

N-N 0 4.35727600 -6.12584600 7.99051800 L

H-H 0 4.43826500 -5.40184500 8.68844400 L

C-CT 0 3.94344600 -7.42471100 8.49074700 L

H-H1 0 4.47789800 -8.20102700 7.94251500 L

C-CT 0 4.31933600 -7.49234800 9.98251700 L

H-HC 0 5.39926900 -7.36327900 10.07651600 L

H-HC 0 3.84035600 -6.65557500 10.49421700 L

C-CT 0 3.90971500 -8.79436200 10.69757800 L

H-HC 0 2.82788300 -8.90295400 10.63875800 L

C-CT 0 4.56510300 -10.03801500 10.07518700 L

H-HC 0 5.64898600 -9.92288600 10.05723600 L

H-HC 0 4.30982200 -10.92271300 10.65905300 L

H-HC 0 4.20438000 -10.18580800 9.05774500 L

C-CT 0 4.28754100 -8.69741600 12.18229500 L

H-HC 0 3.82720600 -7.81765500 12.62648600 L

H-HC 0 3.94289500 -9.58357300 12.71543100 L

H-HC 0 5.36532100 -8.61193200 12.29203500 L

C-C 0 2.43232600 -7.58051700 8.27731400 L

O-O 0 1.65929500 -6.68351500 8.61614600 L

N-N 0 2.02783300 -8.72896700 7.71726700 L

H-H 0 2.72638200 -9.41630200 7.47313500 L

C-CT 0 0.64170100 -9.07297500 7.41376400 L

H-H1 0 0.21360400 -8.26572300 6.81836600 L

C-CT 0 0.61325600 -10.35232500 6.56880500 L

H-HC 0 1.03215500 -11.18516100 7.13518200 L

H-HC 0 -0.41566600 -10.58730500 6.29436500 L

H-HC 0 1.19653500 -10.21029200 5.65835800 L

C-C 0 -0.21627500 -9.24618400 8.67530100 L

O-O 0 -1.38567300 -8.86099000 8.66291700 L

N-N -1 0.34821100 -9.83733700 9.74194400 L

H-H -1 1.33400200 -10.04249700 9.71343600 L

C-CT -1 -0.36204000 -10.13979600 10.99821200 L

H-H1 -1 -1.38003300 -10.43470900 10.74378300 L

C-CT -1 0.28758800 -11.34498300 11.68531200 L

H-H1 -1 -0.22221900 -11.53762200 12.63084200 L

H-H1 -1 0.18172000 -12.22246000 11.04711400 L

O-OH -1 1.66229400 -11.10955100 11.92758300 L

H-HO -1 1.96271800 -11.72640200 12.60464200 L

C-C -1 -0.50843300 -8.95330200 11.96796700 L

O-O -1 -1.19295200 -9.07484500 12.98214400 L

N-N -1 0.09837600 -7.80225900 11.65943700 L

H-H -1 0.65443300 -7.77427100 10.81187000 L

C-CT -1 -0.00960800 -6.54795500 12.42241600 L

H-H1 -1 -0.87808100 -6.60874100 13.07907500 L

C-CT -1 1.24505300 -6.34472700 13.30110000 L

H-HC -1 2.11092300 -6.25930500 12.64501900 L

H-HC -1 1.15324600 -5.40405600 13.84520500 L

C-CT -1 1.52712100 -7.45552600 14.33058500 L

H-HC -1 1.70536000 -8.39676500 13.80724200 L

H-HC -1 2.44720800 -7.19643000 14.85737400 L

C-C -1 0.41495100 -7.65237400 15.37039500 L

O-O2 -1 -0.40526300 -6.73732300 15.63561200 L

O-O2 -1 0.31142700 -8.73339800 15.99090500 L

C-C -1 -0.26019100 -5.32577700 11.50740600 L

O-O -1 -0.05587400 -4.18377400 11.93055400 L

N-N 0 -0.76022100 -5.55140900 10.27891500 L

H-H 0 -0.93926500 -6.51079700 10.01861000 L

C-CT 0 -0.98019500 -4.56084000 9.21893400 L

H-H1 0 -0.00100500 -4.17668500 8.92837100 L

C-CT 0 -1.58272400 -5.30334100 8.00909200 L

H-HC 0 -0.98790200 -6.20313000 7.84782100 L

H-HC 0 -2.59842100 -5.62484600 8.24347500 L

C-CT 0 -1.56558200 -4.52555000 6.68119700 L

H-HC 0 -0.72756300 -3.82740700 6.68403300 L

H-HC 0 -1.39525500 -5.24304300 5.87691600 L

C-CT 0 -2.88157600 -3.78780000 6.37211300 L

H-HC 0 -3.69975600 -4.50667500 6.38613100 L

H-HC 0 -3.09039800 -3.03082200 7.12321700 L

C-CT 0 -2.85626900 -3.12424300 4.99099000 L

H-HP 0 -2.69522100 -3.88829600 4.23278200 L

H-HP 0 -3.82179200 -2.65163400 4.81003600 L

N-N3 0 -1.80026800 -2.10720500 4.86961400 L

H-H 0 -0.89935400 -2.53638200 5.02567700 L

H-H 0 -1.82547900 -1.70419500 3.94370300 L

H-H 0 -1.95124700 -1.38233800 5.55633200 L

C-C 0 -1.85121000 -3.37607900 9.67789500 L

O-O 0 -2.69333100 -3.52608800 10.56291900 L

N-N 0 -1.62437900 -2.19758300 9.07288900 L

H-H 0 -0.91364600 -2.16394200 8.35593900 L

C-CT 0 -2.30054200 -0.93060600 9.36346100 L

H-H1 0 -3.27920900 -1.11926900 9.80477500 L

C-CT 0 -1.44122700 -0.10519600 10.33714900 L

H-HC 0 -0.46227200 0.02195600 9.87615000 L

H-HC 0 -1.88544900 0.88232900 10.44279700 L

C-CT 0 -1.23046000 -0.67939100 11.74621300 L

H-HC 0 -0.80546300 -1.67784200 11.67363800 L

H-HC 0 -0.50627200 -0.04882600 12.26103700 L

C-CT 0 -2.51650400 -0.70501600 12.58464600 L

H-HC 0 -2.92744800 0.30376900 12.63021100 L

H-HC 0 -3.25460300 -1.35776300 12.12231900 L

C-CT 0 -2.24819800 -1.17661100 14.01841500 L

H-HP 0 -1.45766600 -0.56528600 14.45375400 L

H-HP 0 -3.15703300 -1.04508400 14.60552900 L

N-N3 0 -1.85152200 -2.59438200 14.07946800 L

H-H 0 -1.00452300 -2.72979000 13.54600600 L

H-H 0 -1.69023100 -2.85828900 15.04088800 L

H-H 0 -2.58808600 -3.16647000 13.69162900 L

C-C 0 -2.47539900 -0.10235000 8.07831100 L H-H 1029 0.0000

O-O 0 -1.58628900 -0.09821100 7.22595300 L

N-N 0 -3.57834900 0.65200700 7.97482000 H

H-H -1 -4.18625400 0.60005400 8.78352400 H

C-CT -1 -3.57022100 1.89807600 7.20661700 H

H-H1 -1 -3.52347900 1.57705100 6.16878300 H

C-CT -1 -4.94438800 2.55795900 7.42494000 H

H-H1 -1 -5.67441900 2.02494200 6.81776700 H

H-H1 -1 -5.23091200 2.44586300 8.46665100 H

S-SH 0 -5.02140500 4.30463600 7.20292500 H

H-HS 0 -4.83497800 4.39826700 5.89208800 H

C-C -1 -2.37479500 2.84164100 7.46297100 H

O-O 0 -1.90872600 2.81272400 8.60750100 H

N-N 0 -1.88710200 3.56881600 6.43526100 L H-H1 1038 0.0000

H-H 0 -2.35368400 3.52776100 5.54144900 L

C-CT 0 -0.59969900 4.25761700 6.48997600 L

H-H1 0 0.12154700 3.63000300 7.01268900 L

H-H1 0 -0.24866900 4.39324400 5.47086000 L

C-C 0 -0.65520200 5.62871700 7.16721800 L

O-O 0 0.06286300 6.54059000 6.75838100 L

N-N 0 -1.50225600 5.75776800 8.19701000 L

H-H 0 -2.03761500 4.94342700 8.46122400 L

C-CT 0 -1.80515900 6.97099400 8.94853800 L

H-H1 0 -0.95265000 7.64883200 8.88702000 L

C-CT 0 -3.03110600 7.66851500 8.33565200 L

H-HC 0 -3.90678200 7.02766300 8.44859300 L

H-HC 0 -3.21854700 8.59813500 8.87441500 L

C-CC 0 -2.84027800 7.98220200 6.87224700 L

N-NB 0 -3.20790500 7.13988700 5.82146500 L

C-CR 0 -2.69603900 7.70896500 4.72301900 L

H-H5 0 -2.78373000 7.29918300 3.73063400 L

N-NA 0 -2.06107200 8.85669700 5.02135800 L

H-H 0 -1.59059500 9.46246000 4.36417100 L

C-CW 0 -2.13538200 9.04407600 6.38095900 L

H-H4 0 -1.67596900 9.83826500 6.95068400 L

C-C 0 -2.04055200 6.62344800 10.42472900 L

O-O 0 -1.74826100 7.44347300 11.29478200 L

N-N 0 -2.53620500 5.40420000 10.69964300 L

H-H 0 -2.76376000 4.79723100 9.92496500 L

C-CT 0 -2.64612200 4.81400900 12.02870400 L

H-H1 0 -3.21846500 5.48595600 12.66911900 L

C-CT 0 -3.36264000 3.45233300 11.94552000 L

H-HC 0 -2.72747600 2.77761300 11.37263300 L

H-HC 0 -3.44713700 3.04520400 12.95456600 L

C-CT 0 -4.76316800 3.45910500 11.30009200 L

H-HC 0 -4.68513000 3.84015500 10.28210400 L

C-CT 0 -5.29218000 2.01697500 11.22942900 L

H-HC 0 -5.39855300 1.60441400 12.23361600 L

H-HC 0 -6.26287100 2.00176500 10.73251900 L

H-HC 0 -4.60038700 1.39421300 10.66133300 L

C-CT 0 -5.75773200 4.33839100 12.07336000 L

H-HC 0 -5.43550700 5.37933900 12.04978500 L

H-HC 0 -6.74318900 4.27442700 11.61060400 L

H-HC 0 -5.82681200 4.00555400 13.10976300 L

C-C 0 -1.25669300 4.59784100 12.64241800 L

O-O 0 -0.27034000 4.41518500 11.92569400 L

N-N 0 -1.20728500 4.56174500 13.98108200 L

H-H 0 -2.05749100 4.71958500 14.50280600 L

C-CT 0 -0.02868200 4.17729400 14.74618400 L

H-H1 0 0.86258900 4.60946900 14.28936900 L

H-H1 0 -0.12548300 4.57236800 15.75759900 L

C-C 0 0.12407200 2.65437900 14.81998300 L

O-O 0 -0.73183200 1.90492100 14.34384700 L

N-N -1 1.23258300 2.20789400 15.42728600 L

H-H -1 1.86445900 2.89707900 15.82153500 L

C-CT -1 1.53487000 0.78244700 15.61072100 L

H-H1 -1 2.44119300 0.69361000 16.20769700 L

H-H1 -1 0.72014100 0.31301200 16.16353400 L

C-C -1 1.74976900 -0.01247000 14.31528600 L

O-O -1 1.43294300 -1.20168300 14.27647200 L

N-N 0 2.32675600 0.61400500 13.27859800 L

H-H 0 2.56738800 1.59112100 13.36452100 L

C-CT 0 2.72024300 -0.06639100 12.04607200 L

H-H1 0 1.92298400 -0.75444800 11.75975300 L

C-CT 0 2.90738900 0.94394800 10.89790100 L

H-HC 0 3.82368700 1.50331900 11.05311100 L

H-HC 0 3.00645500 0.38379200 9.96783400 L

C-CT 0 1.75622700 1.95761200 10.76851200 L

H-HC 0 0.81109000 1.45481400 10.94698400 L

H-HC 0 1.87124800 2.72511200 11.53513700 L

C-CT 0 1.68726800 2.63248600 9.39215000 L

H-HC 0 1.58050800 1.87380000 8.61530300 L

H-HC 0 0.80959100 3.27973500 9.37098200 L

C-CT 0 2.93309100 3.47870600 9.12206800 L

H-HP 0 3.05863100 4.18562100 9.94070600 L

H-HP 0 3.80751000 2.82948500 9.08035600 L

N-N3 0 2.82207000 4.22319800 7.85587700 L

H-H 0 2.01722400 4.83244900 7.89245700 L

H-H 0 3.65669600 4.77436800 7.71516500 L

H-H 0 2.71643900 3.57184000 7.09106300 L

C-C 0 4.00821800 -0.86689500 12.30504100 L

O-O 0 4.89611400 -0.39370700 13.01813000 L

N-N -1 4.10414800 -2.07616100 11.72791000 L

H-H -1 3.35594100 -2.39045000 11.12519300 L

C-CT -1 5.23279800 -3.00919000 11.93310000 L

H-H1 -1 6.03058800 -2.50785100 12.48055000 L

C-CT -1 4.79155200 -4.25382300 12.73924700 L

H-HC -1 4.04906800 -4.80050400 12.15885200 L

C-CT -1 5.96915000 -5.19934000 13.02242600 L

H-HC -1 6.77025500 -4.66472800 13.53001500 L

H-HC -1 5.63987400 -6.02738600 13.65010600 L

H-HC -1 6.35250500 -5.61634900 12.09165800 L

C-CT -1 4.14527000 -3.88733100 14.08175400 L

H-HC -1 3.21946700 -3.33555000 13.91495600 L

H-HC -1 3.89773400 -4.79437400 14.63484100 L

H-HC -1 4.82358100 -3.27974600 14.67348200 L

C-C -1 5.78194800 -3.44861400 10.57811100 L

O-O -1 4.99450300 -3.81368300 9.71211800 L

N-N -1 7.10406700 -3.44316200 10.39412700 L

H-H -1 7.68277000 -3.10836000 11.15666200 L

C-CT -1 7.79411400 -3.78400900 9.14364700 L

H-H1 -1 7.10814400 -3.64035900 8.31020900 L

C-CT -1 8.99862800 -2.84173500 8.94259000 L

H-HC -1 9.69206600 -3.00083300 9.76739200 L

H-HC -1 9.50964100 -3.12152900 8.02059200 L

C-CT -1 8.67484100 -1.34230400 8.87408600 L

H-HC -1 8.18743200 -1.04518800 9.79905800 L

C-CT -1 9.97288700 -0.54460200 8.74921300 L

H-HC -1 10.46600100 -0.77010700 7.80471100 L

H-HC -1 9.75104700 0.52062700 8.79500200 L

H-HC -1 10.63661100 -0.79026100 9.57763700 L

C-CT -1 7.76927700 -1.00030600 7.69115400 L

H-HC -1 6.78471600 -1.44676700 7.82161700 L

H-HC -1 7.65098900 0.07818700 7.59815500 L

H-HC -1 8.20492000 -1.37719000 6.76671700 L

C-C -1 8.28353200 -5.23942800 9.08918700 L

O-O -1 8.63823800 -5.83386900 10.11100200 L

N-N 0 8.46801900 -5.76131300 7.86633000 L

H-H 0 8.16843200 -5.21987000 7.06961500 L

C-CT 0 9.30813500 -6.92646600 7.56446900 L

H-H1 0 9.59895700 -7.37132700 8.51368800 L

C-CT 0 8.52661500 -8.01080100 6.77534400 L

H-HC 0 9.23877700 -8.75979700 6.42762400 L

C-CT 0 7.57305200 -8.72799400 7.74710400 L

H-HC 0 6.83816900 -8.02613200 8.13906000 L

H-HC 0 7.05472400 -9.53947900 7.23509500 L

H-HC 0 8.13700800 -9.15299100 8.57735000 L

C-CT 0 7.70639500 -7.54012100 5.55077800 L

H-HC 0 7.12433900 -8.38798200 5.18657600 L

H-HC 0 7.00273000 -6.76789700 5.85289400 L

C-CT 0 8.52563200 -7.02588300 4.36357700 L

H-HC 0 9.32781200 -7.72388700 4.13231800 L

H-HC 0 7.87506000 -6.93471300 3.49347000 L

H-HC 0 8.93952000 -6.04473600 4.58185300 L

C-C 0 10.61187100 -6.46971900 6.87185000 L

O-O 0 10.63695800 -5.37562200 6.30476400 L

N-N -1 11.71400700 -7.25286800 6.93358200 L

C-CT -1 11.84598200 -8.53336500 7.61318800 L

H-H1 -1 11.56520600 -9.33958200 6.93362800 L

H-H1 -1 11.25620000 -8.58317900 8.52633000 L

C-CT -1 13.32629600 -8.64432200 7.96486000 L

H-HC -1 13.63918500 -9.68286300 8.06888300 L

H-HC -1 13.53225400 -8.07853400 8.87490000 L

C-CT -1 14.00188200 -7.96487600 6.77718800 L

H-HC -1 14.10890300 -8.68328600 5.96178400 L

H-HC -1 14.97420000 -7.55654000 7.05393000 L

C-CT -1 13.01738500 -6.85662300 6.38623000 L

H-H1 -1 13.33430900 -5.93295700 6.87329000 L

C-C -1 13.00847700 -6.62831000 4.86933700 L

O-O -1 12.21679900 -7.22446200 4.13433000 L

N-N -1 13.94318700 -5.81823700 4.37822800 L

H-H -1 14.55776400 -5.34231200 5.04185600 L

C-CT -1 14.04312700 -5.41158400 2.96508400 L

H-H1 -1 13.12013800 -4.90436900 2.68786800 L

C-CT -1 15.19119400 -4.39709800 2.83511200 L

H-H1 -1 16.10451300 -4.79726500 3.26055800 L

C-CT -1 15.48226700 -3.92913100 1.41343000 L

H-HC -1 14.61172200 -3.42708200 0.99928900 L

H-HC -1 16.31140700 -3.22231000 1.44278500 L

H-HC -1 15.76503800 -4.77200600 0.78450400 L

O-OH -1 14.83169500 -3.24615300 3.55735700 L

H-HO -1 15.05554300 -3.41466600 4.49705900 L

C-C -1 14.19058800 -6.61685700 2.02023200 L

O-O -1 13.45262300 -6.72631800 1.03808100 L

N-N -1 15.02075900 -7.60423200 2.37379400 L

H-H -1 15.68535600 -7.39752300 3.11934000 L

C-CT -1 15.19129500 -8.88172900 1.66135700 L

H-H1 -1 15.43048500 -8.64715200 0.62469600 L

C-CT -1 16.38743300 -9.63526100 2.29003600 L

H-HC -1 17.19744700 -8.93621000 2.47099200 L

H-HC -1 16.07937200 -10.03084200 3.25928300 L

C-CT -1 16.98727000 -10.78417900 1.45658600 L

H-HC -1 17.77202500 -11.25791900 2.04604500 L

H-HC -1 16.23079600 -11.54344700 1.26499200 L

C-C -1 17.60805400 -10.34716700 0.13005800 L

O-O -1 17.81051400 -9.17625300 -0.15808200 L

N-N -1 17.94940100 -11.28499600 -0.72267000 L

H-H -1 18.33975200 -11.00315800 -1.61529400 L

H-H -1 17.86255700 -12.25849900 -0.46963600 L

C-C -1 13.92099800 -9.76212600 1.65025000 L

O-O -1 13.87783800 -10.76201200 0.93380800 L

N-N -1 12.88078300 -9.42917200 2.42464900 L

H-H -1 12.95997100 -8.59143700 2.99298700 L

C-CT -1 11.57292900 -10.09658300 2.39678500 L

H-H1 -1 11.69305300 -11.07155000 1.93157100 L

C-CT -1 11.09721100 -10.35164500 3.83803000 L

H-HC -1 11.90434400 -10.87203300 4.35381200 L

H-HC -1 10.92748100 -9.40948800 4.35539600 L

C-CT -1 9.80875300 -11.19556300 3.91190300 L

H-HC -1 8.94692900 -10.52921800 3.85280000 L

H-HC -1 9.76226900 -11.87458500 3.06014200 L

C-C -1 9.72073300 -12.05158100 5.17831600 L

O-O -1 10.69475500 -12.64123900 5.62479600 L

N-N -1 8.55336300 -12.20916700 5.76491900 L

H-H -1 8.51185100 -12.84132300 6.55588000 L

H-H -1 7.72833500 -11.77412400 5.39258400 L

C-C -1 10.56113900 -9.36091800 1.50870000 L

O-O -1 9.85522900 -10.02431600 0.74930600 L

N-N 0 10.55583400 -8.01824500 1.49614500 L

H-H 0 11.16844600 -7.50599700 2.11465100 L

C-CT 0 9.78679500 -7.26157700 0.51060900 L

H-H1 0 8.75759500 -7.61664200 0.58269600 L

C-CT 0 9.77116200 -5.76553600 0.83978700 L

H-HC 0 9.56607900 -5.63061400 1.90236600 L

H-HC 0 10.74896400 -5.33640100 0.62404500 L

C-CC 0 8.70235100 -5.03760200 0.05795300 L

N-NB 0 8.92436000 -4.10284500 -0.95344700 L

C-CR 0 7.70555100 -3.75515100 -1.39162400 L

H-H5 0 7.51723600 -3.03921300 -2.17629500 L

N-NA 0 6.74713500 -4.42543500 -0.72815200 L

H-H 0 5.75178700 -4.34164900 -0.87877700 L

C-CW 0 7.35828300 -5.24345200 0.19162600 L

H-H4 0 6.87181100 -5.92442400 0.87431700 L

C-C 0 10.27053400 -7.52502400 -0.92867200 L

O-O 0 9.44490800 -7.56134900 -1.83160100 L

N-N -1 11.57825500 -7.73050300 -1.15155800 L

H-H -1 12.20728500 -7.48479000 -0.39294600 L

C-CT -1 12.15769300 -8.16375700 -2.43754300 L

H-H1 -1 11.84258400 -7.45099200 -3.20126100 L

C-CT -1 13.70592500 -8.09754100 -2.35379400 L

H-HC -1 14.02203800 -8.60699300 -1.44131000 L

C-CT -1 14.39397000 -8.79087000 -3.54681400 L

H-HC -1 14.08368700 -8.32420000 -4.48333400 L

H-HC -1 15.47608800 -8.71730000 -3.45725500 L

H-HC -1 14.14514800 -9.85170400 -3.57281000 L

C-CT -1 14.15355200 -6.61430300 -2.28350900 L

H-HC -1 13.96293000 -6.13387800 -3.24478200 L

H-HC -1 13.56196300 -6.08739900 -1.53684700 L

C-CT -1 15.62835000 -6.40801300 -1.91238700 L

H-HC -1 16.28131000 -6.78165000 -2.70054300 L

H-HC -1 15.81730600 -5.34180400 -1.78994800 L

H-HC -1 15.85389100 -6.91650800 -0.97630800 L

C-C -1 11.61391700 -9.53902400 -2.89401800 L

O-O -1 11.47302200 -9.76321300 -4.09981800 L

N-N -1 11.21905100 -10.44270600 -1.97816800 L

H-H -1 11.27706200 -10.18489200 -1.00100600 L

C-CT -1 10.53689500 -11.70926700 -2.33642400 L

H-H1 -1 11.02661500 -12.12650000 -3.21799400 L

C-CT -1 10.64668900 -12.76835500 -1.21771800 L

H-HC -1 10.17927600 -12.40224200 -0.30253000 L

H-HC -1 10.11035100 -13.66172100 -1.53973900 L

C-CT -1 12.11091000 -13.14798000 -0.93908700 L

H-HC -1 12.61755600 -13.29188600 -1.89395200 L

H-HC -1 12.59465000 -12.32169000 -0.43105200 L

C-CT -1 12.31932500 -14.43531600 -0.12023000 L

H-H1 -1 11.93152800 -15.27497800 -0.69714800 L

H-H1 -1 13.39140200 -14.60103800 0.00193900 L

N-N2 -1 11.66750600 -14.44937000 1.20564400 L

H-H -1 10.88231300 -15.07647200 1.30699400 L

C-CA -1 12.11663300 -13.93840600 2.33825200 L

N-N2 -1 13.12816800 -13.12102500 2.41291900 L

H-H -1 13.51545000 -12.72000500 1.56869300 L

H-H -1 13.45651200 -12.82399000 3.30973000 L

N-N2 -1 11.51529100 -14.24101100 3.44591200 L

H-H -1 10.76089800 -14.92259300 3.42146300 L

H-H -1 11.69773100 -13.76022300 4.31744100 L

C-C -1 9.08358800 -11.47801100 -2.76819400 L

O-O -1 8.64213700 -12.08246000 -3.74511400 L

N-N 0 8.36931600 -10.55617200 -2.10041100 L

H-H 0 8.79853500 -10.09313100 -1.31194500 L

C-CT 0 7.05343900 -10.06263400 -2.51007400 L

H-H1 0 6.37583600 -10.91732000 -2.54895000 L

C-CT 0 6.50656800 -9.07694800 -1.45376000 L

H-H1 0 7.24074500 -8.29888800 -1.26686300 L

C-CT 0 5.20116500 -8.38616400 -1.87197200 L

H-HC 0 4.44283400 -9.12391600 -2.12819400 L

H-HC 0 4.83714900 -7.77028200 -1.04905800 L

H-HC 0 5.37797100 -7.73968600 -2.73183100 L

O-OH 0 6.28140300 -9.76621400 -0.23980300 L

H-HO 0 5.96882700 -9.13693400 0.41434000 L

C-C 0 7.11067300 -9.45048700 -3.92126900 L

O-O 0 6.25310800 -9.76551400 -4.73907400 L

N-N 0 8.09936800 -8.59337600 -4.21807400 L

H-H 0 8.78576900 -8.37817700 -3.51041200 L

C-CT 0 8.27805700 -7.96001600 -5.52398300 L

H-H1 0 7.32075900 -7.53590200 -5.82318800 L

C-CT 0 9.31152600 -6.82125300 -5.43850700 L

H-HC 0 10.23634200 -7.22655000 -5.02507000 L

H-HC 0 9.52650100 -6.46820800 -6.44865100 L

C-CT 0 8.85820700 -5.61379900 -4.58533500 L

H-HC 0 8.56388600 -5.96105400 -3.59685400 L

C-CT 0 10.03302600 -4.63721600 -4.41152500 L

H-HC 0 10.35814300 -4.26126300 -5.38257000 L

H-HC 0 9.72822100 -3.79581800 -3.78928600 L

H-HC 0 10.86785400 -5.14314200 -3.92494000 L

C-CT 0 7.65790800 -4.87041700 -5.19628100 L

H-HC 0 6.79705900 -5.53202100 -5.28075000 L

H-HC 0 7.37196700 -4.04431700 -4.54663600 L

H-HC 0 7.91574800 -4.48353200 -6.18301600 L

C-C 0 8.66635000 -8.98098400 -6.60306900 L

O-O 0 8.16803900 -8.87526400 -7.72176700 L

N-N -1 9.52806200 -9.95884500 -6.27484500 L

H-H -1 9.95106300 -9.90514400 -5.35557000 L

C-CT -1 9.90558000 -11.07291300 -7.16419900 L

H-H1 -1 10.33630400 -10.66813500 -8.08017200 L

C-CT -1 10.95696400 -11.98084500 -6.49949800 L

H-H1 -1 10.58301300 -12.33973300 -5.54147900 L

C-CT -1 11.34699400 -13.18448300 -7.35919400 L

H-HC -1 11.67404200 -12.84816200 -8.34346100 L

H-HC -1 12.15938400 -13.72784000 -6.87709700 L

H-HC -1 10.50114700 -13.86159200 -7.47235800 L

O-OH -1 12.15227700 -11.26323100 -6.29122800 L

H-HO -1 11.99078000 -10.63239300 -5.56835100 L

C-C -1 8.67431900 -11.89423800 -7.55871100 L

O-O -1 8.47115400 -12.16307600 -8.74057800 L

N-N -1 7.81198600 -12.24136600 -6.59747200 L

H-H -1 8.02870800 -11.98190500 -5.64000600 L

C-CT -1 6.57284400 -12.99382300 -6.84338700 L

H-H1 -1 6.81974300 -13.89379700 -7.40617800 L

C-CT -1 5.99058800 -13.42749300 -5.49611500 L

H-H1 -1 6.77926900 -13.89866500 -4.90669500 L

H-H1 -1 5.61579300 -12.56051800 -4.95236400 L

O-OH -1 4.95752500 -14.36497000 -5.69265900 L

H-HO -1 4.68593200 -14.73346600 -4.83296300 L

C-C -1 5.55106100 -12.19794100 -7.67170500 L

O-O -1 5.01301800 -12.70550200 -8.65952600 L

N-N -1 5.35227000 -10.91468400 -7.34244100 L

H-H -1 5.82245500 -10.55131300 -6.51990400 L

C-CT -1 4.45342300 -10.01058300 -8.05989700 L

H-H1 -1 3.46946500 -10.47865200 -8.10957900 L

C-CT -1 4.32463400 -8.71400300 -7.24961100 L

H-HC -1 5.29407400 -8.22288400 -7.16988000 L

H-HC -1 3.62167000 -8.03972400 -7.74099500 L

H-HC -1 3.95076500 -8.93328500 -6.24828500 L

C-C -1 4.90429500 -9.72542900 -9.50814200 L

O-O -1 4.06109000 -9.43633900 -10.36278100 L

N-N -1 6.20846800 -9.83297100 -9.80647300 L

H-H -1 6.85828300 -9.97103400 -9.03838400 L

C-CT -1 6.74456100 -9.78090700 -11.17501700 L

H-H1 -1 6.13192700 -9.08627000 -11.74663200 L

C-CT -1 8.19387300 -9.25998400 -11.14188700 L

H-HC -1 8.23975700 -8.35589500 -10.53265000 L

H-HC -1 8.82878300 -10.01082600 -10.66781500 L

C-CT -1 8.76182000 -8.94895000 -12.53957800 L

H-HC -1 9.83586100 -8.77586700 -12.45611000 L

H-HC -1 8.61361200 -9.81015400 -13.18838500 L

C-CT -1 8.11318300 -7.74132000 -13.22255000 L

H-H1 -1 8.45742000 -7.71800800 -14.25918800 L

H-H1 -1 7.03002400 -7.86820500 -13.23477500 L

N-N2 -1 8.48012300 -6.46824600 -12.57547600 L

H-H -1 9.27158900 -6.46281700 -11.94955600 L

C-CM -1 7.95808900 -5.28974100 -12.84824000 L

N-N2 -1 6.88368700 -5.14479000 -13.56450600 L

H-H -1 6.33120900 -5.93510200 -13.83077500 L

H-H -1 6.45926700 -4.21853500 -13.56852500 L

N-N2 -1 8.52171000 -4.20884300 -12.41172200 L

H-H -1 9.30696300 -4.25876800 -11.77286700 L

H-H -1 8.26416400 -3.31706600 -12.83308200 L

C-C -1 6.63026700 -11.13166300 -11.89053200 L

O-O -1 6.11926500 -11.17108400 -13.00687800 L

N-N -1 1.97706200 -3.42550100 -14.05715900 L

H-H -1 0.99808100 -3.26930600 -14.28129000 L

C-CT -1 2.67734200 -2.37729400 -13.30374000 L

H-H1 -1 3.68707300 -2.30202800 -13.69044000 L

C-CT -1 2.01372500 -1.00992900 -13.54917100 L

H-HC -1 0.97744600 -1.04859000 -13.21942000 L

C-CT -1 2.71831500 0.12125600 -12.79745300 L

H-HC -1 3.74922300 0.20890500 -13.13553000 L

H-HC -1 2.20253800 1.06323100 -12.98502700 L

H-HC -1 2.70140600 -0.06582100 -11.72358100 L

C-CT -1 2.04228400 -0.65240400 -15.04641000 L

H-HC -1 1.42466700 -1.34853900 -15.61483500 L

H-HC -1 1.64804100 0.35083000 -15.20059200 L

H-HC -1 3.06479000 -0.69193900 -15.42200700 L

C-C -1 2.76154900 -2.74925000 -11.81679500 L

O-O -1 1.74741200 -2.79089000 -11.12536100 L

N-N -1 3.96403500 -3.02907400 -11.31496800 L

H-H -1 4.76100300 -2.95825600 -11.94441400 L

C-CT -1 4.21506700 -3.43664100 -9.91868200 L

H-H1 -1 3.27908600 -3.77644500 -9.47160700 L

C-CT -1 5.20834000 -4.61819400 -9.85023300 L

H-HC -1 6.16479800 -4.31054100 -10.27593200 L

C-CT -1 5.44090200 -5.07816400 -8.40397000 L

H-HC -1 4.49775800 -5.37881500 -7.94686000 L

H-HC -1 6.12983000 -5.92335500 -8.39031700 L

H-HC -1 5.88167000 -4.27337500 -7.81670700 L

C-CT -1 4.69880200 -5.83432000 -10.63774000 L

H-HC -1 4.58159400 -5.58129800 -11.69035000 L

H-HC -1 5.41264500 -6.65426800 -10.55829800 L

H-HC -1 3.73714800 -6.15744500 -10.24038900 L

C-C -1 4.71287900 -2.23848900 -9.10997500 L

O-O -1 5.79990000 -1.71968200 -9.36936400 L

N-N 0 3.96933200 -1.83098600 -8.07683300 L

H-H 0 3.10558500 -2.31370000 -7.88497400 L

C-CT 0 4.29306400 -0.69499000 -7.21482900 L

H-H1 0 5.06869400 -0.09465500 -7.69208400 L

C-CT 0 3.05099800 0.21375400 -7.03247500 L

H-HC 0 2.29525300 -0.35709700 -6.50249300 L

C-CT 0 3.41138500 1.44147800 -6.16872400 L

H-HC 0 4.17800100 2.03887200 -6.66249200 L

H-HC 0 2.53108700 2.05796400 -5.99181000 L

H-HC 0 3.77865400 1.12653600 -5.19248300 L

C-CT 0 2.44877900 0.62502500 -8.40062000 L

H-HC 0 3.21226000 1.11401200 -9.00250300 L

H-HC 0 2.13128600 -0.27103100 -8.93472400 L

C-CT 0 1.21884100 1.53761600 -8.31676100 L

H-HC 0 1.50171600 2.52899300 -7.96318200 L

H-HC 0 0.77898600 1.63949400 -9.30932000 L

H-HC 0 0.47963200 1.10225000 -7.64389500 L

C-C 0 4.83902200 -1.23322900 -5.88218700 L

O-O 0 4.28570000 -2.17533000 -5.31209600 L

N-N 0 5.92120000 -0.60806200 -5.39342700 L

H-H 0 6.30785000 0.15910600 -5.92427800 L

C-CT 0 6.59632800 -0.92748800 -4.14077300 L

H-H1 0 6.19326300 -1.85290500 -3.72757600 L

C-CT 0 8.09026600 -1.13584700 -4.40648100 L

H-HC 0 8.52897600 -0.23626100 -4.84090800 L

H-HC 0 8.59756300 -1.36746200 -3.47076900 L

H-HC 0 8.22654700 -1.96549800 -5.09636800 L

C-C 0 6.37020300 0.21190800 -3.14718300 L H-H 1448 0.0000

O-O 0 6.85713100 1.31981600 -3.36028300 L

N-N 0 5.63122200 -0.07204900 -2.07780300 H

H-H -1 5.43650300 -1.05165900 -1.93246600 H

C-CT -1 5.33691300 0.87644000 -1.02577600 H

H-H1 -1 5.53352600 1.88788200 -1.37268300 H

C-CT -1 3.83823200 0.79371200 -0.69589400 H

H-HC -1 3.27736600 1.24183200 -1.51689400 H

H-HC -1 3.54217400 -0.25317600 -0.61781300 H

C-CT -1 3.46741300 1.49855600 0.61743200 H

H-HC -1 3.93209300 0.97311600 1.45336400 H

H-HC -1 3.81254400 2.53349600 0.60320700 H

C-CT -1 1.95685700 1.47662500 0.81765700 H

H-H1 -1 1.50866300 2.21393800 0.15226500 H

H-H1 -1 1.56703300 0.49167700 0.55584300 H

N-N2 -1 1.61280700 1.75146500 2.21889800 H

H-H -1 2.17625700 1.32311000 2.95474200 H

C-CA 0 0.45143000 2.19192200 2.61791500 H

N-N2 -1 -0.34379700 2.88264800 1.85392200 H

H-H 0 -0.33095000 2.83848500 0.86295900 H

H-H -1 -1.13670900 3.34663900 2.30108600 H

N-N2 -1 0.20786300 2.30162500 3.90366000 H

H-H 0 0.61314600 1.64406000 4.54285700 H

H-H -1 -0.73978200 2.58158700 4.15338800 H

C-C -1 6.22831500 0.64274700 0.19478300 H

O-O 0 6.54477200 -0.51618100 0.47667600 H

N-N 0 6.55540200 1.76204000 0.88600000 L H-H1 1470 0.0000

H-H 0 6.23775600 2.65375400 0.53388700 L

C-CT 0 7.18057500 1.77295200 2.20084400 L

H-H1 0 7.23488500 0.74170900 2.54138300 L

C-CT 0 8.63427800 2.29198500 2.12424600 L

H-H1 0 9.18007500 1.66693200 1.41711600 L

C-CT 0 8.77945200 3.75064700 1.67564400 L

H-HC 0 8.24673400 4.41491000 2.35600100 L

H-HC 0 9.83503700 4.02030300 1.66292200 L

H-HC 0 8.39267800 3.87131400 0.66892700 L

O-OH 0 9.26105400 2.16253100 3.38389800 L

H-HO 0 9.22135100 1.24118800 3.65064800 L

C-C 0 6.28927600 2.53746400 3.19265800 L

O-O 0 5.60660300 3.49690300 2.83232700 L

N-N 0 6.31284800 2.08042500 4.45021300 L

H-H 0 6.90748200 1.28745700 4.64477100 L

C-CT 0 5.56210600 2.59766200 5.59403700 L

H-H1 0 4.90411700 3.40585300 5.27480000 L

C-CT 0 4.70103100 1.46267500 6.17588800 L

H-HC 0 5.29417300 0.55092100 6.26043400 L

H-HC 0 4.37193400 1.72659300 7.18087000 L

C-C 0 3.46083500 1.19611600 5.33194900 L

O-O2 0 2.38676300 1.67306100 5.75568300 L

O-O2 0 3.60211200 0.51542400 4.29333000 L

C-C 0 6.50147300 3.15034400 6.67483500 L

O-O 0 6.02860500 3.59704100 7.72018500 L

N-N 0 7.82041000 3.10433800 6.43497000 L

H-H 0 8.13312500 2.75779100 5.53930600 L

C-CT 0 8.85074300 3.29544000 7.43885200 L

H-H1 0 8.59750000 2.65302600 8.27989500 L

C-CT 0 10.18598100 2.78521600 6.88491400 L

H-HC 0 10.52672000 3.41439200 6.06584700 L

H-HC 0 10.93348000 2.78522100 7.67795400 L

H-HC 0 10.06823400 1.76372300 6.52046100 L

C-C 0 8.95884100 4.72509000 7.99228200 L

O-O 0 9.70074000 4.89558000 8.95543100 L

N-N -1 8.22915200 5.73003900 7.46183400 L

H-H -1 7.67611100 5.55873900 6.63513300 L

C-CT -1 8.21305700 7.07820300 8.06389600 L

H-H1 -1 9.24272100 7.39657700 8.19264800 L

C-CT -1 7.53129900 8.07206300 7.10751900 L

H-HC -1 7.69936000 7.70525700 6.09506000 L

H-HC -1 6.45520200 8.08377400 7.28574700 L

C-CT -1 8.09232300 9.50624900 7.13214200 L

H-HC -1 9.17980400 9.46200000 7.03389200 L

H-HC -1 7.71334400 10.02544700 6.25126500 L

C-C -1 7.70847100 10.35263700 8.34999700 L

O-O2 -1 6.52034200 10.74808100 8.46075100 L

O-O2 -1 8.61766000 10.70530000 9.14082400 L

C-C -1 7.55706300 7.07468000 9.45671400 L

O-O -1 7.94799400 7.85203100 10.32115700 L

N-N -1 6.63233700 6.14109000 9.72255600 L

H-H -1 6.37416200 5.52346400 8.96326400 L

C-CT -1 5.94801800 5.99903200 11.01863400 L

H-H1 -1 6.45105800 6.62518900 11.75430600 L

C-CT -1 4.52518100 6.55023400 10.86443300 L

H-HC -1 3.98252200 6.00401200 10.09532500 L

H-HC -1 3.99081100 6.46998300 11.81231900 L

H-HC -1 4.57147800 7.60357900 10.58114700 L

C-C -1 5.97564200 4.57058700 11.61175400 L

O-O -1 5.51854700 4.35424100 12.73595000 L

N-N -1 6.51606300 3.57517200 10.90081200 L

H-H -1 6.89723900 3.77722200 9.98688600 L

C-CT -1 6.57724600 2.19265300 11.38313300 L

H-H1 -1 5.66693300 1.98399800 11.94261500 L

C-CT -1 6.57833800 1.25232800 10.17517800 L

H-HC -1 7.52758300 1.33066600 9.64811000 L

H-HC -1 6.44389700 0.22757400 10.51937600 L

H-HC -1 5.76845800 1.50240800 9.48966200 L

C-C -1 7.75387500 1.96196700 12.35762400 L

O-O -1 8.82433600 1.49728500 11.97749500 L

N-N -1 7.55031800 2.28901100 13.63851900 L

H-H -1 6.67932800 2.76416100 13.83766000 L

C-CT -1 8.57449400 2.20767000 14.71332300 L

H-H1 -1 9.46853800 2.71492500 14.34869300 L

C-CT -1 8.12129600 2.95021800 15.98747600 L

H-H1 -1 8.95914400 2.98128300 16.68639100 L

C-CT -1 7.67034300 4.38774100 15.73909100 L

H-HC -1 6.76568200 4.42169300 15.13254000 L

H-HC -1 7.47842600 4.87739300 16.69378300 L

H-HC -1 8.46472700 4.92910300 15.23142900 L

O-OH -1 7.04298700 2.27921300 16.61385600 L

H-HO -1 6.31676400 2.27793800 15.98192800 L

C-C -1 9.03719200 0.79220900 15.10713400 L

O-O -1 9.80012200 0.64418200 16.06371100 L

N-N -1 8.57532500 -0.25956400 14.42211900 L

H-H -1 8.06351500 -0.07205000 13.56935800 L

C-CT -1 8.79112300 -1.67151000 14.76170500 L

H-H1 -1 9.60158100 -1.75675700 15.48322800 L

C-CT -1 7.49475600 -2.23917100 15.37152000 L

H-HC -1 6.65537400 -1.87207600 14.77690600 L

H-HC -1 7.50546700 -3.32649200 15.28872700 L

C-CT -1 7.26028800 -1.88095500 16.84725500 L

H-HC -1 7.45108700 -0.82165500 17.00773300 L

C-CT -1 5.81277900 -2.17801000 17.23923400 L

H-HC -1 5.59179600 -3.23467100 17.08637700 L

H-HC -1 5.64950400 -1.93093100 18.28797400 L

H-HC -1 5.13648400 -1.58064600 16.62711500 L

C-CT -1 8.16502600 -2.70963600 17.76086100 L

H-HC -1 9.21083200 -2.48403500 17.56523400 L

H-HC -1 7.94170400 -2.49240700 18.80099000 L

H-HC -1 7.99182000 -3.76881500 17.58446300 L

C-C -1 9.18490900 -2.49165800 13.52739200 L

O-O -1 8.64660400 -2.27456400 12.44502200 L

N-N -1 10.06282300 -3.48035400 13.70412700 L

H-H -1 10.48583100 -3.58132600 14.62241600 L

C-CT -1 10.46775300 -4.45154700 12.67229600 L

H-H1 -1 9.64518400 -4.53614400 11.96215700 L

C-CT -1 11.69715400 -3.92200300 11.89233900 L

H-HC -1 11.43129300 -2.92899800 11.52738600 L

C-CT -1 12.93348500 -3.75938300 12.79122100 L

H-HC -1 13.33294100 -4.73602200 13.06549600 L

H-HC -1 13.69976800 -3.19999900 12.25735500 L

H-HC -1 12.68507800 -3.20322600 13.69492400 L

C-CT -1 12.02314200 -4.79120900 10.65634500 L

H-HC -1 12.42235900 -5.75394000 10.97524700 L

H-HC -1 11.10623100 -4.96881100 10.09606600 L

C-CT -1 13.03216700 -4.14989200 9.69602400 L

H-HC -1 14.01061800 -4.06812900 10.16843000 L

H-HC -1 13.13118400 -4.77688400 8.81148600 L

H-HC -1 12.68597300 -3.16047100 9.39364900 L

C-C -1 10.67579900 -5.85106400 13.27764900 L

O-O -1 10.99535100 -5.99350000 14.46086400 L

N-N -1 10.46161300 -6.90342400 12.48411900 L

H-H -1 10.12136700 -6.72950300 11.54396800 L

C-CT -1 10.46384000 -8.30341400 12.95944700 L

H-H1 -1 10.06496500 -8.32024200 13.97532800 L

C-CT -1 9.53993500 -9.17567900 12.10133200 L

H-H1 -1 9.57808000 -10.20021400 12.47130500 L

C-CT -1 8.08828000 -8.72115500 12.14204800 L

H-HC -1 7.97323900 -7.73809400 11.68816600 L

H-HC -1 7.47588500 -9.44268400 11.60677400 L

H-HC -1 7.75495600 -8.67467600 13.17884400 L

O-OH -1 9.95491800 -9.16946200 10.75556900 L

H-HO -1 9.24087000 -9.55486300 10.24244100 L

C-C -1 11.82648300 -8.99632100 13.03612000 L

O-O -1 11.91536100 -10.04485600 13.67308200 L

N-N -1 18.59341200 -2.51442300 6.88804200 L

H-H -1 18.88294700 -3.48013500 6.79665700 L

C-CT -1 17.68132100 -2.18031000 7.98945400 L

H-H1 -1 17.22068900 -1.21287600 7.79731200 L

C-CT -1 16.55304200 -3.22209600 8.03298500 L

H-HC -1 16.98135000 -4.22557400 8.08806800 L

H-HC -1 15.97106600 -3.06576200 8.94238700 L

C-C -1 15.60228600 -3.11111500 6.83873200 L

O-O2 -1 14.95465600 -2.05755800 6.67344900 L

O-O2 -1 15.37884400 -4.10468900 6.11048400 L

C-C -1 18.39618100 -2.06900400 9.35291800 L

O-O -1 18.00302700 -1.25397200 10.19048100 L

N-N -1 12.09581400 5.21375700 12.77210000 L

H-H -1 11.68994100 4.98219500 13.66824500 L

C-CT -1 11.19608900 5.66601600 11.71530400 L

H-H1 -1 11.24259100 4.94121900 10.90125200 L

C-CT -1 9.76805600 5.68319500 12.28817800 L

H-HC -1 9.06479700 5.80869800 11.46866100 L

H-HC -1 9.54905900 4.72901800 12.76591200 L

C-C -1 9.56853900 6.80240100 13.30178800 L

O-O -1 10.10434700 6.77479400 14.40050000 L

N-N -1 8.84370500 7.83973100 12.96652200 L

H-H -1 8.74225500 8.57891100 13.63460400 L

H-H -1 8.47878600 7.93127300 12.01807300 L

C-C -1 11.58962300 7.04927900 11.15245800 L

O-O -1 12.04989800 7.91940400 11.89933500 L

N-N -1 11.36120000 7.26138300 9.85673000 L

H-H -1 10.97652000 6.49992600 9.30495700 L

C-CT -1 11.58928200 8.53979400 9.17670100 L

H-H1 -1 10.71669300 9.18077700 9.31102800 L

H-H1 -1 12.45016500 9.04124500 9.62126700 L

C-C -1 11.84777600 8.37663800 7.67712700 L

O-O -1 11.64809300 7.30252000 7.11339300 L

N-N -1 12.33032000 9.43736900 7.02318200 L

H-H -1 12.43474700 10.30297400 7.53118400 L

C-CT -1 12.62177300 9.43149200 5.57762600 L

H-H1 -1 11.80786400 8.89687700 5.08861400 L

C-CT -1 12.60239500 10.87961600 5.03239900 L

H-HC -1 11.65944600 11.32792500 5.34898200 L

C-CT -1 13.74708100 11.73448100 5.61094500 L

H-HC -1 14.71130900 11.38754000 5.23750000 L

H-HC -1 13.62026000 12.77861400 5.32283400 L

H-HC -1 13.74788500 11.68880400 6.69812400 L

C-CT -1 12.62630500 10.89139800 3.48899800 L

H-HC -1 13.62404800 10.63422700 3.14108900 L

H-HC -1 11.93898000 10.13494800 3.11153600 L

C-CT -1 12.22593200 12.23739100 2.87477900 L

H-HC -1 12.92097700 13.01902800 3.17541500 L

H-HC -1 12.24661700 12.15322700 1.78855800 L

H-HC -1 11.21688200 12.50550100 3.18986300 L

C-C -1 13.90479800 8.66333300 5.20545500 L

O-O -1 13.99024300 8.11155100 4.11303500 L

N-NB -1 15.20512100 5.61687200 6.43413800 L

C-CT -1 14.74687100 5.95453000 7.77812300 L

H-H1 -1 13.93898600 6.67713800 7.72315400 L

H-H1 -1 15.55285400 6.34196700 8.39537400 L

C-CT -1 14.23298100 4.66124100 8.40525800 L

H-HC -1 13.39295700 4.84656700 9.07413500 L

H-HC -1 15.03949200 4.15837200 8.93455800 L

C-CT -1 13.81166100 3.82433300 7.20697900 L

H-HC -1 12.78490500 4.08535600 6.94681200 L

H-HC -1 13.88996500 2.75573500 7.41271000 L

C-CT -1 14.77183000 4.25635000 6.09258000 L

H-H1 -1 15.64142500 3.59901100 6.09168700 L

C-C -1 14.07093000 4.15958700 4.73265800 L

O-O -1 14.37904900 3.26689100 3.94160600 L

N-N -1 13.15538300 5.08583700 4.42849700 L

H-H -1 12.91284100 5.78243900 5.11834500 L

C-CT -1 12.39655200 5.06296800 3.17728900 L

H-H1 -1 11.92782600 4.08284600 3.07541900 L

C-CT -1 11.28646200 6.11870400 3.22199400 L

H-H1 -1 11.71485800 7.10809800 3.38715400 L

H-H1 -1 10.75963900 6.12151400 2.26561700 L

S-SH -1 10.10224500 5.72815700 4.53904600 L

H-HS -1 9.23207600 6.70745700 4.26816400 L

C-C -1 13.29307900 5.26250600 1.94593500 L

O-O -1 13.08202100 4.59665400 0.93697100 L

N-N -1 14.31994800 6.11764500 2.02363900 L

H-H -1 14.41365900 6.66525300 2.87595600 L

C-CT -1 15.31753600 6.30697000 0.95620500 L

H-H1 -1 14.79571200 6.50850500 0.01956200 L

C-CT -1 16.21623900 7.52586100 1.28003600 L

H-HC -1 16.56996900 7.42421200 2.30787400 L

C-CT -1 17.45027100 7.59266400 0.35429600 L

H-HC -1 17.13588100 7.66393600 -0.68777400 L

H-HC -1 18.06404200 8.45534300 0.60468500 L

H-HC -1 18.07829000 6.71228800 0.48428600 L

C-CT -1 15.40917000 8.84140400 1.15783500 L

H-HC -1 15.29221600 9.09830000 0.10384000 L

H-HC -1 14.40962600 8.70613000 1.56839800 L

C-CT -1 16.05160400 10.02232900 1.90230300 L

H-HC -1 17.07151300 10.19188800 1.56683100 L

H-HC -1 15.47059500 10.92494800 1.71250400 L

H-HC -1 16.05996000 9.82150700 2.97340100 L

C-C -1 16.13795700 5.02351400 0.74329400 L

O-O -1 16.32167400 4.59572000 -0.39862000 L

N-N -1 16.59729900 4.37982300 1.82339000 L

H-H -1 16.41751000 4.77207500 2.74174300 L

C-CT -1 17.34951000 3.12473800 1.73939600 L

H-H1 -1 18.21561100 3.27853100 1.09414300 L

C-CT -1 17.85355100 2.76554800 3.14416800 L

H-HC -1 17.01327600 2.59529900 3.81814800 L

H-HC -1 18.45811300 1.85832300 3.09659200 L

H-HC -1 18.46471100 3.57872000 3.53783000 L

C-C -1 16.50667800 1.99383100 1.11612200 L

O-O -1 16.96050300 1.30563300 0.19620200 L

N-N 0 15.25425900 1.83499800 1.56833300 L

H-H 0 14.93473700 2.44439100 2.30516700 L

C-CT 0 14.27323000 0.89230500 1.03914800 L

H-H1 0 14.71673600 -0.10428500 1.07083200 L

C-CT 0 13.01761200 0.87672300 1.92567000 L

H-HC 0 12.70358500 1.89793400 2.14601400 L

H-HC 0 12.21293500 0.38617100 1.38012200 L

C-CT 0 13.25372000 0.09953800 3.23196300 L

H-HC 0 13.56377100 -0.91586100 2.98681500 L

H-HC 0 14.03784500 0.57843000 3.81873600 L

C-CT 0 11.96375000 0.04065000 4.05493500 L

H-H1 0 11.66787000 1.05618400 4.31898500 L

H-H1 0 11.17803100 -0.39820700 3.44223400 L

N-N2 0 12.11365800 -0.73772500 5.29723300 L

H-H 0 12.30320600 -0.20015300 6.13097600 L

C-CA 0 11.95488700 -2.06882900 5.44198800 L

N-N2 0 11.72421300 -2.87745900 4.39990300 L

H-H 0 11.67067500 -2.49297100 3.46813500 L

H-H 0 11.61126500 -3.87066800 4.54519200 L

N-N2 0 12.02954600 -2.61216300 6.66276500 L

H-H 0 12.20237800 -2.02589200 7.46682800 L

H-H 0 11.91366200 -3.60864200 6.78019100 L

C-C 0 13.92147800 1.18743000 -0.42665600 L

O-O 0 13.86178500 0.24422100 -1.20912300 L

N-N -1 13.69971600 2.45870700 -0.80595200 L

H-H -1 13.67875500 3.16990500 -0.08444000 L

C-CT -1 13.45181000 2.86754600 -2.18872400 L

H-H1 -1 12.56326300 2.34547700 -2.54517800 L

C-CT -1 13.17247300 4.37229700 -2.22841900 L

H-HC -1 14.01235900 4.92763000 -1.81167000 L

H-HC -1 13.00822900 4.69214300 -3.25793800 L

H-HC -1 12.27605900 4.58679900 -1.64707300 L

C-C -1 14.60789400 2.48152000 -3.12567800 L

O-O -1 14.35908300 1.87830700 -4.16991500 L

N-N -1 15.86974000 2.73157500 -2.74338800 L

H-H -1 16.02371300 3.23368100 -1.87231100 L

C-CT -1 17.03322200 2.27114500 -3.52764200 L

H-H1 -1 16.93920200 2.64423700 -4.54837000 L

C-CT -1 18.34430800 2.80406700 -2.93124000 L

H-HC -1 18.37783000 2.57365700 -1.86465800 L

H-HC -1 19.17489100 2.28698100 -3.41605000 L

C-CT -1 18.53738100 4.31465000 -3.14009500 L

H-HC -1 18.46007200 4.55287100 -4.20224300 L

H-HC -1 17.76817000 4.86421800 -2.59720400 L

C-CT -1 19.92384100 4.72556200 -2.62860300 L

H-HC -1 20.02938400 4.39193100 -1.59427400 L

H-HC -1 20.69039600 4.24137700 -3.23667700 L

C-CT -1 20.11824100 6.24318400 -2.67821700 L

H-HP -1 19.97648100 6.60274300 -3.70224400 L

H-HP -1 19.35275300 6.71468400 -2.05417500 L

N-N3 -1 21.46431600 6.62084500 -2.18918400 L

H-H -1 22.18805200 6.39184000 -2.87308200 L

H-H -1 21.51538100 7.61252200 -2.00600700 L

H-H -1 21.70635200 6.08940400 -1.35828700 L

C-C -1 17.09178400 0.74339900 -3.65627600 L

O-O -1 17.47946300 0.25090900 -4.70859200 L

N-N -1 16.67256300 -0.00570900 -2.63375000 L

H-H -1 16.39656000 0.45712400 -1.77815200 L

C-CT -1 16.62321700 -1.46966700 -2.68171400 L

H-H1 -1 17.55346200 -1.82842900 -3.12477500 L

C-CT -1 16.55616200 -1.98968800 -1.24182800 L

H-HC -1 15.60434600 -1.71308700 -0.78856100 L

H-HC -1 16.64581300 -3.07574700 -1.24598100 L

H-HC -1 17.37111300 -1.56539900 -0.65483600 L

C-C -1 15.47257200 -2.03156700 -3.54700400 L

O-O -1 15.59679200 -3.13341400 -4.07907400 L

N-N 0 14.37171200 -1.27932700 -3.71618000 L

H-H 0 14.33302100 -0.37561500 -3.26672700 L

C-CT 0 13.19687000 -1.66807100 -4.50372700 L

H-H1 0 13.11478300 -2.75531500 -4.50703400 L

C-CT 0 11.91718600 -1.09777700 -3.87268100 L

H-HC 0 11.96501000 -0.00884600 -3.93371700 L

H-HC 0 11.06883200 -1.42093900 -4.47661200 L

C-CA 0 11.62522200 -1.48084100 -2.43003000 L

C-CA 0 12.20354400 -2.62220100 -1.83181800 L

H-HA 0 12.85549200 -3.28182600 -2.38353400 L

C-CA 0 11.95526900 -2.91739700 -0.48326500 L

H-HA 0 12.41617300 -3.78324600 -0.03106200 L

C-C 0 11.09368000 -2.08951300 0.27019900 L

O-OH 0 10.83629300 -2.38788300 1.57146600 L

H-HO 0 11.29658500 -3.17407100 1.87418200 L

C-CA 0 10.48387600 -0.96638500 -0.33344300 L

H-HA 0 9.81590900 -0.33632000 0.23562400 L

C-CA 0 10.76028100 -0.66260400 -1.67601100 L

H-HA 0 10.30462600 0.20787000 -2.12578400 L

C-C 0 13.28398900 -1.20781500 -5.96343300 L

O-O 0 12.65053200 -1.82302900 -6.81783300 L

N-N -1 14.03867100 -0.13283500 -6.23837600 L

H-H -1 14.46112000 0.34275900 -5.44851000 L

C-CT -1 14.21058500 0.48858500 -7.55369600 L

H-H1 -1 13.24346200 0.90105600 -7.84272200 L

C-CT -1 15.19202800 1.66296000 -7.42004500 L

H-HC -1 16.16428700 1.31087200 -7.07595200 L

H-HC -1 15.31875500 2.14923800 -8.38829800 L

H-HC -1 14.81146800 2.39407800 -6.70877400 L

C-C -1 14.63142800 -0.46235500 -8.70063500 L

O-O -1 14.13985700 -0.24623200 -9.80963100 L

N-N -1 15.45137800 -1.52018800 -8.49910300 L

C-CT -1 16.32646800 -1.75078100 -7.35734500 L

H-H1 -1 15.83875400 -2.42771700 -6.65366300 L

H-H1 -1 16.60867700 -0.82681600 -6.85776900 L

C-CT -1 17.56866100 -2.41758000 -7.93422100 L

H-HC -1 18.09845600 -3.00580000 -7.18481300 L

H-HC -1 18.22351000 -1.66258400 -8.37303000 L

C-CT -1 16.97334900 -3.29310000 -9.03349400 L

H-HC -1 16.61599000 -4.22519800 -8.59228700 L

H-HC -1 17.70156700 -3.50246000 -9.81825500 L

C-CT -1 15.78997100 -2.47152600 -9.56696400 L

H-H1 -1 16.11832800 -1.90762500 -10.44063800 L

C-C -1 14.64093300 -3.40153800 -9.99342900 L

O-O -1 14.75138900 -4.06734200 -11.02340600 L

N-N -1 13.55831600 -3.48429000 -9.20939600 L

H-H -1 13.52947900 -2.90006400 -8.38324200 L

C-CT -1 12.52066600 -4.51633400 -9.34936000 L

H-H1 -1 12.77318900 -5.17699500 -10.18009900 L

C-CT -1 12.50363200 -5.36730900 -8.06809400 L

H-HC -1 12.09803500 -4.76422700 -7.25469800 L

H-HC -1 11.82680600 -6.21070000 -8.21691300 L

C-CA -1 13.86019600 -5.90696000 -7.64760600 L

C-CA -1 14.50461500 -6.88483800 -8.42828200 L

H-HA -1 14.03140300 -7.26261700 -9.32343100 L

C-CA -1 15.77578700 -7.35935500 -8.05781400 L

H-HA -1 16.27248300 -8.10169000 -8.66583500 L

C-CA -1 16.40697600 -6.85767800 -6.90479900 L

H-HA -1 17.38881100 -7.21449700 -6.62559700 L

C-CA -1 15.76671100 -5.88031900 -6.12481200 L

H-HA -1 16.25237200 -5.48052500 -5.24519700 L

C-CA -1 14.49633900 -5.40469500 -6.49564300 L

H-HA -1 14.02177200 -4.64217500 -5.89567700 L

C-C -1 11.12027000 -3.95475500 -9.63715700 L

O-O -1 10.34434400 -4.59264000 -10.35463800 L

N-N -1 10.78938500 -2.78092000 -9.09033300 L

H-H -1 11.48461100 -2.31253300 -8.52249700 L

C-CT -1 9.48005500 -2.13276500 -9.20805700 L

H-H1 -1 8.70929600 -2.90367400 -9.24643700 L

C-CT -1 9.25570500 -1.31509800 -7.93011400 L

H-HC -1 9.96409300 -0.48502200 -7.88997900 L

H-HC -1 8.24011800 -0.91923100 -7.91140300 L

H-HC -1 9.40439700 -1.95289200 -7.05940600 L

C-C -1 9.32780800 -1.26820800 -10.47949400 L

O-O -1 10.29774400 -0.99597300 -11.18440300 L

N-N -1 8.09915500 -0.82219200 -10.75514300 L

H-H -1 7.33866100 -1.13510000 -10.15878500 L

C-CT -1 7.76732300 0.13778900 -11.82293600 L

H-H1 -1 8.61035200 0.24171000 -12.50870100 L

C-CT -1 6.56076300 -0.37910500 -12.61085700 L

H-HC -1 5.71595500 -0.49205200 -11.92901100 L

H-HC -1 6.28248000 0.35495400 -13.36889100 L

C-C -1 6.82682500 -1.70536000 -13.30283900 L

O-O2 -1 7.88999200 -1.92946900 -13.91222900 L

O-O2 -1 5.95214200 -2.59220600 -13.27963200 L

C-C -1 7.43469100 1.54259400 -11.29951400 L

O-O -1 7.54596900 2.50775100 -12.04707400 L

N-N 0 6.99439400 1.64972100 -10.03505300 L

H-H 0 6.88026200 0.79870400 -9.50311000 L

C-CT 0 6.86197400 2.89421300 -9.27746300 L

H-H1 0 7.54986400 3.62399900 -9.69879200 L

C-CT 0 5.43567400 3.48786700 -9.32381500 L

H-HC 0 4.72928600 2.77334200 -8.90596300 L

H-HC 0 5.43737900 4.35369900 -8.65984200 L

C-CT 0 4.94628400 3.95970100 -10.71226400 L

H-HC 0 5.80546400 4.25036600 -11.30981000 L

C-CT 0 4.16354400 2.86709500 -11.45654300 L

H-HC 0 3.24117400 2.63739900 -10.92212300 L

H-HC 0 3.91454900 3.21181700 -12.46074900 L

H-HC 0 4.75488900 1.95888900 -11.54000500 L

C-CT 0 4.04567600 5.19746800 -10.59475700 L

H-HC 0 4.59893700 6.01468500 -10.13195300 L

H-HC 0 3.72487200 5.51928500 -11.58626500 L

H-HC 0 3.16720900 4.96267800 -9.99299500 L

C-C 0 7.26873400 2.62934600 -7.82051100 L

O-O 0 7.25634100 1.48361000 -7.36682000 L

N-N 0 7.60788800 3.69827400 -7.08245800 L

H-H 0 7.59193600 4.61353300 -7.50946400 L

C-CT 0 8.00306400 3.62776600 -5.67797100 L

H-H1 0 7.72085500 2.64656700 -5.29948500 L

C-CT 0 9.52928200 3.78791800 -5.46560900 L

H-HC 0 9.76988900 4.85046500 -5.50645200 L

C-CT 0 9.87402300 3.26419200 -4.05550600 L

H-HC 0 9.77070300 2.17868800 -4.02199300 L

H-HC 0 10.89287600 3.53662000 -3.79214900 L

H-HC 0 9.21598600 3.69922000 -3.30611300 L

C-CT 0 10.39086800 3.06537700 -6.52793600 L

H-HC 0 10.19741000 1.99194800 -6.49558600 L

H-HC 0 10.12843400 3.43191000 -7.52017800 L

C-CT 0 11.89195900 3.31403000 -6.34992100 L

H-HC 0 12.25477100 2.81558200 -5.45339400 L

H-HC 0 12.42276100 2.90964400 -7.20690000 L

H-HC 0 12.09060600 4.38435700 -6.28480100 L

C-C 0 7.21774300 4.68781900 -4.90269000 L

O-O 0 7.22951500 5.86720200 -5.24849300 L

N-N 0 6.55360800 4.24072800 -3.83488300 L

H-H 0 6.63935500 3.26106600 -3.60986500 L

C-CT 0 5.58945400 4.97973500 -3.03785600 L

H-H1 0 5.54163700 6.00892100 -3.38626700 L

C-CT 0 4.22599500 4.31080400 -3.26923800 L

H-HC 0 3.99647300 4.36227100 -4.33314800 L

H-HC 0 4.32466300 3.25332200 -3.01897200 L

C-C* 0 3.02954400 4.82522600 -2.53057000 L

C-CW 0 2.85390900 6.06202400 -2.01389900 L

H-H4 0 3.55923600 6.87597200 -2.06533900 L

N-NA 0 1.64488300 6.12432300 -1.36143000 L

H-H 0 1.32796300 6.95536600 -0.88286700 L

C-CN 0 0.94354300 4.94198900 -1.47098500 L

C-CA 0 -0.31854100 4.51763700 -1.02248400 L

H-HA 0 -0.94335600 5.16014400 -0.42407900 L

C-CA 0 -0.77319400 3.23786800 -1.37124700 L

H-HA 0 -1.73991700 2.89566600 -1.03618100 L

C-CA 0 0.03489600 2.40359500 -2.15876400 L

H-HA 0 -0.30945000 1.42216200 -2.42277100 L

C-CA 0 1.29903200 2.83107400 -2.59615200 L

H-HA 0 1.90040500 2.17168200 -3.20442500 L

C-CB 0 1.79323400 4.10600000 -2.25094000 L

C-C 0 6.03248700 4.97795400 -1.56924300 L

O-O 0 6.68027700 4.03796300 -1.11586400 L

N-N 0 5.69807500 6.05337800 -0.84337500 L

H-H 0 5.16776600 6.78309200 -1.29726900 L

C-CT 0 6.06135500 6.30261700 0.54612600 L

H-H1 0 6.28546000 5.35607600 1.03839700 L

C-CT 0 7.32384700 7.18107300 0.57279500 L

H-HC 0 8.15284100 6.58548800 0.20242200 L

H-HC 0 7.18144000 8.03732900 -0.08887900 L

C-CT 0 7.70365700 7.70391500 1.96078700 L

H-H1 0 6.87273300 8.29116100 2.34656200 L

H-H1 0 7.87785300 6.86035100 2.62876500 L

S-S 0 9.16188500 8.77873000 1.96796700 L

C-CT 0 9.15971400 9.25875000 3.71285100 L

H-H1 0 9.55439700 8.44942900 4.32235100 L

H-H1 0 9.77316400 10.14750100 3.85134900 L

H-H1 0 8.14137400 9.47222000 4.02574200 L

C-C 0 4.89184800 6.97320200 1.27233000 L

O-O 0 4.30662700 7.93517200 0.77219100 L

N-N 0 4.60040600 6.47528100 2.48170000 L

H-H 0 5.12250600 5.67545700 2.80985500 L

C-CT 0 3.70759900 7.10483000 3.44679300 L

H-H1 0 2.90357600 7.61794000 2.91748100 L

C-CT 0 3.10142300 6.04764200 4.38191900 L

H-HC 0 3.90462500 5.50288000 4.87988900 L

H-HC 0 2.51198900 6.55186900 5.14903800 L

C-CT 0 2.19387500 5.04643900 3.66029500 L

H-HC 0 2.78107200 4.46600600 2.94707900 L

H-HC 0 1.80281700 4.35841200 4.40689600 L

C-C 0 1.02820700 5.72686400 2.93645400 L

O-O2 0 1.11550400 5.83507400 1.69465300 L

O-O2 0 0.07159500 6.13356200 3.63095300 L

C-C 0 4.47826200 8.12815800 4.28120800 L

O-O 0 5.66742600 7.95999600 4.54649800 L

N-N -1 3.76665100 9.17370100 4.71491900 L

H-H -1 2.79208600 9.22696700 4.44262100 L

C-CT -1 4.28338800 10.27976300 5.54597300 L

H-H1 -1 5.19948200 9.95586500 6.03945000 L

C-CT -1 4.62637400 11.51571900 4.68959000 L

H-H1 -1 4.98841100 12.29494500 5.36125700 L

C-CT -1 5.70510900 11.28588500 3.63469000 L

H-HC -1 5.39850800 10.50794000 2.93491100 L

H-HC -1 5.88023900 12.21020400 3.08488500 L

H-HC -1 6.62582700 10.99155100 4.12942200 L

O-OH -1 3.49564700 12.01820200 4.01745700 L

H-HO -1 3.51364300 12.98533700 4.15479500 L

C-C -1 3.30585400 10.70341900 6.64691600 L

O-O -1 2.09456600 10.53002200 6.52440400 L

N-N -1 3.81581600 11.31139700 7.72494900 L

H-H -1 4.82644100 11.28168000 7.87051200 L

C-CT -1 2.98226200 11.95551100 8.75290400 L

H-H1 -1 2.20790000 11.26031800 9.07505100 L

H-H1 -1 3.61277000 12.19113900 9.61061700 L

C-C -1 2.29959300 13.25802700 8.29805200 L

O-O -1 1.25233200 13.62068000 8.83374700 L

N-N -1 2.85815300 13.94552500 7.29549400 L

H-H -1 3.72095900 13.59840200 6.90726500 L

C-CT -1 2.31747100 15.17815800 6.68709900 L

H-H1 -1 1.23934000 15.18915800 6.84492200 L

C-CT -1 2.90294300 16.44980400 7.33778400 L

H-H1 -1 2.43933800 17.31862800 6.87144900 L

C-CT -1 2.67306800 16.55566900 8.84404800 L

H-HC -1 3.22685700 15.77693600 9.36814500 L

H-HC -1 3.00823000 17.53004100 9.19839000 L

H-HC -1 1.61167900 16.44926300 9.06055200 L

O-OH -1 4.29161200 16.52828500 7.12124900 L

H-HO -1 4.60563500 17.33496700 7.53969900 L

C-C -1 2.58514200 15.22663900 5.17051900 L

O-O -1 3.54879900 14.60876800 4.70178400 L

N-N -1 1.77887900 15.95798000 4.37516500 L

C-CT -1 0.51892000 16.57326800 4.77510900 L

H-H1 -1 0.67768400 17.33975600 5.53265500 L

H-H1 -1 -0.16038600 15.80476800 5.14640100 L

C-CT -1 -0.07565500 17.20717100 3.51867800 L

H-HC -1 0.24146700 18.24857200 3.44871500 L

H-HC -1 -1.16362700 17.13284800 3.50750500 L

C-CT -1 0.56120900 16.39603200 2.39313000 L

H-HC -1 0.56440100 16.93955400 1.45117100 L

H-HC -1 0.02669400 15.45053400 2.28220200 L

C-CT -1 1.97004500 16.11479600 2.92957700 L

H-H1 -1 2.32002100 15.17571900 2.49936200 L

C-C -1 3.02003700 17.19583600 2.59387600 L

O-O -1 2.73154000 18.19556900 1.93537900 L

N-N 0 13.67613300 11.16655400 -0.93140300 L

H-H 0 14.14323100 10.96000800 -0.05992100 L

C-CT 0 12.92498800 10.10202600 -1.56911600 L

H-H1 0 13.51518000 9.18673800 -1.52728900 L

C-CT 0 11.61121500 9.87331900 -0.81072900 L

H-HC 0 11.84454800 9.65342700 0.23260600 L

H-HC 0 11.02431200 10.79333200 -0.82803800 L

C-CA 0 10.76691800 8.74012200 -1.36488400 L

C-CA 0 10.96506200 7.42168500 -0.91005100 L

H-HA 0 11.71192200 7.20821000 -0.15878200 L

C-CA 0 10.17797600 6.37699800 -1.42439400 L

H-HA 0 10.29969700 5.37053300 -1.05415400 L

C-CA 0 9.20265300 6.64396800 -2.39894600 L

H-HA 0 8.59372200 5.84310800 -2.78049300 L

C-CA 0 9.00456700 7.95491100 -2.85944600 L

H-HA 0 8.25239100 8.15480100 -3.60798800 L

C-CA 0 9.78356400 9.00238300 -2.33994600 L

H-HA 0 9.62733500 10.00879200 -2.69814800 L

C-C 0 12.68547300 10.47280600 -3.03031500 L

O-O 0 12.63082000 9.60589500 -3.90077400 L

N-N -1 17.10470600 9.55240500 -3.99218100 L

H-H -1 16.35346400 10.23273800 -4.02652400 L

C-CT -1 17.00862700 8.36505400 -4.86011600 L

H-H1 -1 17.64209800 7.57758100 -4.45411900 L

C-CT -1 15.56673300 7.81457400 -4.88722200 L

H-HC -1 14.88753400 8.60514500 -5.20560500 L

C-CT -1 15.41395600 6.62678700 -5.84834100 L

H-HC -1 16.11971400 5.83755400 -5.58847200 L

H-HC -1 14.39847500 6.23316200 -5.78725600 L

H-HC -1 15.59002700 6.94516300 -6.87584700 L

C-CT -1 15.14950300 7.32133300 -3.49296300 L

H-HC -1 15.22675300 8.12941000 -2.76613400 L

H-HC -1 14.10791600 6.99981500 -3.51720700 L

H-HC -1 15.78139400 6.49209300 -3.17376300 L

C-C -1 17.54265600 8.67177700 -6.26309800 L

O-O -1 18.35901400 7.90989400 -6.77982200 L

N-N -1 13.80310000 6.02651700 -12.79525100 L

H-H -1 14.71861700 6.08097700 -12.37683500 L

C-CT -1 12.74876300 5.28439000 -12.07973700 L

H-H1 -1 12.30489300 4.58835000 -12.79358400 L

C-CT -1 13.41000500 4.44942300 -10.96203500 L

H-HC -1 14.16985300 3.81113400 -11.41626800 L

H-HC -1 13.91672700 5.12918800 -10.27495700 L

C-CT -1 12.47127800 3.55051200 -10.13692400 L

H-HC -1 13.07287200 3.00526100 -9.40935000 L

H-HC -1 11.76967600 4.17143400 -9.57982900 L

C-C -1 11.67346200 2.54587400 -10.96682700 L

O-O -1 10.70157100 2.89903800 -11.60954900 L

N-N -1 12.03341600 1.28229500 -10.98845200 L

H-H -1 11.39435100 0.62605200 -11.42469000 L

H-H -1 12.85760200 0.93228100 -10.51558600 L

C-C -1 11.60957200 6.20460800 -11.58213000 L

O-O -1 11.84892200 7.34053400 -11.16089000 L

N-N -1 10.36104600 5.73030000 -11.61983600 L

H-H -1 10.22909900 4.75149700 -11.86484400 L

C-CT -1 9.15999200 6.52994100 -11.33441000 L

H-H1 -1 9.37281400 7.56991900 -11.58339500 L

C-CT -1 7.99994700 6.07654800 -12.23356400 L

H-HC -1 7.80650600 5.02308300 -12.04906400 L

H-HC -1 7.10689600 6.64667800 -11.97780000 L

C-CT -1 8.29400800 6.27944700 -13.72482400 L

H-H1 -1 8.63015500 7.30574200 -13.87324800 L

H-H1 -1 9.10586900 5.61464900 -14.02423900 L

S-S -1 6.87798100 5.99972500 -14.82612900 L

C-CT -1 6.61945400 4.21473800 -14.62663300 L

H-H1 -1 6.23530800 4.00995100 -13.62851200 L

H-H1 -1 5.89652000 3.86519400 -15.36374500 L

H-H1 -1 7.56201500 3.68295700 -14.76100000 L

C-C -1 8.74472300 6.49857500 -9.85404900 L

O-O -1 8.98625800 5.52634500 -9.13806800 L

N-N -1 8.07432800 7.56335700 -9.39951600 L

H-H -1 7.88230500 8.31845200 -10.04102500 L

C-CT -1 7.59038700 7.72501900 -8.02126100 L

H-H1 -1 7.79001300 6.81172600 -7.46282300 L

C-CT -1 8.35375200 8.87371200 -7.32888900 L

H-HC -1 8.06634100 9.81111600 -7.80350500 L

H-HC -1 8.03550700 8.91825100 -6.28681400 L

C-CT -1 9.89150800 8.78157100 -7.36301300 L

H-HC -1 10.23569300 8.73722700 -8.39689800 L

C-CT -1 10.49102800 10.03571900 -6.73198700 L

H-HC -1 10.21235400 10.10015400 -5.67969900 L

H-HC -1 11.57568900 9.99561800 -6.81554500 L

H-HC -1 10.13241300 10.92433800 -7.25073800 L

C-CT -1 10.43612200 7.56735400 -6.60999900 L

H-HC -1 10.10025700 6.65132200 -7.09073400 L

H-HC -1 11.52532200 7.58220000 -6.62059800 L

H-HC -1 10.08914100 7.58126400 -5.57874600 L

C-C -1 6.06676200 7.95145800 -7.98136100 L

O-O -1 5.48062900 8.41816400 -8.96116000 L

N-N -1 5.42962400 7.64902400 -6.85019400 L

H-H -1 5.97326000 7.24938400 -6.08976400 L

C-CT -1 3.98710100 7.77964100 -6.62385900 L

H-H1 -1 3.57648000 8.51646300 -7.31543300 L

C-CT -1 3.34728400 6.41850200 -6.92129200 L

H-HC -1 3.72394800 5.66978600 -6.22518700 L

H-HC -1 2.26682800 6.48973100 -6.82713300 L

H-HC -1 3.58966000 6.11407500 -7.93764400 L

C-C -1 3.67446000 8.26103500 -5.18966100 L

O-O -1 4.48391800 8.05081300 -4.28446500 L

N-N -1 2.52646000 8.91695800 -4.97239300 L

H-H -1 1.87080100 9.03200700 -5.74096200 L

C-CT -1 2.15169700 9.45107900 -3.65185000 L

H-H1 -1 2.57853200 8.79098000 -2.89428800 L

C-CT -1 2.77592500 10.84354400 -3.45010800 L

H-HC -1 3.84879300 10.78480500 -3.63848200 L

H-HC -1 2.34929600 11.53495100 -4.17780900 L

C-CA -1 2.57690200 11.39146300 -2.04719000 L

C-CA -1 1.63739600 12.41522000 -1.81234100 L

H-HA -1 1.08071400 12.84245700 -2.63295700 L

C-CA -1 1.39253300 12.85737000 -0.49713000 L

H-HA -1 0.65307900 13.61367300 -0.29588900 L

C-C -1 2.08040600 12.27647600 0.58976400 L

O-OH -1 1.83326800 12.71503600 1.85048800 L

H-HO -1 2.32558800 12.21285700 2.51825300 L

C-CA -1 3.02174200 11.25184800 0.35353800 L

H-HA -1 3.54171200 10.79558600 1.18367400 L

C-CA -1 3.26820500 10.81191500 -0.96240000 L

H-HA -1 3.98004400 10.01384300 -1.12890100 L

C-C -1 0.63403400 9.48686500 -3.38478300 L

O-O -1 -0.17993500 9.67198100 -4.29389900 L

N-N 0 0.28092100 9.33491000 -2.10273600 L

H-H 0 1.01981500 9.21957200 -1.42383900 L

C-CT 0 -1.07256100 9.22262400 -1.58657500 L

H-H1 0 -1.71512200 8.78526600 -2.35292200 L

C-CT 0 -1.00166800 8.25791800 -0.39513100 L

H-HC 0 -0.52533000 7.34710900 -0.73772000 L

H-HC 0 -0.37740600 8.68750500 0.39075600 L

C-C 0 -2.34534800 7.85898500 0.20003900 L

O-O 0 -3.40214700 8.32737100 -0.21572800 L

N-N 0 -2.29096600 6.96451800 1.18823800 L

H-H 0 -3.14182400 6.64617700 1.62866500 L

H-H 0 -1.39841300 6.60420900 1.49467800 L

C-C 0 -1.62174700 10.59097000 -1.16095300 L

O-O 0 -1.13262400 11.18651700 -0.20054700 L

N-N -1 -2.68187300 11.04647000 -1.84596800 L

H-H -1 -3.00375300 10.49657500 -2.63537900 L

C-CT -1 -3.49877200 12.19485600 -1.45369800 L

H-H1 -1 -2.86332200 12.91893700 -0.94242300 L

C-CT -1 -4.05491800 12.85855100 -2.72729600 L

H-H1 -1 -4.53812700 12.09611200 -3.33670000 L

H-H1 -1 -4.80286800 13.60142800 -2.44534900 L

S-SH -1 -2.76282700 13.68419200 -3.70479400 L

H-HS -1 -2.77391800 14.81823900 -2.97673700 L

C-C -1 -4.60536000 11.77895300 -0.45028800 L

O-O -1 -5.78715600 12.03111300 -0.69366800 L

N-N 0 -4.23017500 11.14292200 0.67049500 L

H-H 0 -3.24486300 10.99205500 0.83367300 L

C-CT 0 -5.15477700 10.45003000 1.56424800 L

H-H1 0 -5.66916900 9.68783300 0.98117500 L

C-CT 0 -4.36195700 9.74217900 2.66268100 L

H-H1 0 -3.63083200 9.06861000 2.22643900 L

H-H1 0 -3.84398900 10.47411400 3.28014800 L

O-OH 0 -5.24837800 9.00425100 3.47057600 L

H-HO 0 -5.69536700 8.35716800 2.92004500 L

C-C 0 -6.15520900 11.40252800 2.23615600 L

O-O 0 -5.71480200 12.35575000 2.87238000 L

N-N 0 -7.47391700 11.12128600 2.19735600 L

C-CT 0 -8.12360700 10.14816300 1.33122000 L

H-H1 0 -8.03290800 9.15395600 1.77005800 L

H-H1 0 -7.70307600 10.15727600 0.32639000 L

C-CT 0 -9.58947200 10.57438100 1.29532300 L

H-HC 0 -10.25720600 9.75510700 1.03521300 L

H-HC 0 -9.71604400 11.40210800 0.59598500 L

C-CT 0 -9.80225200 11.07316100 2.72190200 L

H-HC 0 -9.93182400 10.21932400 3.39003600 L

H-HC 0 -10.65537300 11.74875100 2.79135400 L

C-CT 0 -8.48119700 11.79170200 3.02449100 L

H-H1 0 -8.56135200 12.82346300 2.67872000 L

C-C 0 -8.17962500 11.79898800 4.53671000 L

O-O 0 -8.71351500 12.64866700 5.24914000 L

N-N 0 -7.33055900 10.87322500 5.01774900 L

H-H 0 -6.93638400 10.20947700 4.36651400 L

C-CT 0 -6.88551500 10.75076900 6.40584500 L

H-H1 0 -7.77391100 10.66264100 7.03273900 L

C-CT 0 -6.05477900 9.47232700 6.58575900 L

H-H1 0 -5.14628300 9.54314300 5.98925300 L

H-H1 0 -5.77124900 9.37487600 7.63395900 L

O-OH 0 -6.77958200 8.31952500 6.20810900 L

H-HO 0 -6.22120000 7.55053700 6.34400600 L

C-C 0 -6.07004200 11.95568700 6.90698300 L

O-O 0 -5.99258900 12.14017700 8.12216900 L

N-N -1 -5.47589400 12.76921900 6.01279000 L

H-H -1 -5.68724100 12.62234800 5.03342200 L

C-CT -1 -4.87390500 14.05095200 6.40310800 L

H-H1 -1 -4.35412500 13.91935800 7.35310500 L

C-CT -1 -3.82803700 14.51262200 5.37798500 L

H-HC -1 -4.32938400 14.69438400 4.43165900 L

H-HC -1 -3.41409200 15.46590000 5.70943000 L

C-CA -1 -2.67049300 13.55786100 5.14297200 L

C-CA -1 -1.67902100 13.38922800 6.13002800 L

H-HA -1 -1.74045000 13.92910400 7.06291000 L

C-CA -1 -0.60005500 12.51734700 5.90309700 L

H-HA -1 0.15424200 12.37285400 6.66153700 L

C-CA -1 -0.49767400 11.82282600 4.68521400 L

H-HA -1 0.33460600 11.15175200 4.51346300 L

C-CA -1 -1.47777700 11.99543600 3.69404500 L

H-HA -1 -1.38823500 11.46235200 2.75572400 L

C-CA -1 -2.56456800 12.85714000 3.92500200 L

H-HA -1 -3.31486400 12.98335400 3.16091800 L

C-C -1 -5.95773100 15.12305000 6.60233400 L

O-O -1 -6.86539600 15.27090200 5.78329300 L

N-N -1 -5.84886300 15.93692600 7.65867000 L

H-H -1 -5.08256600 15.79873600 8.29763600 L

C-CT -1 -6.75386500 17.07137000 7.88318900 L

H-H1 -1 -7.73921600 16.78099600 7.52184500 L

C-CT -1 -6.88386400 17.33241600 9.40007200 L

H-HC -1 -7.18102200 16.40563500 9.89101100 L

H-HC -1 -5.91849300 17.64341000 9.79954200 L

C-C -1 -7.91629900 18.38942700 9.77656900 L

O-O -1 -7.73437000 19.17168300 10.69651200 L

N-N -1 -9.06498700 18.42410100 9.14387900 L

H-H -1 -9.71438900 19.14631900 9.39550500 L

H-H -1 -9.29552100 17.75270600 8.42188300 L

C-C -1 -6.31087000 18.27118000 7.02128400 L

O-O -1 -5.72816900 19.23545600 7.52127000 L

N-N -1 -6.51925700 18.16052400 5.70201100 L

H-H -1 -6.95351100 17.29894600 5.38802000 L

C-CT -1 -5.84132300 18.96388200 4.67440800 L

H-H1 -1 -4.78988500 18.67076000 4.66815000 L

C-CT -1 -6.39628500 18.63796600 3.27846700 L

H-HC -1 -7.48647600 18.62030100 3.31131600 L

H-HC -1 -6.10452500 19.43681500 2.59472400 L

C-C* -1 -5.88739600 17.35267000 2.69578100 L

C-CW -1 -6.47084200 16.14101100 2.82967400 L

H-H4 -1 -7.38861100 15.94270500 3.37126000 L

N-NA -1 -5.68060900 15.17603700 2.23887100 L

H-H -1 -5.86392800 14.17527700 2.32290400 L

C-CN -1 -4.53348800 15.71326100 1.70566100 L

C-CA -1 -3.41310500 15.14166400 1.08472900 L

H-HA -1 -3.34038900 14.07067800 0.98465300 L

C-CA -1 -2.37953600 15.97766600 0.63447200 L

H-HA -1 -1.49864700 15.54917600 0.17283100 L

C-CA -1 -2.48788500 17.37051500 0.79530800 L

H-HA -1 -1.68681100 18.00687800 0.44343600 L

C-CA -1 -3.60512900 17.93162500 1.45172300 L

H-HA -1 -3.65535800 18.99984400 1.60665100 L

C-CB -1 -4.65265600 17.11633800 1.93715300 L

C-C -1 -5.84798900 20.47303900 4.92946700 L

O-O -1 -4.77725600 21.06307200 4.87064300 L

N-N -1 -0.67055200 21.35426900 -5.83148700 L

H-H -1 -1.41682500 21.79446300 -5.30736300 L

C-CT -1 0.01655200 20.23756200 -5.16360100 L

H-H1 -1 0.92285800 20.62705800 -4.69614000 L

C-CT -1 -0.90834200 19.72517000 -4.04594800 L

H-HC -1 -1.18081300 20.57251600 -3.41923300 L

H-HC -1 -1.83034900 19.34294100 -4.48729900 L

C-CA -1 -0.31723800 18.66468200 -3.13737100 L

C-CA -1 0.53384700 19.04635400 -2.08334500 L

H-HA -1 0.77127900 20.08846500 -1.92485700 L

C-CA -1 1.08362900 18.07193700 -1.23177000 L

H-HA -1 1.74696100 18.36598900 -0.42910400 L

C-CA -1 0.77535400 16.71460600 -1.42309300 L

H-HA -1 1.20994400 15.96817900 -0.77131000 L

C-CA -1 -0.09888700 16.33245000 -2.45529000 L

H-HA -1 -0.34254300 15.28809900 -2.59303500 L

C-CA -1 -0.64283900 17.30562900 -3.31431300 L

H-HA -1 -1.31990300 17.00557500 -4.09947400 L

C-C -1 0.45266700 19.09818100 -6.10290200 L

O-O -1 1.63385100 18.75553100 -6.15604100 L

N-N -1 -0.46501500 18.54290700 -6.90548200 L

H-H -1 -1.41896600 18.86847100 -6.84978800 L

C-CT -1 -0.12937800 17.42084200 -7.79312800 L

H-H1 -1 0.42170900 16.68578700 -7.20510100 L

C-CT -1 -1.39049300 16.72444300 -8.32657900 L

H-HC -1 -2.02975000 17.44511600 -8.83891300 L

H-HC -1 -1.05077500 15.99006500 -9.05518100 L

C-CT -1 -2.20042200 15.97323300 -7.25455800 L

H-HC -1 -1.51121700 15.45632500 -6.58610000 L

H-HC -1 -2.77916800 16.68261500 -6.66353200 L

C-C -1 -3.14614500 14.92790600 -7.85727800 L

O-O -1 -3.40067500 14.87526900 -9.05449200 L

N-N -1 -3.66945600 14.02521900 -7.05892500 L

H-H -1 -4.14427400 13.25027200 -7.49534000 L

H-H -1 -3.46187300 14.02060600 -6.07087000 L

C-C -1 0.79753000 17.79725300 -8.96593800 L

O-O -1 1.54385600 16.92878700 -9.41973000 L

N-N -1 7.08811600 12.69838900 -9.24296300 L

H-H -1 6.97250600 13.66588400 -8.96671700 L

C-CT -1 5.98904300 11.77905500 -8.94613000 L

H-H1 -1 6.38911400 10.77495900 -8.82577500 L

C-CT -1 5.31874000 12.16598600 -7.62448400 L

H-HC -1 4.88852300 13.15629700 -7.73767900 L

H-HC -1 4.50152400 11.46919000 -7.43738900 L

C-CA -1 6.22951000 12.15577000 -6.41196500 L

C-CA -1 6.20509800 11.06184700 -5.52719100 L

H-HA -1 5.53688500 10.23559000 -5.70662600 L

C-CA -1 7.03845200 11.04370400 -4.39651300 L

H-HA -1 7.00278500 10.20383600 -3.71412100 L

C-CA -1 7.90685100 12.11879900 -4.15130100 L

H-HA -1 8.54581300 12.10838800 -3.27934600 L

C-CA -1 7.93551900 13.21604200 -5.03034200 L

H-HA -1 8.60163700 14.04458100 -4.83480200 L

C-CA -1 7.09181200 13.23961100 -6.15351300 L

H-HA -1 7.10486900 14.09414000 -6.81348000 L

C-C -1 4.99391200 11.74955500 -10.11053500 L

O-O -1 4.52707300 12.79729500 -10.56136200 L

N-N -1 4.68848600 10.55508000 -10.62464800 L

H-H -1 5.08218700 9.73488900 -10.17225200 L

C-CT -1 3.86757100 10.36332900 -11.83124800 L

H-H1 -1 3.57222600 11.33852000 -12.20812900 L

C-CT -1 4.69807300 9.67402800 -12.93490500 L

H-HC -1 5.01948400 8.69248300 -12.58328700 L

H-HC -1 4.06209200 9.51937200 -13.80822200 L

C-CT -1 5.94121500 10.47274700 -13.38676200 L

H-HC -1 6.66188300 10.51886000 -12.56980800 L

H-HC -1 6.40448600 9.93935100 -14.21842800 L

C-CT -1 5.59771000 11.90216900 -13.83876100 L

H-HC -1 4.81307100 11.84651900 -14.59451100 L

H-HC -1 5.22787100 12.47292700 -12.99011500 L

C-CT -1 6.79812400 12.65044100 -14.42295300 L

H-HP -1 7.65799400 12.54293500 -13.75679100 L

H-HP -1 7.04402900 12.19534100 -15.38731300 L

N-N3 -1 6.47199500 14.08503900 -14.60898600 L

H-H -1 6.56926100 14.62688100 -13.75265800 L

H-H -1 7.04206700 14.52123200 -15.33141000 L

H-H -1 5.51118600 14.18155900 -14.93101300 L

C-C -1 2.52141500 9.68474300 -11.58124800 L

O-O -1 1.68874400 9.72563900 -12.48142100 L

N-N -1 2.26753800 9.14274400 -10.38868100 L

H-H -1 2.96898400 9.24089000 -9.67008000 L

C-CT -1 0.94521200 8.65482300 -9.98645700 L

H-H1 -1 0.22190400 8.98834000 -10.72227000 L

C-CT -1 0.92921100 7.11915100 -10.01451700 L

H-HC -1 1.36129700 6.78404400 -10.95701000 L

H-HC -1 1.56611400 6.74421100 -9.21552700 L

C-CA -1 -0.45127200 6.49404500 -9.90272700 L

C-CA -1 -1.46236300 6.83392600 -10.82366000 L

H-HA -1 -1.26040800 7.54610600 -11.60893900 L

C-CA -1 -2.73573500 6.24454600 -10.73305100 L

H-HA -1 -3.50717600 6.50712900 -11.44439600 L

C-CA -1 -3.00443000 5.30598800 -9.72270200 L

H-HA -1 -3.98158800 4.84838700 -9.65669100 L

C-CA -1 -1.99677000 4.95885400 -8.80392000 L

H-HA -1 -2.19823000 4.24198900 -8.02083000 L

C-CA -1 -0.72327100 5.54721800 -8.89734800 L

H-HA -1 0.04265600 5.27437800 -8.18539100 L

C-C -1 0.51685900 9.24686800 -8.63210700 L

O-O -1 1.05010200 8.87061500 -7.58657400 L

N-N -1 -0.44204500 10.18133500 -8.65994700 L

H-H -1 -0.84488900 10.41103900 -9.56238000 L

C-CT -1 -0.94926100 10.90178400 -7.48133300 L

H-H1 -1 -0.45334700 10.52889800 -6.58480800 L

C-CT -1 -0.65068700 12.41384000 -7.59092600 L

H-HC -1 -0.98641900 12.79235500 -8.55781300 L

H-HC -1 -1.24284700 12.90035100 -6.81731700 L

C-CT -1 0.80377600 12.85003500 -7.33693300 L

H-HC -1 0.79892700 13.91072500 -7.08575000 L

H-HC -1 1.19507600 12.31214100 -6.47216700 L

C-C -1 1.75764900 12.65230700 -8.51116300 L

O-O -1 2.31551700 11.58939700 -8.70970500 L

N-N -1 2.03210900 13.65529700 -9.31785900 L

H-H -1 2.76683600 13.47504900 -9.99401500 L

H-H -1 1.61151400 14.56696500 -9.23133200 L

C-C -1 -2.45884600 10.64906100 -7.31382300 L

O-O -1 -3.25826000 11.07325500 -8.14993900 L

N-N 0 -2.88892100 9.96855800 -6.23769200 L

H-H 0 -2.21429200 9.67674100 -5.54490200 L

C-CT 0 -4.22510600 9.35365500 -6.15861400 L

H-H1 0 -4.88341800 9.84572000 -6.87228200 L

C-CT 0 -4.11673200 7.87285800 -6.56219400 L

H-HC 0 -5.08205400 7.38686300 -6.41157900 L

H-HC 0 -3.89406000 7.82164000 -7.62921800 L

C-CA 0 -3.04206000 7.10746400 -5.80891100 L

C-CA 0 -1.71235600 7.14386400 -6.26808100 L

H-HA 0 -1.46538500 7.68329100 -7.16960400 L

C-CA 0 -0.69675200 6.49449800 -5.55179200 L

H-HA 0 0.31966900 6.53082000 -5.90286800 L

C-CA 0 -1.00440300 5.79996200 -4.37457500 L

H-HA 0 -0.22077900 5.29359600 -3.83810700 L

C-CA 0 -2.32649700 5.76689900 -3.90457400 L

H-HA 0 -2.54916200 5.23982400 -2.99094100 L

C-CA 0 -3.34822400 6.41444500 -4.62288200 L

H-HA 0 -4.36473700 6.38717300 -4.25891000 L

C-C 0 -4.87227500 9.49245700 -4.77464000 L

O-O 0 -4.17908400 9.42619600 -3.76177600 L

N-N -1 -6.21113800 9.63992400 -4.74348600 L

H-H -1 -6.71354500 9.54118700 -5.61995600 L

C-CT -1 -6.99391100 9.77972200 -3.49563400 L

H-H1 -1 -6.32660200 10.09617100 -2.69022300 L

C-CT -1 -8.09451700 10.86445700 -3.61132600 L

H-HC -1 -8.81544400 10.53895200 -4.35878600 L

C-CT -1 -8.83621800 11.02121400 -2.26808300 L

H-HC -1 -8.16915000 11.46054000 -1.52612800 L

H-HC -1 -9.70365900 11.66830700 -2.39000900 L

H-HC -1 -9.19326500 10.06064600 -1.89923200 L

C-CT -1 -7.50216300 12.22245500 -4.05823500 L

H-HC -1 -6.73036300 12.51630900 -3.35027300 L

H-HC -1 -7.03972500 12.10639700 -5.03931500 L

C-CT -1 -8.50424200 13.38038000 -4.15757500 L

H-HC -1 -8.84509400 13.67336600 -3.16458300 L

H-HC -1 -8.01685700 14.23758900 -4.62055100 L

H-HC -1 -9.36005600 13.08916200 -4.76458500 L

C-C -1 -7.57139300 8.41285800 -3.10905000 L

O-O -1 -8.56396000 7.96040700 -3.68324300 L

N-N 0 -6.94229900 7.74919900 -2.14698400 L

H-H 0 -6.15185900 8.19292500 -1.70146100 L

C-CT 0 -7.17200000 6.34976100 -1.80163200 L

H-H1 0 -6.99546100 5.75285400 -2.69747300 L

C-CT 0 -6.14555100 5.94002000 -0.74768700 L

H-H1 0 -6.45418400 4.99105600 -0.31235900 L

C-CT 0 -4.75618200 5.76834700 -1.36441600 L

H-HC 0 -4.45035200 6.68035300 -1.87471400 L

H-HC 0 -4.02735000 5.52652200 -0.59551800 L

H-HC 0 -4.77881300 4.95597500 -2.08145100 L

O-OH 0 -6.10868400 6.93022400 0.25529300 L

H-HO 0 -5.47606900 6.66152500 0.92551600 L

C-C 0 -8.58638900 6.01754000 -1.30289000 L

O-O 0 -9.12719100 4.99593200 -1.72093100 L

N-N 0 -9.15867200 6.81019300 -0.38161500 L

H-H 0 -8.67027000 7.63930200 -0.07461900 L

C-CT 0 -10.37920300 6.44254100 0.34729000 L

H-H1 0 -10.55378000 5.37332200 0.22937300 L

C-CT 0 -10.19122900 6.70379900 1.85705900 L

H-HC 0 -10.20251000 7.77696000 2.03316400 L

H-HC 0 -11.04782400 6.27439200 2.37957800 L

C-CT 0 -8.90593200 6.13495000 2.49114900 L

H-HC 0 -8.04049400 6.65703100 2.08551400 L

C-CT 0 -8.93547700 6.38859300 4.00603800 L

H-HC 0 -9.79183700 5.88478700 4.45582500 L

H-HC 0 -8.02052800 6.01440700 4.46497200 L

H-HC 0 -9.00912500 7.45964600 4.19907600 L

C-CT 0 -8.75220300 4.63675100 2.20596600 L

H-HC 0 -8.56912300 4.46828800 1.14626600 L

H-HC 0 -7.90510700 4.23757100 2.76000700 L

H-HC 0 -9.66252200 4.11389300 2.49716700 L

C-C 0 -11.62924300 7.16000300 -0.19412400 L

O-O 0 -12.66092700 7.18147700 0.47697300 L

N-N -1 -11.54260900 7.72550500 -1.40754500 L

H-H -1 -10.66411200 7.61037600 -1.89323500 L

C-CT -1 -12.51680400 8.66217300 -1.97699200 L

H-H1 -1 -12.51273900 9.56671300 -1.36767100 L

C-CT -1 -12.04797900 9.03806100 -3.38975500 L

H-HC -1 -12.02466900 8.15393700 -4.02790100 L

H-HC -1 -12.73429700 9.77002100 -3.81996800 L

H-HC -1 -11.05049300 9.47435300 -3.35212500 L

C-C -1 -13.96907500 8.13988600 -1.98777500 L

O-O -1 -14.85900000 8.79037700 -1.43881900 L

N-N -1 -14.20655400 6.96571700 -2.58204400 L

H-H -1 -13.43361600 6.48709200 -3.02976200 L

C-CT -1 -15.54050400 6.38359900 -2.74857800 L

H-H1 -1 -16.14639600 7.03548700 -3.37789300 L

H-H1 -1 -15.43573800 5.41802200 -3.24375700 L

C-C -1 -16.27891300 6.15513900 -1.43052700 L

O-O -1 -17.44496500 6.52083600 -1.31231100 L

N-N -1 -15.59809200 5.61307500 -0.41965700 L

H-H -1 -14.66703800 5.26386000 -0.61716100 L

C-CT -1 -16.17625400 5.32610800 0.89406800 L

H-H1 -1 -17.06173400 4.70449300 0.75273300 L

C-CT -1 -15.16045500 4.52570800 1.72019900 L

H-HC -1 -14.90349400 3.62066600 1.16961900 L

H-HC -1 -14.24687700 5.11214000 1.83540600 L

C-CA -1 -15.66007500 4.11761400 3.09353400 L

C-CA -1 -16.37358500 2.91362300 3.25604900 L

H-HA -1 -16.56041200 2.27505000 2.40289800 L

C-CA -1 -16.84176900 2.53496100 4.52691700 L

H-HA -1 -17.38500100 1.60678700 4.65051100 L

C-CA -1 -16.60093800 3.35943900 5.63927700 L

H-HA -1 -16.95938900 3.06831900 6.61789600 L

C-CA -1 -15.89250900 4.56385900 5.48063500 L

H-HA -1 -15.70714300 5.19963100 6.33579400 L

C-CA -1 -15.41909200 4.94194100 4.21127500 L

H-HA -1 -14.86929200 5.86725800 4.09893700 L

C-C -1 -16.62970100 6.59824400 1.62478900 L

O-O -1 -17.75491800 6.64520600 2.12430500 L

N-N -1 -15.80530100 7.65246800 1.65991900 L

H-H -1 -14.89503000 7.57746300 1.21793700 L

C-CT -1 -16.18765200 8.91141100 2.31986200 L

H-H1 -1 -16.64154000 8.67836000 3.28460300 L

C-CT -1 -14.94587500 9.76491400 2.60997600 L

H-HC -1 -14.34721300 9.86552400 1.70339300 L

H-HC -1 -15.26831500 10.76201100 2.91447600 L

C-CC -1 -14.09591000 9.20225500 3.72418900 L

N-NB -1 -14.39573500 9.26758500 5.08764600 L

C-CR -1 -13.38904700 8.63113200 5.71382800 L

H-H5 -1 -13.30866000 8.50563900 6.78796600 L

N-NA -1 -12.49078400 8.17689600 4.82390600 L

H-H -1 -11.64674600 7.66742400 5.05795300 L

C-CW -1 -12.92082400 8.53034500 3.56461900 L

H-H4 -1 -12.43675300 8.31912500 2.62449100 L

C-C -1 -17.25592700 9.68160400 1.53098500 L

O-O -1 -18.18203000 10.22156400 2.13659900 L

N-N -1 -20.54242000 7.77588400 2.24489700 L

H-H -1 -19.56917300 7.53517400 2.10238600 L

C-CT -1 -21.02076900 8.05252400 3.60075200 L

H-H1 -1 -21.86746700 7.39127100 3.80101600 L

C-CT -1 -19.93011300 7.72554900 4.63445500 L

H-HC -1 -18.95402600 8.05691700 4.28301500 L

H-HC -1 -20.15272100 8.25238300 5.56336700 L

C-C -1 -19.89997400 6.24333500 4.97009300 L

O-O -1 -20.64908100 5.75844200 5.79918900 L

N-N -1 -19.05703300 5.47277900 4.32860100 L

H-H -1 -19.00294700 4.51090600 4.60825600 L

H-H -1 -18.45613000 5.85957600 3.61153900 L

C-C -1 -21.57930200 9.47800800 3.75297300 L

O-O -1 -22.65856600 9.61747500 4.32476700 L

Mg-MG 0 -4.57879500 -1.57423500 1.01881500 H

C-C -1 -4.98071700 1.06717600 0.66124500 H

O-O -1 -5.19488500 1.05345700 -0.52654600 H

O-O -1 -5.53949100 0.29191300 1.40675200 H

C-C 0 -3.63064900 1.91574300 1.24428000 H

O-O -1 -2.72279900 0.94767400 1.35863000 H

C-CT 0 -4.10556200 2.76997000 2.44235800 H

C-CT -1 -4.54506500 4.14418100 1.93467300 H

C-C -1 -5.04459700 5.18191900 2.92516200 H

O-O2 -1 -4.94149300 6.34375200 2.58048200 H

O-O2 -1 -5.59998600 4.82435700 3.94194800 H

C-C -1 -2.93113400 2.81435500 3.43818500 H

O-O2 -1 -2.27504200 3.82359800 3.54615500 H

O-O2 -1 -2.61260900 1.80742300 4.03077300 H

H-H1 -1 -3.52004400 2.55726200 0.35545900 H

H-HC -1 -4.95391200 2.31078500 2.98749600 H

H-HC -1 -5.34221500 3.98623300 1.21098700 H

H-HC -1 -3.69495400 4.57271300 1.40387400 H

H-H -1 -3.12570100 0.43962500 2.72232700 H

O-O 0 -2.61998800 -1.99084900 1.73436800 H

H-H -1 -1.72267100 -2.13936900 2.10003300 H

H-H -1 -2.51844600 -2.36926600 0.85207000 H

O-O -1 -3.92862900 -0.31957300 3.44826700 H

H-H -1 -4.42186200 0.35733300 4.17265700 H

O-O -1 -5.51471700 -2.31442600 2.65723900 H

H-H -1 -5.74988600 -3.29986300 2.60728300 H

H-H -1 -5.97848700 -2.16746400 3.55281700 H

H-H -1 -3.37717000 -0.91147100 3.96298700 H

**
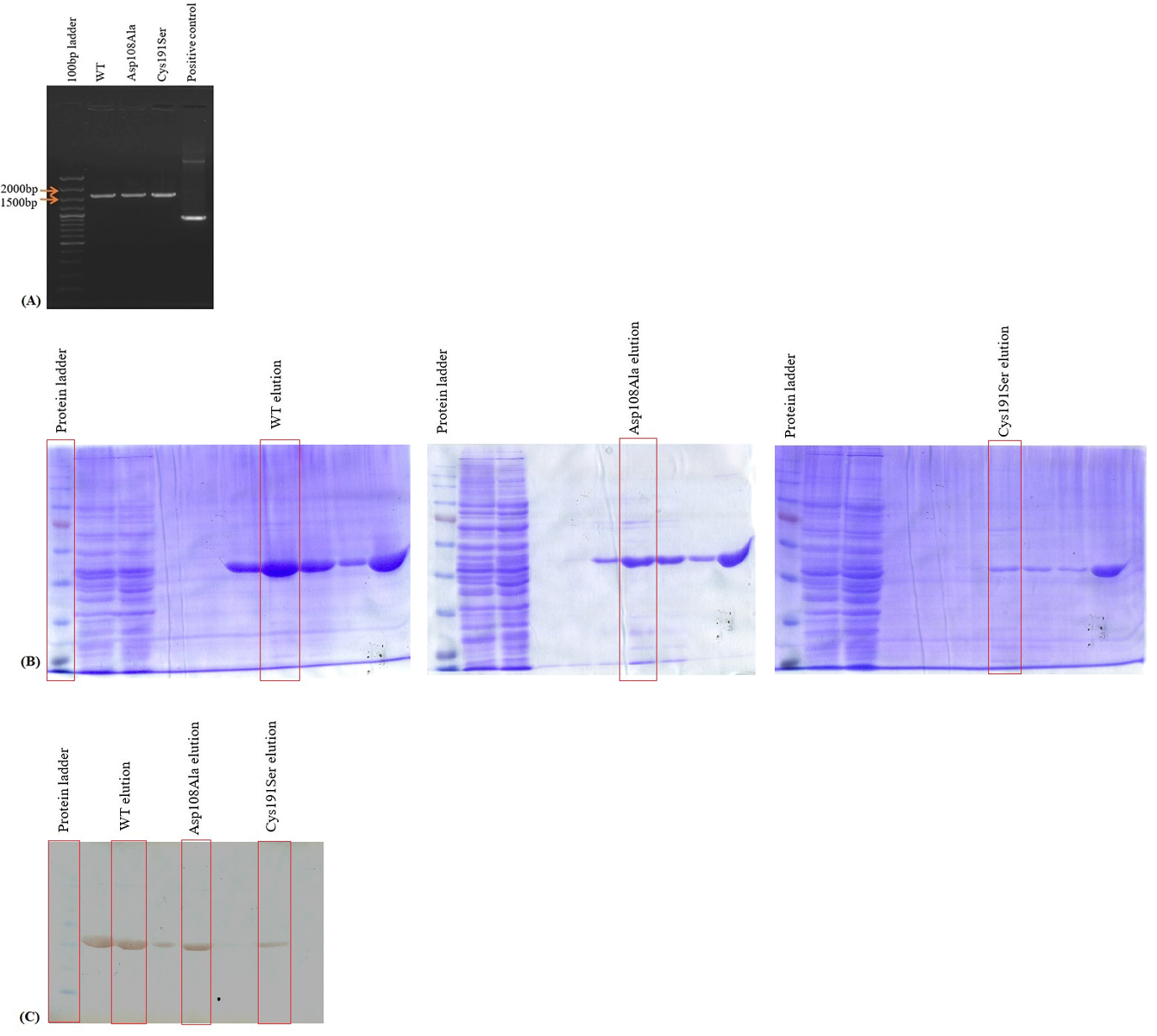
**

**Figure S4.** **(A)** The full-length gel electrophoresis results of colony PCR from wild type (WT), Asp108Ala and Cys191Ser *M. tuberculosis* isocitrate lyase (ICL) gene. The plasmid size for each sample is about 1600bp. **(B)** The Coomasie Blue stained SDS-PAGE full-length gel and **(C)** Western Blot of the purified WT, MT Asp108Ala and Cys191Ser *M. tuberculosis* isocitrate lyase (ICL). The purified ICL is about 50 kDa.
